# A Complex Network of MicroRNAs Expressed in Brain and Genes Associated with Amyotrophic Lateral Sclerosis

**DOI:** 10.1155/2013/383024

**Published:** 2013-07-10

**Authors:** Santosh Shinde, Neelima Arora, Utpal Bhadra

**Affiliations:** Functional Genomics and Gene Silencing Group, Centre for Cellular and Molecular Biology, Hyderabad 500007, India

## Abstract

Amyotrophic Lateral Sclerosis (ALS) is a rare neurological disease affecting mainly motor neurons and often leads to paralysis and death in extreme cases. For exploring the role of microRNAs in genes regulation in ALS disease, miRanda was employed for prediction of target sites of miRNAs expressed in various parts of brain and CNS on 35 genes associated with ALS. Similar search was conducted using TargetScan and PicTar for prediction of target sites in 3′ UTR only. 1456 target sites were predicted using miRanda and more target sites were found in 5′ UTR and CDS region as compared to 3′ UTR. 11 target sites were predicted to be common by all the algorithms and, thus, these represent the most significant sites. Target site hotspots were identified and were recognized as hotspots for multiple miRNAs action, thus, acting as favoured sites of action for the repression of gene expression. The complex interplay of genes and miRNAs brought about by multiplicity and cooperativity was explored. This investigation will aid in elucidating the mechanism of action of miRNAs for the considered genes. The intrinsic network of miRNAs expressed in nervous system and genes associated with ALS may provide rapid and effective outcome for therapeutic applications and diagnosis.

## 1. Introduction

A complete coordination of brain signals and their functional execution in different body parts is often found in multicellular organisms. However, mechanism underlying such coordination between brain and body is still a mystery. How a trivial defect in the coordinated communication can lead to a disease has been a subject of study for many years. Neurological disorders are responsible for major proportion of the global burden of disease [1]. The mammoth rise in incidence of neurological disorders has led to initiatives for declaration of global epidemic. With the significant rise in number of cases of neurological disorders across the globe, it is postulated that no particular geographical region or age group is immune to these disorders. Among these, neurodegenerative disorders that are triggered by environmental and genetic factors are major cause of concern for aged population. Neurodegenerative diseases such as Alzheimer's disease, Parkinson's disease, prion diseases, Amyotrophic Lateral Sclerosis, Huntington's disease, and various spinocerebellar ataxias account for significant morbidity. Progressive and slow degeneration of neurons as witnessed in these diseases leads to neuron dysfunction. The escalating cost of treatment and financial burden in terms of loss in disability adjusted life years (DALYs) along with personal trauma and social stigma warrants the need for exploring new alternatives and novel ways for combating these diseases.

Since its description in 1869 by French neurologist Charcot [2], ALS has become a well-documented motor neuron disease (MND). Amyotrophic Lateral Sclerosis (ALS) is referred to by different names such as motor neuron disease (MND), Charcot's disease, and Lou Gehrig's disease. ALS affects motor neurons that are involved in controlling muscle movement and the disease is characterized by generalized weakness, muscle atrophy, and progressive muscular paralysis, reflecting degeneration of motor neurons in the primary motor cortex, brain stem, and spinal cord [3, 4]. Often sidelined as an orphan disease owing to a small number of cases worldwide, ALS has high lifetime risk despite its low frequency [5].

Many studies have established that the disease pattern has a gender bias as men are more prone to ALS [6–10]. It is also reported that several races are more susceptible to ALS incidence [11]. ALS is a fatal disorder often manifesting itself at a late age. Till date, there is no tangible treatment plan available for ALS [12]. Despite extensive research on ALS, exact mechanism of ALS continues to be a mystery as multiple processes like protein aggregation, ex-cytotoxicity, oxidative stress, and mitochondrial malfunction; inflammation and defects in axonal transport are proposed [13]. About 90% of ALS cases have got no genetic link (sporadic) while the rest are familial [14]. Though highly heterogeneous in nature, clinical symptoms are often relied on for disease diagnosis and degree of severity depends on the number of motor neurons affected. But more often, it is regarded as a disease with obscure pathogenesis due to vast spectrum of clinical indications. Several genes expressed in different pathways are implicated in manifestation of ALS.

It has not been long since the discovery of involvement of a tiny stretch of 21–23 endogenous ribonucleotides called “miRNA” in gene regulation. It is predicted that a considerable proportion of human genes is controlled by the miRNAs [15]. MicroRNAs, often propounded as being “micro in size, macro in function” [16], have been found to be implicated in various vital processes. The wide range of processes, which are reported to be regulated by miRNAs, are cell differentiation, development, stress resistance [17], and several processes in neurodegenerative disorders [18–20]. Growing evidence about the role of microRNAs in neurodegenerative disorders has incited innumerable studies across the globe. Present era has witnessed a revolution in information technology and the avalanche of information arising from high throughput methods has warranted the need for employing *in silico* methods for obtaining fast, simple, and accurate results. Many computational algorithms and tools available for prediction of miRNA and their target sites have led to unfathomable progress in this field [21, 22]. Although computational analysis suffers from drawbacks like high signal to noise ratio yet high speed of prediction and analysis makes it advantageous for initial screening. Besides being skill intensive and time consuming, the conventional methods used for biochemical profiling of miRNA expression are also marred by problems arising due to transient and low level of microRNA expression, specificity to tissue type, and complex interaction of miRNAs and their targets [23]. Annotation and analysis of sites of action of miRNAs provide important clues with regard to their function and mode of action. Comprehensive information about multifarious association of miRNAs with genes of ALS pathway is not readily available. Ample number of miRNAs expressed in brain and central nervous system anticipated to play a part in regulation of coordination of nervous system adds a layer of complexity in gene regulation. Here, we identified targets of microRNAs predominantly expressed in brain on the gene associated with ALS. A diagram depicting a complex interaction between microRNAs and genes was constructed which will aid in deciphering the role of miRNAs in ALS etiology. This study offers sufficient information regarding the regulation of ALS genes, which represents first effort for simplification of microRNA-based control of ALS genes. It also sheds ample light on the complex interplay of genes and their targeted miRNAs.

## 2. Materials and Methods

**Figure 1:**
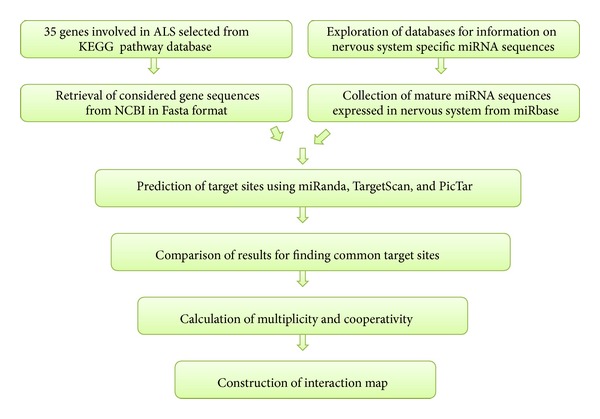
Schematic representation of the workflow.

In current study, we emphasize on identification of fraction of miRNA targets specific to the pathway involved in ALS and propose an interaction network between these genes and miRNA. Figure 1 represents stepwise workflow of the study.

### 2.1. Selection of Genes

Genes involved in ALS manifestation contribute in a unique pathway in a synchronized manner and are referred to in KEGG (Kyoto Encyclopedia of Genes and Genomes) [24] pathway. The KEGG pathway database is an excellent repository that provides biochemical and genetic information related to pathways in a sequential manner in form of diagrams depicting associations, molecular interactions, and reactions. KEGG (http://www.genome.jp/kegg/) was explored for information on genes involved in ALS. The ALS pathway represented in KEGG consists of 35 genes present on different chromosomes.

The genes considered were *SLC1A2 solute carrier family 1 (glial high affinity glutamate transporter), member 2, GRIA1, TNF tumor necrosis factor, tumor necrosis factor receptor superfamily member 1A, caspase 1, superoxide dismutase 1, tumor protein p53, CHP calcium binding protein P22, BID BH3 interacting domain death agonist, TOMM40 translocase of outer mitochondrial membrane 40 homolog, BCL2 B-cell CLL/lymphoma 2, BCL2-like 1, BAX BCL2-associated X protein, BAD BCL2-associated agonist of cell death, cytochrome c somatic, apoptotic peptidase activating factor 1, caspase 9, caspase 3, Der1-like domain family member 1, MAP3K5, DAXX death-domain associated protein, MAP2K 3, MAP2K6, MAPK14, nitric oxide synthase 1, copper chaperone for superoxide dismutase, glutathione peroxidase 1, catalase, neurofilament, light polypeptide, NEFM neurofilament, medium polypeptide, NEFH neurofilament, heavy polypeptide, peripherin, Amyotrophic Lateral Sclerosis 2, RAB5A,* and *ras-related C3 botulinum toxin substrate 1.GPX1* and *RAB5A* are located on chromosome 3 while *TNF*, *MAP3K5*, *DAXX* death-domain associated protein and *MAPK14* are located on chromosome *6. CYCS* and *RAC1* are positioned on chromosome 7 and *DERL1*, *NEFL*, and *NEFM* are sited on chromosome 8. Five genes, namely, *SLC1A2*, *CASP1*, *BAD*, *CCS*, and *CAT*,are present on chromosome 11 while *TNFRSF1A*, *APAF1*, *NOS1*, and *PRPH* reside on chromosome 12. Detailed information of each gene is summarized in Table S1 (in Supplementary Material Available online at http://dx.doi.org/10.1155/2013/383024). Gene sequences were retrieved in Fasta format (a widely acceptable format) from public database NCBI.

### 2.2. Selection of miRNA Dataset

Information regarding specific microRNAs expressed in midbrain, cerebellum, hippocampus, and frontal cortex of human was collected from the Human microRNA and Disease Database (HMDD) http://202.38.126.151/hmdd/mirna/md/), (mir2disease data base (http://www.mir2disease.org/), and other relevant references. Mature miRNA sequences were collected from miRbase (http://www.mirbase.org/) [25]. The number of miRNAs expressed in cerebellum, hippocampus, midbrain, and frontal cortex available in public repository is 62, 93, 112, and 105, respectively. Out of these, few miRNAs are not only limited to one region but are expressed in other parts also. Expression of 23 miRNAs is limited only to midbrain while 17 miRNAs are expressed in hippocampus. The number of miRNAs that are expressed only in frontal cortex and cerebellum is 14 and 4, respectively.

The concept of miRNA target sites finds application in functional annotation. miRanda (version 1.0 b) available at http://www.microrna.org/microrna/releaseNotes.do, a dynamic programming algorithm that relies on the sequence complementarity [26, 27], was employed for prediction of target sites of miRNAs expressed in nervous system on the selected genes. miRanda allots higher weights to matches at the 5′ end of the mature miRNA while considering the free energy of the RNA-RNA duplex [28] and the degree of conservation of the miRNA targets across related genomes. The analysis was performed keeping the following cut-off values for prediction of target sites: Gap Open Penalty: 2.0, Gap Extend: 8.00, match score (*S*) ≥ 150.00, duplex free energy (Δ*G*) = −25.00 kcal/mol, and scaling parameter (*w*) = 3.00. The selected gene sequences and mature miRNAs expressed in human midbrain, cerebellum, hippocampus, and frontal cortex were used as reference and query sequences, respectively, as input to miRanda.

Positions of the target sites for these miRNAs on 35 genes were explored and sites prone to multiple miRNAs were identified as hot spots. Multiplicity and cooperativity were determined on the basis of miRNA and targeted gene interactions. After assembling the data for all genes, miRNAs were selected based on high multiplicity. A complex interaction network among selected genes and top miRNAs (as elucidated by the algorithm) was constructed.

### 2.3. Prediction of Common miRNA Targets by Multiple Programs

Target sites of microRNA predicted solely by miRanda were further verified using TargetScan (Release 5.1) [29]. The predicted miRNA target binding sites in the 3′ UTR were also compared using PicTar [30], which predicts miRNA target sites based on the conservation. miRNA targets only in 3′ UTR that are common in these miRNA prediction algorithms and their comparative analysis are provided in Table 4. Based on the intersection of results, microRNAs were selected and multiplicity and cooperativity were determined.

## 3. Results and Discussion

Delicate balance of gene expression involved in a trait is maintained by employing different regulatory mechanisms. One of the important processes is microRNA-mediated gene regulation, which basically fine-tunes the expression of genes essential for the pathway. Prediction of miRNA target sites using bioinformatics approach offers noteworthy advantages such as sequence specificity, rapid selection, and screening procedures. This methodology can be exploited for the recognition of molecular hallmarks of different disease traits. But a major weakness of *in silico* analyses is the lack of experimental validation. For circumventing such problem, a combinatorial approach can be implemented where target sites are predicted using multiple programs. This methodology can verify the potential targets of microRNAs. Further, the sites of miRNA action will aid in identifying the stage of regulation of genes associated with ALS that can be effectively utilized for therapeutic applications.

**Figure 2:**
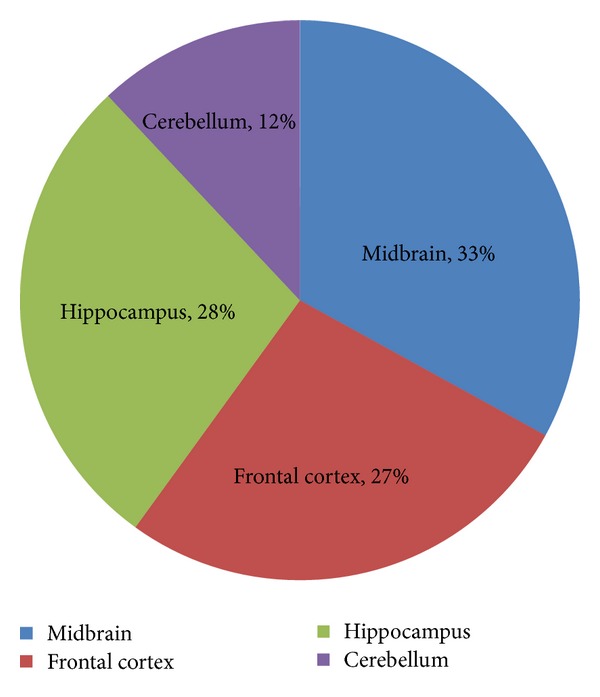
Percentage of miRNAs expressed in various parts of the brain.

**Figure 3:**
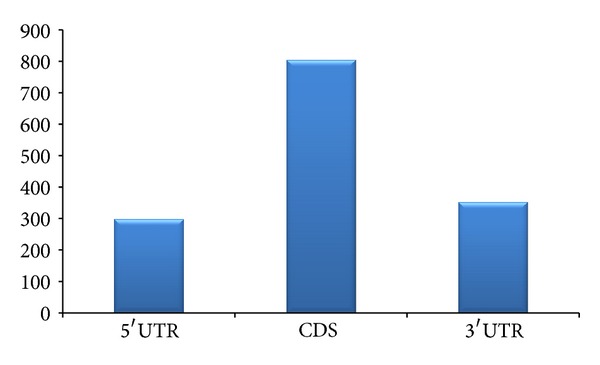
Distribution of target sites in different regions of selected genes for the selected miRNAs.

Out of miRNAs that are expressed in midbrain, cerebellum, hippocampus, and frontal cortex, majority of them are from midbrain (Figure 2) while nearly equal proportions of microRNAs are expressed in hippocampus and frontal cortex. The microRNAs in cerebellum are less in number compared to miRNAs expressed in other regions considered (Figure 2). The target sites of the microRNA expressed in different parts of the brain were predicted on the 35 genes related to ALS for initial screening. The result obtained from miRanda (version 1.0) indicates that 477 target sites were predicted for miRNAs expressed in midbrain as compared to the target sites (411) predicted for miRNAs expressed in hippocampus. However, total target sites predicted for miRNAs expressed in cerebellum and frontal cortexes were 175 and 395, respectively. The number of targets for miRNAs expressed in cerebellum is considerably less as compared to binding sites for miRNAs expressed in other areas of the brain. Surprisingly, microRNA targets were distributed in the different regions of the genes and are not limited to only 3′ UTR. Total 1456 target sites were predicted using miRanda for miRNAs considered in the study. Majority of them were found in CDS. Target sites in the 3′ untranslated region is comparatively less. However, a significant number of targets were also found in the 5′ UTR of the genes (Figure 3). Earlier, the occurrence of miRNA target sites in coding region and 5′ UTR of genes was considered as exception in animals. But numerous recent studies have established that microRNAs can target different regions of the gene and microRNA-based regulation is not confined only to 3′ UTR [31–35]. Experiments aimed at identifying specific parameter or factor for effective targeting of 3′ UTR by miRNAs have failed considerably. Conversely, the existence of miRNA target sites in ORF of Nanog, *OCT4* and *SOX1*, during induction of stem cell pluripotency [36] supports the existence of miRNA binding sites in other sites of the genes in animals. These results established the notion that microRNA target sites are not restricted to 3′ UTR in animals. Such variability in regions also adds cues for anticipating the mode of action specially their role in transcriptional, posttranscriptional, and translation inhibition. As the majority of the algorithms developed for miRNA target site prediction considers only 3′ UTR, verification of 3′ UTR target site prediction using a combinatorial approach will be useful.

**Figure 4:**
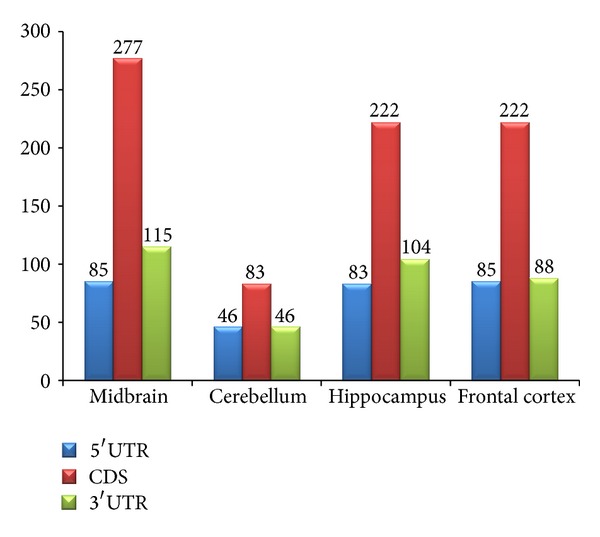
Distribution of target sites in 5′ UTR, CDS, and 3′ UTR for miRNAs expressed in various parts of nervous system.

Target sites on ALS genes were classified according to the microRNAs expressed in four different regions of brain. Maximum target sites were predicted in the CDS of all the genes considered. However, for the miRNAs expressed in midbrain, hippocampus, and frontal cortex, the number of targets in the coding region is threefold greater than the number of miRNA targets found in 5′ UTR and 3′ UTR, but in miRNAs expressed in cerebellum, the number of targets in CDS was about twofold levels as compared to other regions of the genes. As there were less miRNAs expressed in cerebellum, this can explain small number of target sites predicted on genes associated to ALS.  Figure 4 shows the distribution of collective target sites of miRNAs expressed in brain in the various regions of the selected genes.

Next, we analyzed the microRNA targets in each gene associated with the ALS pathways. No target sites could be detected in 3′ UTR for miRNAs expressed in midbrain for *CASP1*, *GRIA1*, *GPX1*, *DAXX*, *CAT*, *SOD1*, *NEFM*, *NEFL,* and *NEFH* genes. Similarly, CDS of two genes, *CYCS* and *RAB5A,* have no potential targets. Conversely, CDS of *MAP3K5* have maximum number of targets for miRNAs expressed in midbrain. On the other hand, 5′ UTR lack any target sites of same miRNAs in *CASP1*, *GRIA1*, *GPX1*, *DAXX*, *CAT*, *SOD1*, *NEFM*, *NEFL*, *NEFH*, *RAC1*, and *TNF* genes. The genes having maximum and minimum number of target sites were also identified. The miRNAs expressed in midbrain showed the least number of target sites (3) in *RAC1* and *CASP1* genes while 29 target sites were predicted in *NOS1*. Similar results were obtained for miRNAs expressed in cerebellum for the same genes. For instance, *RAC1* has only 1 target site whereas *NOS1* has 11 target sites. For miRNAs expressed in hippocampus, *CASP1* and *GRIA1* genes have minimum number of predicted sites (3) and *MAP3K5* was predicted to possess maximum target sites (33). Similarly, *CASP1* and *SOD1* showed 2 target sites each while 23 target sites were predicted for *TNFRSF1A* for miRNAs expressed in frontal cortex. On the contrary, target sites could not be predicted in CDS for 6 genes, namely, *GPX1*, *CYCS*, *CHP*, *CASP3*, *SOD1,* and *RAB5A,* for miRNAs expressed in cerebellum. No target sites could be predicted in 3′ UTR of *ALS2*, *BID*, *CASP1*, *GRIA1*, *DAXX*, *CCS*, *CAT*, *CASP9*, *TOMM40*, *TNF*, *SOD1*, *RAC1*, *NEFM,* and *NEFL* genes. No target site could be predicted in *BCL2*, *CASP1*, *GRIA1*, *DAXX*, *CYCS*, *CASP3*, *TNF*, *RAC1*, *NEFM*, and *NEFH* genes for miRNAs expressed in cerebellum. These results indicate that the majority of the ALS genes lack any target site for miRNAs expressed in cerebellum. Target sites predicted using miRanda are summarized in Table S2 (in Supplementary Material). miRNA targets were found in most of the genes but surprisingly few genes, *CASP1 GRIA1*, *DAXX*, *CAT*, *SOD1*, and *NEFM*, lack any miRNA target site in their 3′ UTR.

### 3.1. Hotspot Identification

MicroRNAs either share the same complementary target sites or proximally located sites adjacent to other miRNA target site. A hot spot can be defined as a stretch of sequence that is prone to action of a group of miRNAs. Many studies have revealed that usually microRNAs having the same origin are regulated and expressed together in time and space [37]. Such miRNAs play extensive role in many vital processes and carry out important biological functions in different pathways [38–40]. It is quite possible that all the miRNA occupying position in “hotspot” may not be equally effective for controlling the same gene. Based on competitive selection, predominant miRNA may outcast other miRNAs and get potentially involved in repression of other microRNA or in similar events. But exact mechanism behind such selection remains unknown. It will be interesting to elucidate the factors responsible for variable effectiveness of microRNAs. Various researchers have used different standards and parameters such as the same chromosomal location for more than two miRNAs, the same orientation, phylogenetic relationships, and absence of interfering transcription unit [41, 42]. As miRNAs are presumed to be implicated in important biological processes, miRNA targets predicted using miRanda were then further analyzed for identifying miRNA-prone regions in all selected genes. We defined a region as hotspot or region prone to multiple miRNA action if the region showed minimum 10 nucleotides overlap from the starting position and concurrence of 3 miRNA targets. Total 11 miRNAs-prone regions were identified in 35 genes associated to ALS (Figure SF1-37 in Supplementary Material). CYCS and MAP2K3 showed 1 hotspot each in 3′ UTR while single target site hotspot was found in 5′ UTR in 4 genes, that is, BAX, DERL1, SLC1A2, and RAB5A. Two such regions were found in CDS of CASP9 and 3 other genes, namely, TNF, MAPK14, and MAP2K3, showed the occurrence of single region vulnerable to multiple miRNAs (Table S7 in Supplementary Material).

**Figure 5:**
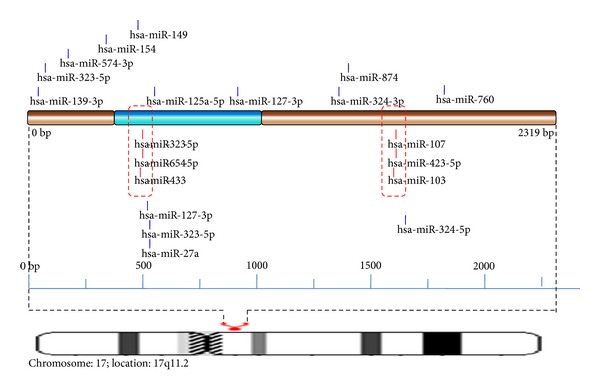
Schematic representation of miRNA targets on MAP2K3 gene.

In miRNA cluster prediction, 2 hot spots were identified in MAP2K3. The hotspot in CDS was found to have target sites for 3 miRNAs,that is, hsa-mir-323-5p, hsa-mir-654-5p, and hsa-mir-433 (Figure 5). Target sites for 3 miRNAs namely hsa-mir-107, hsa-mir-423-5p, and hsa-mir-103 are congregated in another hotspot found in 3′ UTR.

MAP2K3, having 2 such hotspots, is expected to show more sensitivity towards gene regulation by microRNAs. Occurrence of such small number of hot spots indicates the possible specificity of miRNAs and stringency of regulation. The variability in existence of such target site hotspots enhances the complexity in gene regulation. These results suggest that any region in a gene, namely, 5′ UTR, CDS, and 3′ UTR, can be targeted by the microRNAs as opposed to the earlier belief which postulated 3′ UTR as the only site of action of microRNAs.

### 3.2. Multifarious Relationships among Gene and miRNAs

MicroRNA-mediated regulation offers a whole new dimension and often researchers are left confounded by sheer complexity of its level. Not akin to the earlier incidences of one miRNA targeting one gene, distribution of target sites for the miRNAs points out various possibilities where multiplicity and cooperativity come into picture. In principle, one miRNA can target more than one gene (multiplicity), and one gene can be controlled by more than one miRNA (cooperativity) [26]. Multiplicity is a property arising from relaxed base pairing. As reported earlier, single miRNA can control hundreds of genes. Distribution of target sites on genes is rarely uniform and these target sites are often found scattered across a region, and range of occurrence is also known to vary in numbers in a gene [27]. Often, presence of more target sites per gene indicates efficient regulation. In this study, we found multiple target sites in most of the genes. After assembling all data, multiplicity and cooperativity were calculated. From these analyses, selective miRNAs that displayed maximum number of interactions with the genes associated with manifestation of ALS were used for generation of an interaction network (Table 1). We observed that hsa-miR-370 showed maximum multiplicity as it showed 65 interactions with 30 gene sequences while no target site could be predicted in 5 genes, namely, *CASP1*, *GPX1*, *DERL1*, *CCS*, and *SOD1*. Thus, it is expected that degree of repression by hsa-mir-370 will be considerably high. Next, hsa-miR-874 displayed 52 interactions with 29 genes but did not show any target in *CASP1*, *CHP*, *SOD1*, *RAC1*, *RAB5A*, and *MAP2K6* genes. None of the top miRNAs display any interaction with SOD1. Several evidences implicated that SOD1 is one of the core factor for ALS that encodes the free radical scavenging enzyme copper zinc superoxide dismutase in ALS pathway. It can be interpreted that these miRNAs may not be involved in regulation of *SOD1* activity.

In a similar approach, the data was analyzed for estimating cooperativity. Highest cooperativity was observed for *PRPH* followed by *TNFRSF1A* and *TOMM40* towards top 10 microRNAs. The complex picture presented by these interactions is difficult to interpret. For instance, *PRPH* is regulated by 10 miRNAs at 23 positions, so these top 10 miRNAs demonstrate high cooperativity towards *PRPH*. Top 10 miRNAs exhibited 22 and 20 targets for *TNFRSF1A* and *TOMM40* genes. Relatively very low cooperativity was observed in *BID*, *MAP2K6,* and *DERL1* genes. It can be inferred that there exists a low sensitivity of these genes towards microRNA mediated gene regulation. Seemingly, simple process of selection of microRNA targets is endowed with inherent complexity and this led to the development of a complex network by two phenomena, that is, multiplicity and cooperativity. These two properties may serve as deciding factors for the mode of miRNA action, which have significant role in chromatin organization, rapid RNA turnover, or translational inhibition. A complete and detailed analysis is required to unravel microRNA-based gene regulation. Table 1 shows the miRNAs with high multiplicity and cooperativity.

### 3.3. A Simplistic Approach for Complex Interaction

**Figure 6:**
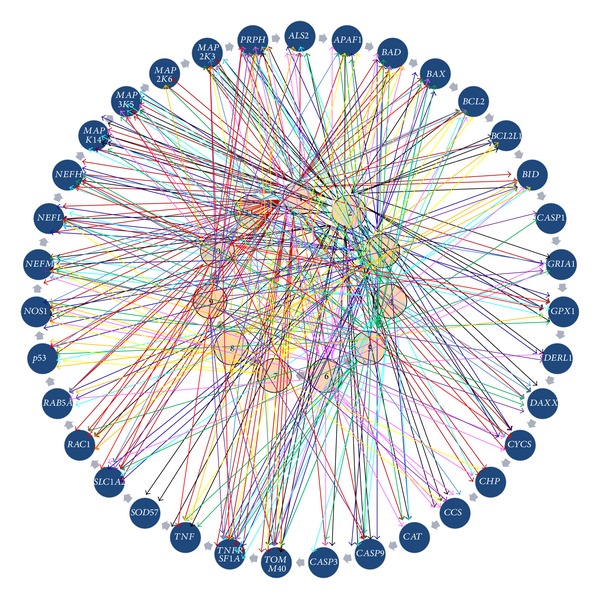
Interaction map of miRNA and selected 35 genes. Interactions among genes and miRNAs are depicted with arrows, where 1 = hsa-miR-370, 2 = hsa-miR-874, 3 = hsa-miR-423-3p, 4 = hsa-miR-323-5p, 5 = hsa-miR-760, 6 = hsa-miR-149, 7 = hsa-miR-139-3p, 8 = hsa-miR-744, 9 = hsa-miR-324-3p, 10 = hsa-miR-339-3p, and 11 = hsa-miR-654-5p.

We employed a simple approach to derive a complex microRNA-ALS gene integrated network for envisaging the relationships shared by top 10 miRNA targeting ALS genes (Figure 6). The major advantage underlying this approach is its simplicity in identifying complete sets of microRNA and their targets on a particular disease at a glance. The highest number of interactions with genes (30) was shown by hsa-miR-370. In contrast, only 4 interactions were observed in *MAP2K6* for hsa-miR-370, hsa-miR-760, and hsa-miR-744 miRNAs. On determining targets on an individual gene in consideration, it was noted that the maximum number of interactions for selective top microRNAs was observed between hsa-miR-370 and *TNFRSF1A* whereas *SOD1* did not show any conclusive interaction with top miRNAs. It can be presumed that *SOD1* may not be under control of the microRNAs. These findings provide an important clue that few genes are devoid of microRNA-based regulation. It might appear that some of the core components of ALS pathway may not be under direct control of miRNAs.

### 3.4. Prediction of Common Targets

**Figure 7:**
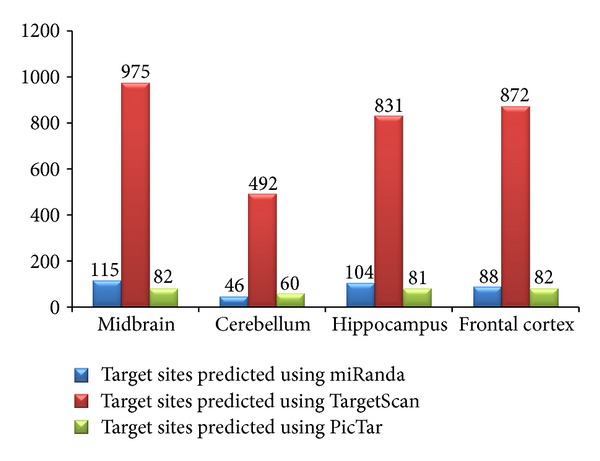
Target sites predicted in 3′ UTR of selected genes using miRanda, TargetScan and PicTar.

To eliminate noise from computational prediction, other algorithms were used for verification of the data obtained from miRanda program. However, major drawback of such verification underlies in the differences in number of predicted targets. It can be attributed to the fact that miRNA target sites in animals are quite small with limited complementarity. A slight variation in prediction algorithms is capable of producing diverse and variable results. As a measure, the convergence or intersection of the results was obtained for ensuring the reliability. Second, most of the algorithms restrict their search only to 3′ UTR; therefore, there is no option available for validating the miRNA targets present in the 5′ UTR and CDS. A comparative analysis of three algorithms, miRanda, TargetScan, and PicTar, is summarized in Figure 7. TargetScan is a well-established tool for identifying complementary sites for miRNA binding which has low false positive rate owing to its dependence on perfect seed complementarity. PicTar recognizes sequences of perfect 7-mer matches starting at initial one or two nucleotides of the 5′ end of the miRNA. TargetScan predicted 3170 target sites while PicTar predicted only 305 targets from miRNA pool considered.

Target sites predicted using miRanda, TargetScan, and PicTar were compared (Figure 7). TargetScan predicted the maximum number of target sites for miRNAs expressed in midbrain while the lowest number of target sites was predicted for miRNAs expressed in cerebellum (Table S3 in Supplementary Material). PicTar predicted considerably less number of sites as compared to TargetScan. The comparative account of total number of sites predicted using these algorithms is summarized in Table 2.

miRNA binding sites could be predicted for only 17 genes using PicTar (Table S4 in Supplementary Material) in the available dataset. *ALS2*, *APAF1*, *BCL2*, *BCL2L1*, *DERL1*, *CHP*, *CASP3*, *TNFRSF1A*, *SLC1A2*, *RAC1*, *RAB5A*, *NEFM*, *NEFH*, *MAPK14*, *MAP3K5*, and *MAP2K7* are the genes for which target sites could be predicted for miRNAs expressed in cerebellum. For miRNAs expressed in hippocampus as well as frontal cortex, PicTar predicted target sites for 17 genes. The number of genes for which miRNA target sites were predicted employing PicTar was 18 in case of microRNAs expressed in midbrain. No target site could be predicted for *DAXX* using TargetScan, while in case of miRanda, 4 target sites were predicted for hsa-miR-204, hsa-miR-874, and hsa-miR-93 expressed in cerebellum.

The number of targets sites predicted by these programs is quite large as compared to common microRNA targets envisaged by three programs. TargetScan predicted a significantly higher number of target sites as compared to those predicted using miRanda. Target sites predicted based on only seed sequence similarity in TargetScan (only in 3′ UTR) are even greater than the 1456 sites (for 3′ UTR, CDS, and 5′ UTR) predicted by miRanda.

**Figure 8:**
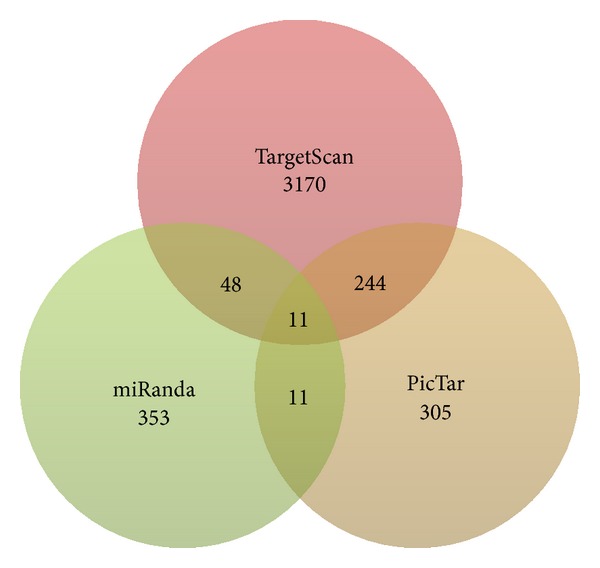
Intersection of miRanda, PicTar, and TargetScan results.

**Figure 9:**
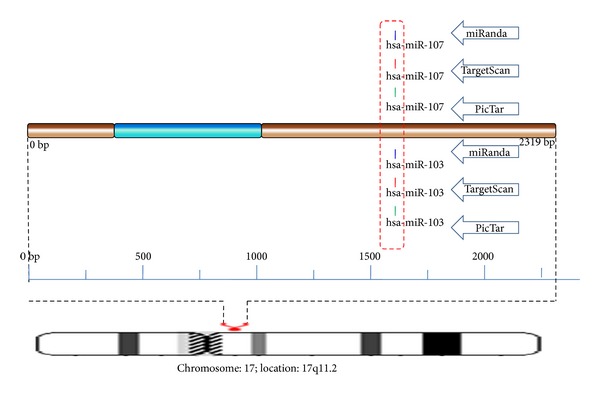
Schematic representation of miRNA conserved target on MAP2K3.

However, scanning for common target sites predicted by miRanda and TargetScan provided only 48 targets (Table 3, Figure 8). TargetScan and PicTar algorithms take into account the conservation criteria and this might be clue for large number of common sites predicted but these algorithms consider only 3′ UTR. Target sites found to be common and conserved in the output obtained from all three programs represent a much smaller number (11).  Figure 9 shows the common target sites predicted on *MAP2K3* by all the programs.

**Figure 10:**
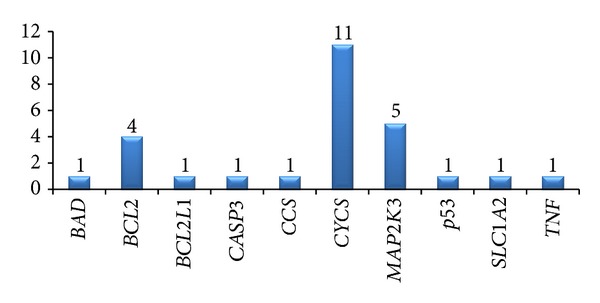
Common target sites predicted using miRanda and TargetScan where data labels show the number of target sites predicted for each gene.

On analyzing the results of TargetScan and miRanda, it was found that for *CYCS* gene, 11 target sites were found to be comparable (Figure 10). hsa-miR-93 (miRanda: 3333–3357, TargetScan: 3348–3354; miRanda: 4143–4167, TargetScan: 4158–4164), hsa-miR-125b (miRanda: 1564–1590, TargetScan: 1582–1588), hsa-miR-17 (miRanda: 3333–3357, TargetScan: 3348–3354; miRanda: 4143–4167, TargetScan: 4158–4164; miRanda: 2189–2212, TargetScan: 2204–2210), hsa-miR-25 (miRanda: 2597–2619, TargetScan: 2610–2616), hsa-miR-92a (miRanda: 2596–2619, TargetScan: 2610–2616), hsa-miR-20a (miRanda: 4143–4167, TargetScan: 4158–4164), hsa-miR-221 (miRanda: 1576–1599, TargetScan: 1592–1598), and hsa-miR-93 (miRanda: 3333–3357, TargetScan: 3348–3354) were found in vicinity. Results obtained for other genes are summarized in Table 3. Similarly, results for sites were found to be quite similar in PicTar versus TargetScan (Table S5 in supplementary material) and miRanda versus PicTar (Table S6 in Supplementary Material).

The number of predicted target sites that converged in all the 3 programs used was found to be quite small. On further exploration, it was found that multiplicity for the common miRNAs that appeared to converge was relatively very less and ranged from 2 to 3.

## 4. Conclusion

Emerging neurodegenerative diseases affect the quality of life and pose huge social-economic burden in terms of treatment and care. Researchers have unveiled the role of miRNAs in regulation of genes in various neurodegenerative disorders. The complexity of neurodegenerative diseases and their predisposing factors (for instance, environment and genetic adaptation) motivated us to explore the confounding relationship of genes and miRNAs involved in ALS in present investigation. Limited knowledge of genes involved in ALS and the exact mode of their regulation is an impediment in devising efficient treatment plan. The major goal of the study was to identify a complete set of microRNAs that interact with genes involved in disease. The constructed network based on the relationship between microRNAs and genes involved in ALS represents a simplified way of looking at the complex interplay involved and may prove useful in identification of microRNA-mediated regulation, genes prone to miRNA action, and their involvement in disease manifestation. Several algorithms have been developed recently for the identification of the microRNA targets in a gene. With the advent of rapid and efficient computational programs, it is possible to predict possible network of miRNA expressed in brain and target ALS genes, which will aid in unraveling their mode of action and pathways for controlling genes of a disease. Present efforts aim to devise a new approach to construct miRNA network for a single trait and disease.

Understanding the mechanism of repression of candidate genes involved in disease onset by a single miRNA or multitude of miRNAs may aid in paving the way for devising a tactical way to control disease. This study provides an overall view of complex and intricate association of miRNAs and genes. Distribution of target sites across the gene along various regions interprets the plausible pathway of gene regulation. Interestingly, more miRNA targets sites were found in CDS and 5′ UTR as compared to 3′ UTR. Sites prone to the action of multiple miRNAs based on their sequence complementarity were identified and explored, as they may be preferred sites for miRNA-mediated control. Information about multiplicity and cooperativity of miRNAs in genes associated with ALS may aid in selection of miRNAs for diagnostics and therapeutics. Though the work sheds light on miRNA action and important miRNAs involved in ALS, it has its own limitations. As the majority of computational tools and servers for miRNA prediction primarily target 3′ UTR, it is difficult to cross-verify miRNA target sites predicted in 5′ UTR and CDS. *In silico* analysis can be supplemented using experimental approach in future. Nevertheless, this combinatorial approach, which makes use of various computational programs for prediction of common microRNA pools, provides a novel way for exploring such relationships and can serve as foundation for further investigations.

## Supplementary Material

Table S1: Information of Amyotrophic Lateral Sclerosis Disease associated genes selected from KEGG pathway database.
Table S2: Distribution of target sites predicted across gene in 5' UTR, CDS and 3' UTR for miRNAs expressed in midbrain, cerebellum, frontal cortex and hippocampus predicted by miRanda.
Table S3: Target sites predicted for miRNAs expressed in midbrain, cerebellum, hippocampus and frontal cortex using TargetScan.
Table S4: Target sites predicted for miRNAs expressed in midbrain, cerebellum, hippocampus and frontal cortex using Pictar.
Table S5: Comparison of target site prediction results obtained using TargetScan and Pictar.
Table S6: Comparison of target site prediction results obtained using miRanda and Pictar.
Table S7: Hot spots identified in selected genes in 5'UTR , CDS and 3' UTR for miRNAs considered in the study.
Figure SF1: Schematic representation of miRNA Target sites on ALS2
Figure SF2: Schematic representation of miRNA Target sites on APAF1
Figure SF3: Schematic representation of miRNA Target sites on BAD
Figure SF4: Schematic representation of miRNA Target sites on BAX
Figure SF 5: Schematic representation of miRNA Target sites on BCL2
Figure SF 6: Schematic representation of miRNA Target sites on BCL2L1
Figure SF7: Schematic representation of miRNA Target sites on BID
Figure SF8: Schematic representation of miRNA Target sites on CASP1
Figure SF8: Schematic representation of miRNA Target sites on CASP1
Figure SF9: Schematic representation of miRNA targets on GRIA1
Figure SF10: Schematic representation of miRNA Target sites on GPX1
Figure SF11: Schematic representation of miRNA Target sites on DERL1
Figure SF12: Schematic representation of miRNA Target sites on DAXX
Figure SF13: Schematic representation of miRNA Target sites on CYCS
Figure SF14. Schematic representation of miRNA targets on CHP
Figure SF15: Schematic representation of miRNA Target sites on CCS
Figure SF16: Schematic representation of miRNA Target sites on CAT
Figure SF17: Schematic representation of miRNA Target sites on CASP9
Figure SF18: Schematic representation of miRNA Target sites on CASP3
Figure SF19: Schematic representation of miRNA Target sites on TOMM40
Figure SF20: Schematic representation of miRNA Target sites on TNFRSF1A
Figure SF21: Schematic representation of miRNA Target sites on TNF
Figure SF22: Schematic representation of miRNA Target sites on SOD1
Figure SF23: Schematic representation of miRNA Target sites on SLC1A2
Figure SF24: Schematic representation of miRNA Target sites on RAC1
Figure SF25: Schematic representation of miRNA Target sites on RAB5A
Figure SF26: Schematic representation of miRNA Target sites on p53
Figure SF27: Schematic representation of miRNA Target sites on NOS1
Figure SF28: Schematic representation of miRNA Target sites on NEFM
Figure SF29: Schematic representation of miRNA targets on NEFL
Figure SF30: Schematic representation of miRNA Target sites on NEFH
Figure SF31: Schematic representation of miRNA Target sites on MAPK14
Figure SF32: Schematic representation of miRNA Target sites on MAP3K5
Figure SF33: Schematic representation of miRNA Target sites on MAP2K6
Figure SF34: Schematic representation of miRNA Target sites on MAP2K3
Figure SF35: Schematic representation of miRNA Target sites on PRPH
Figure SF36: Schematic representation of miRNA conserved target on BCL2
Figure SF37: Schematic representation of miRNA conserved target on MAP2K3

## Figures and Tables

**Table 1:** Multiplicity and cooperativity for all miRNAs.

S. no.	Gene name	hsa-miR-370	hsa-miR-874	hsa-miR-423-3p	hsa-miR-323-5p	hsa-miR-760	hsa-miR-149	hsa-miR-139-3p	hsa-miR-744	hsa-miR-324-3p	hsa-miR-339-3p	hsa-miR-654-5p	Total
1	ALS2	1	1	1	1	1	2	—	1	1	—	—	9
2	APAF1	1	1	1	—	3	1	2	—	—	—	1	10
3	BAD	4	1	2	—	2	3	3	3	1	—	—	19
4	BAX	1	1	3	1	3	1	1	—	—	1	1	13
5	BCL2	1	1	—	1	1	1	2	1	—	1	2	11
6	BCL2L1	1	3	—	—	—	—	1	1	—	—	1	7
7	BID	2	1	1	2	—	1	—	3	1	—	—	11
8	CASP1	—	—	—	—	1	—	—	—	1	—	—	2
9	GRIA1	1	1	1	—	1	2	—	—	1	—	1	8
10	GPX1	—	3	2	2	1	—	—	1	—	2	—	11
11	DERL1	—	1	1	—	—	1	—	—	1	—	1	4
12	DAXX	1	2	—	—	2	—	—	—	1	—	3	9
13	CYCS	3	2	2	1	—	2	—	—	1	1	—	12
14	CHP	3	—	—	1	—	1	1	—	—	—	—	6
15	CCS	—	3	—	—	—	4	1	—	1	—	1	10
16	CAT	1	1	1	1	1	1	—	—	—	—	—	6
17	CASP9	1	3	2	2	1	—	—	—	1	1	—	11
18	CASP3	1	1	—	1	1	1	—	—	—	—	—	5
19	TOMM40	4	3	2	2	3	1	2	1	1	—	1	20
20	TNFRSF1A	5	2	4	4	1	1	1	—	2	1	1	22
21	TNF	1	2	—	—	2	—	1	—	—	1	—	7
22	SOD2	—	—	—	—	—	—	—	—	—	—	—	0
23	SLC1A2	3	1	3	1	1	1	—	2	1	2	1	16
24	RAC1	4	—	—	—	1	—	—	1	—	1	—	7
25	RAB5A	1	—	2	1	—	1	—	2	—	1	1	9
26	p53	1	1	1	1	1	—	2	2	—	1	1	11
27	NOS1	2	1	3	3	2	2	2	1	—	—	1	17
28	NEFM	3	2	1	2	2	1	2	1	1	—	1	16
29	NEFL	3	1	4	1	1	—	1	—	1	1	—	13
30	NEFH	3	1	2	2	2	—	—	—	1	2	1	14
31	MAPK14	4	3	3	4	1	1	—	—	—	—	—	16
32	MAP3K5	2	3	1	5	2	2	—	—	—	2	—	17
33	MAP2K6	2	—	—	—	1	—	—	1	—	—	—	4
34	MAP2K3	2	4	—	3	2	1	1	—	1	1	1	16
35	PRPH	3	2	2	2	1	4	2	1	3	2	1	23

Total		65	52	45	44	41	36	25	22	21	21	21	392

**Table 2:** Number of target sites predicted in 3′ UTR using miRanda, TargetScan, and PicTar.

Site of miRNA expression	Target sites predicted using miRanda	Target sites predicted using TargetScan	Target sites predicted using PicTar
miRNA expressed in midbrain	115	975	82
miRNA expressed in cerebellum	46	492	60
miRNA expressed in hippocampus	104	831	81
miRNA expressed in frontal cortex	88	872	82

**Table 3:** Common microRNA target sites for various genes predicted using miRanda and TargetScan.

Gene	MicroRNA	Start position of target sites predicted by miRanda	End position of target sites predicted by miRanda	Start position of target sites predicted by TargetScan	End position of target sites predicted by TargetScan
BAD	hsa-miR-744	211	236	228	234
BCL2	hsa-miR-195	2520	2537	2521	2527
BCL2	hsa-miR-139-5p	1211	1234	1218	1224
BCL2	hsa-miR-25	4228	4253	4237	4243
BCL2	hsa-miR-24	1007	1027	1011	1017
BCL2L1	hsa-miR-486-5p	657	678	670	676
CASP3	hsa-miR-370	1311	1333	1324	1330
CCS	hsa-miR-486-5p	89	111	103	109
CYCS	hsa-miR-93	3333	3357	3348	3354
CYCS	hsa-miR-93	4143	4167	4158	4164
CYCS	hsa-miR-125b	1564	1590	1582	1588
CYCS	hsa-miR-17	3333	3357	3348	3354
CYCS	hsa-miR-17	4143	4167	4158	4164
CYCS	hsa-miR-25	2189	2212	2204	2210
CYCS	hsa-miR-25	2597	2619	2610	2616
CYCS	hsa-miR-92a	2596	2619	2610	2616
CYCS	hsa-miR-93	4143	4167	4158	4164
CYCS	hsa-miR-20a	4143	4167	4158	4164
CYCS	hsa-miR-221	1576	1599	1592	1598
CYCS	hsa-miR-93	3333	3357	3348	3354
MAP2K3	hsa-miR-103	286	307	299	305
MAP2K3	hsa-miR-107	283	307	299	305
MAP2K3	hsa-miR-760	16	39	32	38
MAP2K3	hsa-miR-874	83	106	99	105
MAP2K3	hsa-miR-324-3p	40	64	56	62
p53	hsa-miR-30e	270	294	286	292
SLC1A2	hsa-miR-628-5p	2533	2551	2541	2547
TNF	hsa-miR-185	353	382	375	381

**Table 4:** Summary of intersection of results.

Part of nervous system	Target sites common in miRanda and TargetScan	Target sites common in miRanda and PicTar	Target sites common in TargetScan and PicTar	Target sites conserved in all 3 software
Midbrain	14	3	74	3
Cerebellum	4	2	55	2
Hippocampus	16	3	42	3
Frontal cortex	14	3	73	3
