# Supplementary material for: A Complex Network of MicroRNAs Expressed in Brain and Genes Associated with Amyotrophic Lateral Sclerosis
Source: Int J Genomics. 2013 Jul 10;2013:383024. doi: 10.1155/2013/383024 (PMC3723150; doi:10.1155/2013/383024)
Supplement: Supplementary file 1 — Table S1: Information of Amyotrophic Lateral Sclerosis Disease associated genes selected from KEGG pathway database. Table S2: Distribution of target sites predicted across gene in 5' UTR, CDS and 3' UTR for miRNAs expressed in midbrain, cerebellum, frontal cortex and hippocampus predicted by miRanda. Table S3: Target sites predicted for miRNAs expressed in midbrain, cerebellum, hippocampus and frontal cortex using TargetScan. Table S4: Target sites predicted for miRNAs expressed in midbrain, cerebellum, hippocampus and frontal cortex using Pictar. Table S5: Comparison of target site prediction results obtained using TargetScan and Pictar. Table S6: Comparison of target site prediction results obtained using miRanda and Pictar. Table S7: Hot spots identified in selected genes in 5'UTR , CDS and 3' UTR for miRNAs considered in the study. Figure SF1: Schematic representation of miRNA Target sites on ALS2 Figure SF2: Schematic representation of miRNA Target sites on APAF1 Figure SF3: Schematic representation of miRNA Target sites on BAD Figure SF4: Schematic representation of miRNA Target sites on BAX Figure SF 5: Schematic representation of miRNA Target sites on BCL2 Figure SF 6: Schematic representation of miRNA Target sites on BCL2L1 Figure SF7: Schematic representation of miRNA Target sites on BID Figure SF8: Schematic representation of miRNA Target sites on CASP1 Figure SF8: Schematic representation of miRNA Target sites on CASP1 Figure SF9: Schematic representation of miRNA targets on GRIA1 Figure SF10: Schematic representation of miRNA Target sites on GPX1 Figure SF11: Schematic representation of miRNA Target sites on DERL1 Figure SF12: Schematic representation of miRNA Target sites on DAXX Figure SF13: Schematic representation of miRNA Target sites on CYCS Figure SF14. Schematic representation of miRNA targets on CHP Figure SF15: Schematic representation of miRNA Target sites on CCS Figure SF16: Schematic representation of miRNA Target sites on CAT Figure SF17: [file 383024.f1.zip › f1.383024/S1 Supplementary Table Gene Information.pdf]

S. NO	GENE SYMBOL	SYNONYMS	ACCESSION NUMBER	GENE ID	GENE LENGTH (bp)	CDS POSITION	GENE LOCATION	FUNCTION OF GENES*
1.	**SLC1A2 solute carrier family 1 (glial high affinity glutamate transporter), member 2**	EAAT2, GLT-1, SLC1A2	NM_004171	6506	12021	593-2317	11p13-p12	This gene encodes a member of a family of solute transporter proteins. The membrane-bound protein is the principal transporter that clears the excitatory neurotransmitter glutamate from the extracellular space at synapses in the central nervous system. Glutamate clearance is necessary for proper synaptic activation and to prevent neuronal damage from excessive activation of glutamate receptors. Mutations in and decreased expression of this protein are associated with amyotrophic lateral sclerosis. Alternatively spliced transcript variants of this gene have been described, but their full-length nature is not known.
2.	GRIA1 glutamate receptor, ionotropic, AMPA 1	GLUH1, GLUR1, GLURA, HBGR1, MGC133252, GRIA1	NM_000827	2890	3242	144-2864	5q33, 5q31.1	Glutamate receptors are the predominant excitatory neurotransmitter receptors in the mammalian brain and are activated in a variety of normal neurophysiologic processes. These receptors are heteromeric protein complexes with multiple subunits, each possessing transmembrane regions, and all arranged to form a ligand-gated ion channel. The classification of glutamate receptors is based on their activation by different pharmacologic agonists. This gene belongs to a family of alpha-amino-3-hydroxy-5-methyl-4-isoxazole propionate (AMPA) receptors. Alternatively spliced transcript variants encoding different isoforms have been found for this gene.
3.	TNF tumor necrosis factor (TNF superfamily, member 2)	DIF, TNFA, TNFSF2, TNF-alpha, TNF	NM_000594	7124	1669	170-871	6p21.3	This gene encodes a multifunctional proinflammatory cytokine that belongs to the tumor necrosis factor (TNF) superfamily. This cytokine is mainly secreted by macrophages. It can bind to, and thus functions through its receptors TNFRSF1A/TNFR1 and TNFRSF1B/TNFBR. This cytokine is involved in the regulation of a wide spectrum of biological processes including cell proliferation, differentiation, apoptosis, lipid metabolism, and coagulation. This

								cytokine has been implicated in a variety of diseases, including autoimmune diseases, insulin resistance, and cancer. Knockout studies in mice also suggested the neuroprotective function of this cytokine.
4.	TNFRSF1A tumor necrosis factor receptor superfamily, member 1A	FPF, p55, p60, TBP1, TNF-R, TNFAR, TNFR1, p55-R, CD120a, TNFR55, TNFR60, TNF-R-I, TNF-R55, MGC19588, TNFRSF1A	NM_001065	7132	2236	282-1649	12p13.2	The protein encoded by this gene is a member of the TNF-receptor superfamily. This protein is one of the major receptors for the tumor necrosis factor-alpha. This receptor can activate NF-kappaB, mediate apoptosis, and function as a regulator of inflammation. Antiapoptotic protein BCL2-associated athanogene 4 (BAG4/SODD) and adaptor proteins TRADD and TRAF2 have been shown to interact with this receptor, and thus play regulatory roles in the signal transduction mediated by the receptor. Germline mutations of the extracellular domains of this receptor were found to be associated with the autosomal dominant periodic fever syndrome. The impaired receptor clearance is thought to be a mechanism of the disease.
5.	CASP1 caspase 1, apoptosis-related cysteine peptidase (interleukin 1, beta, convertase)	ICE, P45, IL1BC, CASP1	NM_001223	834	1301	18-1169	11q23	This gene encodes a protein which is a member of the cysteine-aspartic acid protease (caspase) family. Sequential activation of caspases plays a central role in the execution-phase of cell apoptosis. Caspases exist as inactive proenzymes which undergo proteolytic processing at conserved aspartic residues to produce 2 subunits, large and small, that dimerize to form the active enzyme. This gene was identified by its ability to proteolytically cleave and activate the inactive precursor of interleukin-1, a cytokine involved in the processes such as inflammation, septic shock, and wound healing. This gene has been shown to induce cell apoptosis and may function in various developmental stages. Studies of a similar

								gene in mouse suggest a role in the pathogenesis of Huntington disease. Alternative splicing of this gene results in five transcript variants encoding distinct isoforms
6.	SOD1 superoxide dismutase 1, soluble	ALS, SOD, ALS1, IPOA, homodimer, SOD1	NM_000454	6647	981	149-613	21q22.1, 21q22.11	The protein encoded by this gene binds copper and zinc ions and is one of two isozymes responsible for destroying free superoxide radicals in the body. The encoded isozyme is a soluble cytoplasmic protein, acting as a homodimer to convert naturally-occuring but harmful superoxide radicals to molecular oxygen and hydrogen peroxide. The other isozyme is a mitochondrial protein. Mutations in this gene have been implicated as causes of familial amyotrophic lateral sclerosis. Rare transcript variants have been reported for this gene.
7.	TP53 tumor protein p53	p53, LFS1, TRP53, FLJ92943, TP53	NM_000546	7157	2640	252-1433	17p13.1	This gene encodes tumor protein p53, which responds to diverse cellular stresses to regulate target genes that induce cell cycle arrest, apoptosis, senescence, DNA repair, or changes in metabolism. p53 protein is expressed at low level in normal cells and at a high level in a variety of transformed cell lines, where it's believed to contribute to transformation and malignancy. p53 is a DNA-binding protein containing transcription activation, DNA-binding, and oligomerization domains. It is postulated to bind to a p53-binding site and activate expression of downstream genes that inhibit growth and/or invasion, and thus function as a tumor suppressor. Mutants of p53 that frequently occur in a number of different human cancers fail to bind the consensus DNA binding site, and hence cause the loss of tumor suppressor activity. Alterations of this gene occur not only as somatic mutations in human malignancies, but also as germline mutations in some cancer-prone families with Li-Fraumeni syndrome. Multiple p53 variants due to alternative promoters and multiple alternative splicing have been found. These variants encode distinct isoforms, which can regulate p53 transcriptional activity.

8.	CHP calcium binding protein P22	CHP, SLC9A1 BP	NM_007236	11261	3230	145-732	15q13.3	This gene encodes a phosphoprotein that binds to the Na+/H+ exchanger NHE1. This protein serves as an essential cofactor which supports the physiological activity of NHE family members and may play a role in the mitogenic regulation of NHE1. The protein shares similarity with calcineurin B and calmodulin and it is also known to be an endogenous inhibitor of calcineurin activity.
9.	BID BH3 interacting domain death agonist	FP497, MGC153 19, MGC423 55, BID	NM_197967	637	2144	389-688	22q11.1	This gene encodes a death agonist that heterodimerizes with either agonist BAX or antagonist BCL2. The encoded protein is a member of the BCL-2 family of cell death regulators. It is a mediator of mitochondrial damage induced by caspase-8 (CASP8), CASP8 cleaves this encoded protein, and the COOH-terminal part translocates to mitochondria where it triggers cytochrome c release. Multiple alternatively spliced transcript variants have been found, but the full-length nature of some variants has not been defined.
10.	TOMM40 translocase of outer mitochondrial membrane 40 homolog (yeast)	TOM40, PEREC1, C19orf1, PER-EC1, D19S117 7E, TOMM4 0	NM_006114	10452	1700	85-1170	19q13	TOMM40 is the channel-forming subunit of the translocase of the mitochondrial outer membrane (TOM) complex that is essential for protein import into mitochondria
11.	BCL2 B-cell CLL/lymphom a 2<br>(Bcl-2, BCL2)		NM_000633	596	6492	494-1213	18q21.33, 18q21.3	This gene encodes an integral outer mitochondrial membrane protein that blocks the apoptotic death of some cells such as lymphocytes. Constitutive expression of BCL2, such as in the case of translocation of BCL2 to Ig heavy chain locus, is thought to be the cause of follicular lymphoma. Two transcript variants, produced by alternate splicing, differ in their C-terminal ends.

12.	BCL2L1 BCL2-like 1	BCLX, BCL2L, Bcl-X, bcl-xL, bcl-xS, BCL-XL/S, DKFZp781P2092, BCL2L1	NM_138578	598	2575	367-1068	20q11.21	The protein encoded by this gene belongs to the BCL-2 protein family. BCL-2 family members form hetero- or homodimers and act as anti- or pro-apoptotic regulators that are involved in a wide variety of cellular activities. The proteins encoded by this gene are located at the outer mitochondrial membrane, and have been shown to regulate outer mitochondrial membrane channel (VDAC) opening. VDAC regulates mitochondrial membrane potential, and thus controls the production of reactive oxygen species and release of cytochrome C by mitochondria, both of which are the potent inducers of cell apoptosis. Two alternatively spliced transcript variants, which encode distinct isoforms, have been reported. The longer isoform acts as an apoptotic inhibitor and the shorter form acts as an apoptotic activator.
13.	BAX BCL2-associated X protein	BCL2L4, BAX	NM_138764	581	986	70-564	19q13.3-q13.4	The protein encoded by this gene belongs to the BCL2 protein family. BCL2 family members form hetero- or homodimers and act as anti- or pro-apoptotic regulators that are involved in a wide variety of cellular activities. This protein forms a heterodimer with BCL2, and functions as an apoptotic activator. This protein is reported to interact with, and increase the opening of, the mitochondrial voltage-dependent anion channel (VDAC), which leads to the loss in membrane potential and the release of cytochrome c. The expression of this gene is regulated by the tumor suppressor P53 and has been shown to be involved in P53-mediated apoptosis. Multiple alternatively spliced transcript variants, which encode different isoforms, have been reported for this gene.
14.	BAD BCL2-associated agonist of cell death	BBC2, BCL2L8, BAD	NM_032989	572	956	71-577	11q13.1	The protein encoded by this gene is a member of the BCL-2 family. BCL-2 family members are known to be regulators of programmed cell death. This protein positively regulates cell apoptosis by forming heterodimers with BCL-xL and BCL-2, and reversing their death repressor activity. Proapoptotic activity of this protein is regulated through its phosphorylation. Protein kinases AKT and

								MAP kinase, as well as protein phosphatase calcineurin were found to be involved in the regulation of this protein. Alternative splicing of this gene results in two transcript variants which encode the same isoform.
15.	CYCS cytochrome c, somatic	CYC, HCS, THC4, CYCS	NM_018947	54205	5518	146-463	7p15.2	This gene encodes cytochrome c, a component of the electron transport chain in mitochondria. The heme group of cytochrome c accepts electrons from the b-c1 complex and transfers electrons to the cytochrome oxidase complex. Cytochrome c is also involved in initiation of apoptosis. Upon release of cytochrome c to the cytoplasm, the protein binds apoptotic protease activating factor which activates the apoptotic initiator procaspase 9. Many cytochrome c pseudogenes exist, scattered throughout the human genome.
16.	APAF1 apoptotic peptidase activating factor 1	CED4, APAF-1, DKFZp781B1145, APAF1	NM_181861	317	7204	578-4324	12q23	This gene encodes a cytoplasmic protein that initiates apoptosis. This protein contains several copies of the WD-40 domain, a caspase recruitment domain (CARD), and an ATPase domain (NB-ARC). Upon binding cytochrome c and dATP, this protein forms an oligomeric apoptosome. The apoptosome binds and cleaves caspase 9 preproprotein, releasing its mature, activated form. Activated caspase 9 stimulates the subsequent caspase cascade that commits the cell to apoptosis. Alternative splicing results in several transcript variants encoding different isoforms.
17.	CASP9 caspase 9, apoptosis-related cysteine peptidase	MCH6, APAF3, APAF-3, ICE-LAP6, CASPASE-9c, CASP9	NM_032996	842	1584	96-896	1p36.3-p36.1	This gene encodes a member of the cysteine-aspartic acid protease (caspase) family. Sequential activation of caspases plays a central role in the execution-phase of cell apoptosis. Caspases exist as inactive proenzymes which undergo proteolytic processing at conserved aspartic residues to produce two subunits, large and small, that dimerize to form the active enzyme. This protein is processed by caspase APAF1, this step is thought to be one of the earliest in the caspase activation cascade. Alternative splicing

								results in two transcript variants which encode different isoforms.
18.	CASP3 caspase 3, apoptosis-related cysteine peptidase	CPP32, SCA-1, CPP32B, CASP3	NM_032991	836	2522	97-930	4q34	This gene encodes a protein which is a member of the cysteine-aspartic acid protease (caspase) family. Sequential activation of caspases plays a central role in the execution-phase of cell apoptosis. Caspases exist as inactive proenzymes which undergo proteolytic processing at conserved aspartic residues to produce two subunits, large and small, that dimerize to form the active enzyme. This protein cleaves and activates caspases 6, 7 and 9, and the protein itself is processed by caspases 8, 9 and 10. It is the predominant caspase involved in the cleavage of amyloid-beta 4A precursor protein, which is associated with neuronal death in Alzheimer's disease. Alternative splicing of this gene results in two transcript variants that encode the same protein.
19.	DERL1 Der1-like domain family, member 1	DER1, DER-1, MGC3067, PRO2577, FLJ13784, FLJ42092, DERL1	NM_024295	79139	3033	180-935	8q24.13	
20.	MAP3K5 mitogen-activated protein kinase kinase kinase 5	ASK1, MEKK5, MAPKKK5, MAP3K5	NM_005923	4217	5215	362-4486	6q22.33	Mitogen-activated protein kinase (MAPK) signaling cascades include MAPK or extracellular signal-regulated kinase (ERK), MAPK kinase (MKK or MEK), and MAPK kinase kinase (MAPKKK or MEKK). MAPKK kinase/MEKK phosphorylates and activates its downstream protein kinase, MAPK kinase/MEK, which in turn activates MAPK. The kinases of these signaling cascades are highly conserved, and homologs exist in yeast,

								Drosophila, and mammalian cells. MAPKKK5 contains 1,374 amino acids with all 11 kinase subdomains. Northern blot analysis shows that MAPKKK5 transcript is abundantly expressed in human heart and pancreas. The MAPKKK5 protein phosphorylates and activates MKK4 (aliases SERK1, MAPKK4) in vitro, and activates c-Jun N-terminal kinase (JNK)/stress-activated protein kinase (SAPK) during transient expression in COS and 293 cells, MAPKKK5 does not activate MAPK/ERK.
21.	DAXX death-domain associated protein	DAP6, EAP1, BING2, MGC126245, MGC126246, DAXX	NM_001350	1616	2477	116-2338	6p21.3	This gene encodes a multifunctional protein that resides in multiple locations in the nucleus and in the cytoplasm. It interacts with a wide variety of proteins, such as apoptosis antigen Fas, centromere protein C, and transcription factor erythroblastosis virus E26 oncogene homolog 1. In the nucleus, the encoded protein functions as a potent transcription repressor that binds to sumoylated transcription factors. Its repression can be relieved by the sequestration of this protein into promyelocytic leukemia nuclear bodies or nucleoli. This protein also associates with centromeres in G2 phase. In the cytoplasm, the encoded protein may function to regulate apoptosis. The subcellular localization and function of this protein are modulated by post-translational modifications, including sumoylation, phosphorylation and polyubiquitination. Alternative splicing results in multiple transcript variants.
22.	MAP2K3 mitogen-activated protein kinase kinase 3	MEK3, MKK3, MAPKK3, PRKMK3, MAP2K3	NM_145109	5606	2319	266-1309	17q11.2	The protein encoded by this gene is a dual specificity protein kinase that belongs to the MAP kinase kinase family. This kinase is activated by mitogenic and environmental stress, and participates in the MAP kinase-mediated signaling cascade. It phosphorylates and thus activates MAPK14/p38-MAPK. This kinase can be activated by insulin, and is necessary for the expression of glucose transporter. Expression of RAS oncogene is found to result in the accumulation of the active form of this kinase, which thus leads to the constitutive activation of MAPK14, and confers oncogenic

								transformation of primary cells. The inhibition of this kinase is involved in the pathogenesis of Yersina pseudotuberculosis. Multiple alternatively spliced transcript variants that encode distinct isoforms have been reported for this gene.
23.	MAP2K6 mitogen-activated protein kinase kinase 6	MEK6, MKK6, MAPKK 6, PRKMK 6, SAPKK3 , MAP2K 6	NM_002758	5608	1879	289-1293	17q24.3	This gene encodes a member of the dual specificity protein kinase family, which functions as a mitogen-activated protein (MAP) kinase kinase. MAP kinases, also known as extracellular signal-regulated kinases (ERKs), act as an integration point for multiple biochemical signals. This protein phosphorylates and activates p38 MAP kinase in response to inflammatory cytokines or environmental stress. As an essential component of p38 MAP kinase mediated signal transduction pathway, this gene is involved in many cellular processes such as stress induced cell cycle arrest, transcription activation and apoptosis.
24.	MAPK14 mitogen-activated protein kinase 14	RK, p38, EXIP, Mxi2, CSBP1, CSBP2, CSPB1, PRKM14 , PRKM15 , SAPK2A , p38ALP HA, MAPK1 4	NM_139012	1432	3757	363-1445	6p21.3-p21.2	The protein encoded by this gene is a member of the MAP kinase family. MAP kinases act as an integration point for multiple biochemical signals, and are involved in a wide variety of cellular processes such as proliferation, differentiation, transcription regulation and development. This kinase is activated by various environmental stresses and proinflammatory cytokines. The activation requires its phosphorylation by MAP kinase kinases (MKKs), or its autophosphorylation triggered by the interaction of MAP3K7IP1/TAB1 protein with this kinase. The substrates of this kinase include transcription regulator ATF2, MEF2C, and MAX, cell cycle regulator CDC25B, and tumor suppressor p53, which suggest the roles of this kinase in stress related transcription and cell cycle regulation, as well as in genotoxic stress response. Four alternatively spliced transcript variants of this gene encoding distinct isoforms have been reported.
25.	NOS1 nitric oxide synthase	NOS, nNOS,	NM_000620	4842	7124	686-4990	12q24.2-	Nitric oxide (NO) is a messenger molecule with diverse functions throughout the body. In the brain and peripheral nervous system,

	1 (neuronal	IHPS1, NOS1					q24.31	NO displays many properties of a neurotransmitter, it is implicated in neurotoxicity associated with stroke and neurodegenerative diseases, neural regulation of smooth muscle, including peristalsis, and penile erection. NO is also responsible for endothelium-derived relaxing factor activity regulating blood pressure. In macrophages, NO mediates tumoricidal and bactericidal actions, as indicated by the fact that inhibitors of NO synthase (NOS) block these effects. Neuronal NOS and macrophage NOS (MIM 163730) are distinct isoforms (Lowenstein et al., 1992 [PubMed 1379716]). Both the neuronal and the macrophage forms are unusual among oxidative enzymes in requiring several electron donors: FAD (see MIM 610595), flavin mononucleotide (FMN), NADPH, and tetrahydrobiopterin
26.	CCS copper chaperone for superoxide dismutase	MGC138260, CCS	NM_005125	9973	1068	44-868	11q13	Copper chaperone for superoxide dismutase specifically delivers Cu to copper/zinc superoxide dismutase and may activate copper/zinc superoxide dismutase through direct insertion of the Cu cofactor.
27.	GPX1 glutathione peroxidase 1	GSHPX1, MGC14399, MGC88245, GPX1	NM_000581	2876	921	**81-692**	3p21.3	This gene encodes a member of the glutathione peroxidase family. Glutathione peroxidase functions in the detoxification of hydrogen peroxide, and is one of the most important antioxidant enzymes in humans. This protein is one of only a few proteins known in higher vertebrates to contain selenocysteine, which occurs at the active site of glutathione peroxidase and is coded by UGA, that normally functions as a translation termination codon. In addition, this protein is characterized in a polyalanine sequence polymorphism in the N-terminal region, which includes three alleles with five, six or seven alanine (ALA) repeats in this sequence. The allele with five ALA repeats is significantly associated with breast cancer risk. Two alternatively spliced transcript variants encoding distinct isoforms

								have been found for this gene.
28.	CAT catalase	MGC138422, MGC138424, CAT	NM_001752	847	2305	**84-1667**	11p13	
**29.**	**NEFL neurofilament, light polypeptide**	NFL, NF-L, NF68, CMT1F, CMT2E, FLJ53642, NEFL	NM_006158	4747	3584	**98-1729**	8p21	Neurofilaments are type IV intermediate filament heteropolymers composed of light, medium, and heavy chains. Neurofilaments comprise the axoskeleton and they functionally maintain the neuronal caliber. They may also play a role in intracellular transport to axons and dendrites. This gene encodes the light chain neurofilament protein. Mutations in this gene cause Charcot-Marie-Tooth disease types 1F (CMT1F) and 2E (CMT2E), disorders of the peripheral nervous system that are characterized by distinct neuropathies. A pseudogene has been identified on chromosome Y.
**30.**	**NEFM neurofilament, medium polypeptide**	NFM, NEF3, NF-M, NEFM	NM_005382	4741	3271	**34-2784**	8p21	Neurofilaments are type IV intermediate filament heteropolymers composed of light, medium, and heavy chains. Neurofilaments comprise the axoskeleton and functionally maintain neuronal caliber. They may also play a role in intracellular transport to axons and dendrites. This gene encodes the medium neurofilament protein. This protein is commonly used as a biomarker of neuronal damage. Alternative splicing results in multiple transcript variants encoding distinct isoforms.
**31.**	**NEFH neurofilament, heavy polypeptide**	NFH, NEFH	NM_021076	4744	3695	**34-3096**	22q12.2	Neurofilaments are type IV intermediate filament heteropolymers composed of light, medium, and heavy chains. Neurofilaments comprise the axoskeleton and functionally maintain neuronal caliber. They may also play a role in intracellular transport to axons and dendrites. This gene encodes the heavy neurofilament protein. This protein is commonly used as a biomarker of neuronal damage and susceptibility to amyotrophic lateral sclerosis (ALS) has been associated with mutations in this gene.

**32.**	**PRPH peripherin**	NEF4, PRPH1, PRPH	NM_006262	5630	1833	**76-1488**	12q12-q13	This gene encodes a cytoskeletal protein found in neurons of the peripheral nervous system. The encoded protein is a type III intermediate filament protein with homology to other cytoskeletal proteins such as desmin, and is a different protein that the peripherin found in photoreceptors. Mutations in this gene have been associated with susceptibility to amyotrophic lateral sclerosis.
**33.**	**ALS2 amyotrophic lateral sclerosis 2 (juvenile)**	ALSJ, PLSJ, IAHSP, ALS2CR6, FLJ31851, KIAA1563, MGC87187, ALS2	NM_020919	57679	6470	**200-5173**	2q33.1	The protein encoded by this gene contains an ATS1/RCC1-like domain, a RhoGEF domain, and a vacuolar protein sorting 9 (VPS9) domain, all of which are guanine-nucleotide exchange factors that activate members of the Ras superfamily of GTPases. The protein functions as a guanine nucleotide exchange factor for the small GTPase RAB5. The protein localizes with RAB5 on early endosomal compartments, and functions as a modulator for endosomal dynamics. Mutations in this gene result in several forms of juvenile lateral sclerosis and infantile-onset ascending spastic paralysis. Multiple transcript variants encoding different isoforms have been found for this gene.
**34.**	**RAB5A RAB5A, member RAS oncogene family**	RAB5, RAB5A	NM_004162	5868	2352	**377-1024**	3p24-p22	
**35.**	**RAC1 ras-related C3 botulinum toxin substrate 1 (rho family, small GTP**	MIG5, TC-25, p21-Rac1, MGC111543, RAC1	NM_198829	5879	2413	**432-878**	7p22	The protein encoded by this gene is a GTPase which belongs to the RAS superfamily of small GTP-binding proteins. Members of this superfamily appear to regulate a diverse array of cellular events, including the control of cell growth, cytoskeletal reorganization, and the activation of protein kinases. Two transcript variants encoding different isoforms have been found for this gene.

	**binding protein Rac1)**							

Table S1: Information of Amyotrophic Lateral Sclerosis Disease associated genes selected from KEGG pathway database
