# Supplementary material for: A Complex Network of MicroRNAs Expressed in Brain and Genes Associated with Amyotrophic Lateral Sclerosis
Source: Int J Genomics. 2013 Jul 10;2013:383024. doi: 10.1155/2013/383024 (PMC3723150; doi:10.1155/2013/383024)
Supplement: Supplementary file 1 — Table S1: Information of Amyotrophic Lateral Sclerosis Disease associated genes selected from KEGG pathway database. Table S2: Distribution of target sites predicted across gene in 5' UTR, CDS and 3' UTR for miRNAs expressed in midbrain, cerebellum, frontal cortex and hippocampus predicted by miRanda. Table S3: Target sites predicted for miRNAs expressed in midbrain, cerebellum, hippocampus and frontal cortex using TargetScan. Table S4: Target sites predicted for miRNAs expressed in midbrain, cerebellum, hippocampus and frontal cortex using Pictar. Table S5: Comparison of target site prediction results obtained using TargetScan and Pictar. Table S6: Comparison of target site prediction results obtained using miRanda and Pictar. Table S7: Hot spots identified in selected genes in 5'UTR , CDS and 3' UTR for miRNAs considered in the study. Figure SF1: Schematic representation of miRNA Target sites on ALS2 Figure SF2: Schematic representation of miRNA Target sites on APAF1 Figure SF3: Schematic representation of miRNA Target sites on BAD Figure SF4: Schematic representation of miRNA Target sites on BAX Figure SF 5: Schematic representation of miRNA Target sites on BCL2 Figure SF 6: Schematic representation of miRNA Target sites on BCL2L1 Figure SF7: Schematic representation of miRNA Target sites on BID Figure SF8: Schematic representation of miRNA Target sites on CASP1 Figure SF8: Schematic representation of miRNA Target sites on CASP1 Figure SF9: Schematic representation of miRNA targets on GRIA1 Figure SF10: Schematic representation of miRNA Target sites on GPX1 Figure SF11: Schematic representation of miRNA Target sites on DERL1 Figure SF12: Schematic representation of miRNA Target sites on DAXX Figure SF13: Schematic representation of miRNA Target sites on CYCS Figure SF14. Schematic representation of miRNA targets on CHP Figure SF15: Schematic representation of miRNA Target sites on CCS Figure SF16: Schematic representation of miRNA Target sites on CAT Figure SF17: [file 383024.f1.zip › f1.383024/S2-7 Supplementary Tables.pdf]

Table S2 : Distribution of target sites predicted across gene in 5' UTR, CDS and 3' UTR for miRNAs expressed in midbrain, cerebellum, frontal cortex and hippocampus predicted by miRanda.

S.No.	Gene Name	miRNAs expressed in midbrain			miRNAs expressed in Cerebellum			miRNAs expressed in Hippocampus			miRNAs expressed in Frontal Cortex		
		5'	CDS	3'	5'	CDS	3'	5'	CDS	3'	5'	CDS	3'
1	*ALS2*	2	17	1	2	3	0	3	11	0	2	14	1
2	*APAF1*	7	4	12	1	1	4	3	5	11	3	4	6
3	*BAD*	2	12	4	1	3	1	1	7	2	2	12	3
4	*BAX*	4	9	1	2	2	1	2	3	3	3	5	2
5	*BCL2*	1	3	15	0	1	4	0	3	11	1	3	11
6	*BCL2L1*	2	8	5	1	1	3	2	3	6	5	1	3
7	*BID*	4	4	3	2	3	0	4	4	1	7	4	1
8	*CASP1*	0	3	0	0	2	0	0	3	0	0	2	0
9	*GRIA1*	0	12	0	0	2	0	0	3	0	0	7	0
10	*GPX1*	0	5	0	4	0	1	1	7	2	0	2	1
11	*DERL1*	5	1	2	1	1	1	2	3	1	2	2	2
12	*DAXX*	0	11	0	0	4	0	0	8	0	0	7	0
13	*CYCS*	2	0	13	0	0	6	2	0	10	3	0	11
14	*CHP*	2	3	7	1	0	5	3	3	9	3	1	7
15	*CCS*	1	15	1	0	7	0	0	8	1	1	15	0
16	*CAT*	3	2	0	0	2	0	0	5	0	1	5	0
17	*CASP9*	3	6	4	1	3	0	1	7	3	2	4	2
18	*CASP3*	2	1	6	0	0	2	1	1	6	1	0	4
19	*TOMM40*	1	12	4	2	4	0	3	11	1	2	12	1
20	*TNFRSF1A*	3	13	5	2	7	1	2	13	3	3	17	3
21	*TNF*	0	8	1	0	4	0	1	9	1	5	0	3
22	*SOD2*	2	2	0	2	0	0	2	2	0	2	0	0
23	*SLC1A2*	5	6	7	5	2	1	9	4	7	7	4	4
24	*RAC1*	0	2	1	0	1	0	0	2	2	3	3	2
25	*RAB5A*	8	0	1	2	0	1	4	0	0	5	0	1
26	*p53*	4	9	5	3	4	2	4	9	2	3	6	4
27	*NOS1*	8	17	4	3	7	1	4	15	3	4	13	5
28	*NEFM*	0	14	0	0	4	0	0	12	0	0	12	0
29	*NEFL*	0	10	0	1	1	0	1	5	2	1	7	0
30	*NEFH*	0	15	0	0	1	1	2	9	1	0	8	0
31	*MAPK14*	8	5	3	6	2	1	11	5	5	8	6	3

32	*MAP3K5*	2	20	2	2	6	2	9	24	0	2	18	0
33	*MAP2K6*	1	5	1	1	1	1	1	2	1	1	5	1
34	*MAP2K3*	1	10	6	0	1	6	4	6	9	2	7	6
35	*PRPH*	2	13	1	1	3	1	1	10	1	1	16	1

Table S3 : Target sites predicted for miRNAs expressed in midbrain, cerebellum, hippocampus and frontal cortex using TargetScan

S.No.	Gene Name	miRNAs expressed in midbrain	miRNAs expressed in Cerebellum	miRNAs expressed in Hippocampus	miRNAs expressed in Frontal Cortex
		3'	3'	3'	3'
1	*ALS2*	26	17	21	25
2	*APAF1*	66	33	46	55
3	*BAD*	4	2	4	6
4	*BAX*	3	2	3	4
5	*BCL2*	92	47	70	74
6	*BCL2L1*	23	18	22	25
7	*BID*	24	12	23	22
8	*CASP1*	3	18	23	5
9	*GRIA1*	38	12	31	33
10	*GPX1*		15	4	3
11	*DERL1*	39	1	32	33
12	*DAXX*	1	16	1	1
13	*CYCS*	88	49	79	76
14	*CHP*	37	16	32	34
15	*CCS*	4	3	5	3
16	*CAT*	8	3	5	9
17	*CASP9*	5	5	10	9
18	*CASP3*	50	30	6	52
19	*TOMM40*	7	6	45	9
20	*TNFRSF1A*	8	5	7	9
21	*TNF*	16	9	8	15
22	*SOD2*	4	1	5	5

23	*SLC1A2*	167	68	138	132
24	*RAC1*	18	9	20	20
25	*RAB5A*	26	14	18	17
26	*p53*	33	20	33	30
27	*NOS1*	25	15	22	25
28	*NEFM*	23	18	20	19
29	*NEFL*	31	12	28	28
30	*NEFH*	17	7	16	17
31	*MAPK14*	32	14	27	23
32	*MAP3K5*	18	10	12	18
33	*MAP2K6*	15	6	11	10
34	*MAP2K3*	19	9	16	22
35	*PRPH*	3	1	3	4

Table S4 : Target sites predicted for miRNAs expressed in midbrain, cerebellum, hippocampus and frontal cortex using Pictar.

S.No.	Gene Name	miRNAs expressed in midbrain	miRNAs expressed in Cerebellum	miRNAs expressed in Hippocampus	miRNAs expressed in Frontal Cortex
		3'	3'	3'	3'
1	*ALS2*	5	4	6	6
2	*APAF1*	2	1	2	2
3	*BAD*	-	-	-	-
4	*BAX*	-	-	-	-
5	*BCL2*	8	6	8	8
6	*BCL2L1*	8	8	7	8
7	*BID*	-	-	-	-
8	*CASP1*	-	-	-	-
9	*GRIA1*	-	-	-	-
10	*GPX1*	-	-	-	-
11	*DERL1*	3	3	3	3
12	*DAXX*	-	-	-	-
13	*CYCS*	-	-	-	-

14	*CHP*	1	1		
15	*CCS*	-	-	-	-
16	*CAT*	-	-	-	-
17	*CASP9*	-	-	-	-
18	*CASP3*	9	8	9	8
19	*TOMM40*	-	-	-	-
20	*TNFRSF1A*	3	3	3	3
21	*TNF*	-	-	-	-
22	*SOD2*	-	-	1	1
23	*SLC1A2*	9	3	10	12
24	*RAC1*	3	2	3	3
25	*RAB5A*	3	3	2	2
26	*p53*	-	-	-	-
27	*NOS1*	-	-	-	-
28	*NEFM*	6	4	5	4
29	*NEFL*	-	-	-	-
30	*NEFH*	3	1	2	2
31	*MAPK14*	6	3	5	5
32	*MAP3K5*	4	2	5	5
33	*MAP2K6*	3	3	4	3
34	*MAP2K3*	6	5	7	7
35	*PRPH*	-	-	-	-

Table S5: Comparison of target site prediction results obtained using TargetScan and Pictar.

miRNA	TargetScan		PicTar	
	Start position of target site	End position of target site	Start position of target site	Gene name
hsa-miR-142-5p	1203	1209	1204	*ALS2*
hsa-miR-26a	447	453	449	*ALS2*
hsa-miR-26b	447	453	449	*ALS2*
hsa-miR-27a	2151	2157	2147	*APAF1*
hsa-miR-27b	2151	2157	2147	*APAF1*
hsa-miR-143	1811	1817	1819	*BCL2*

hsa-miR-143	4513	4519	4521	*BCL2*
hsa-miR-153	25	31	26	*BCL2*
hsa-miR-15a	2521	2527	2530	*BCL2*
hsa-miR-16	2521	2527	2530	*BCL2*
hsa-miR-195	2521	2527	2530	*BCL2*
hsa-miR-204	208	214	210	*BCL2*
hsa-miR-21	712	718	720	*BCL2*
hsa-let-7a	944	950	946	*BCL2L1*
hsa-let-7b	944	950	946	*BCL2L1*
hsa-let-7c	944	950	946	*BCL2L1*
hsa-let-7d	944	950	945	*BCL2L1*
hsa-let-7e	944	950	946	*BCL2L1*
hsa-let-7f	944	950	946	*BCL2L1*
hsa-let-7g	944	950	946	*BCL2L1*
hsa-let-7i	944	950	946	*BCL2L1*
hsa-miR-142-3p	87	93	88	*BCL2L1*
hsa-miR-214	975	981	976	*BCL2L1*
hsa-let-7a	152	158	154	*CASP3*
hsa-let-7b	152	158	154	*CASP3*
hsa-let-7c	152	158	154	*CASP3*
hsa-let-7e	152	158	154	*CASP3*
hsa-let-7f	152	158	154	*CASP3*
hsa-let-7g	152	158	154	*CASP3*
hsa-let-7i	152	158	154	*CASP3*
hsa-miR-30b	1187	1193	1188	*CASP3*
hsa-miR-30d	1187	1193	1188	*CASP3*
hsa-miR-30e	1187	1193	1188	*CASP3*
hsa-miR-30e	1222	1228	1223	*CASP3*
hsa-miR-204	2304	2310	2305	*CHP*
hsa-miR-181a	104	110	105	*DERL1*
hsa-miR-181a	1185	1191	1186	*DERL1*
hsa-miR-181b	104	110	105	*DERL1*
hsa-miR-181b	1185	1191	1186	*DERL1*
hsa-miR-181c	104	110	105	*DERL1*
hsa-miR-181c	1185	1191	1186	*DERL1*
hsa-miR-103	299	305	300	*MAP2K3*
hsa-miR-107	299	305	300	*MAP2K3*
hsa-miR-15a	231	237	231	*MAP2K3*
hsa-miR-15a	300	306	300	*MAP2K3*
hsa-miR-16	231	237	231	*MAP2K3*
hsa-miR-16	300	306	300	*MAP2K3*

hsa-miR-195	231	237	231	*MAP2K3*
hsa-miR-195	300	306	300	*MAP2K3*
hsa-miR-214	229	235	229	*MAP2K3*
hsa-miR-381	379	385	379	*MAP2K3*
hsa-miR-9	168	174	168	*MAP2K3*
hsa-miR-29a	329	335	329	*MAP2K6*
hsa-miR-29b	329	335	329	*MAP2K6*
hsa-miR-29c	329	335	329	*MAP2K6*
hsa-miR-106b	97	103	99	*MAP3K5*
hsa-miR-23a	383	389	384	*MAP3K5*
hsa-miR-23b	383	389	384	*MAP3K5*
hsa-miR-30b	116	122	117	*MAP3K5*
hsa-miR-30d	116	122	117	*MAP3K5*
hsa-miR-30e	116	122	117	*MAP3K5*
hsa-miR-125b	1610	1616	1610	*MAPK14*
hsa-miR-185	62	68	62	*MAPK14*
hsa-miR-19b	2057	2063	2058	*MAPK14*
hsa-miR-19b	932	938	932	*MAPK14*
hsa-miR-22	1451	1457	1452	*MAPK14*
hsa-miR-27a	33	39	33	*MAPK14*
hsa-miR-27b	33	39	33	*MAPK14*
hsa-miR-154	399	405	399	*NEFH*
hsa-miR-32	555	561	556	*NEFH*
hsa-miR-9	29	35	29	*NEFH*
hsa-miR-125b	144	150	144	*NEFM*
hsa-miR-135b	25	31	26	*NEFM*
hsa-miR-24	285	291	286	*NEFM*
hsa-miR-25	117	123	117	*NEFM*
hsa-miR-32	117	123	118	*NEFM*
hsa-miR-101	884	890	885	*RAB5A*
hsa-miR-101	971	977	971	*RAB5A*
hsa-miR-130a	1162	1168	1162	*RAB5A*
hsa-miR-130a	829	835	829	*RAB5A*
hsa-miR-144	885	891	885	*RAB5A*
hsa-miR-101	537	543	538	*RAC1*
hsa-miR-137	987	993	987	*RAC1*
hsa-miR-142-3p	747	753	748	*RAC1*
hsa-miR-142-3p	1400	1406	1400	*RAC1*
hsa-miR-144	538	544	538	*RAC1*
hsa-miR-194	127	133	127	*RAC1*
hsa-miR-105	1531	1537	1532	*SLC1A2*

hsa-miR-105	5006	5012	5009	*SLC1A2*
hsa-miR-105	7187	7193	7190	*SLC1A2*
hsa-miR-106b	8055	8061	8058	*SLC1A2*
hsa-miR-106b	5642	5648	5645	*SLC1A2*
hsa-miR-145	9579	9585	9582	*SLC1A2*
hsa-miR-153	8051	8057	8054	*SLC1A2*
hsa-miR-19b	495	501	496	*SLC1A2*
hsa-miR-19b	5357	5363	5360	*SLC1A2*
hsa-miR-19b	6611	6617	6614	*SLC1A2*
hsa-miR-221	4642	4648	8066	*SLC1A2*
hsa-miR-27a	6735	6741	6739	*SLC1A2*
hsa-miR-27a	2290	2296	2293	*SLC1A2*
hsa-miR-27a	8284	8290	8287	*SLC1A2*
hsa-miR-27b	6735	6741	6739	*SLC1A2*
hsa-miR-27b	2290	2296	2293	*SLC1A2*
hsa-miR-27b	8284	8290	8287	*SLC1A2*
hsa-miR-29b	7320	7326	7323	*SLC1A2*
hsa-miR-29c	7320	7326	7323	*SLC1A2*
hsa-miR-31	276	282	277	*SLC1A2*
hsa-miR-31	7556	7562	7559	*SLC1A2*
hsa-miR-31	3180	3186	3183	*SLC1A2*
hsa-miR-381	6651	6657	6654	*SLC1A2*
hsa-miR-93	8055	8061	8058	*SLC1A2*
hsa-miR-93	5642	5648	5645	*SLC1A2*
hsa-miR-377	107	113	107	*SOD1*
hsa-miR-29a	116	122	118	*TNFRSF1A*
hsa-miR-29b	116	122	118	*TNFRSF1A*
hsa-miR-29c	116	122	118	*TNFRSF1A*

Table S6: Comparison of target site prediction results obtained using miRanda and Pictar.

	**miRanda**		**PicTar**	
**miRNA**	**Start position of target site**	**End position of target site**	**Start position of target site**	**Gene name**
hsa-miR-103	286	307	300	*MAP2K3*
hsa-miR-107	283	307	300	*MAP2K3*
hsa-miR-195	2520	2537	2530	*BCL2*

Table S7 : Hot spots identified in selected genes in 5'UTR , CDS and 3' UTR for miRNAs considered in the study.

S. No	Gene symbol	Hotspots at 5′ region	Hotspots at CDS region	Hotspots at 3′ region	Total
1	*BAX*	1	-	-	1
2	*DERL1*	1	-	-	1
3	*CYCS*	-	-	1	1
4	*CASP9*	-	2	-	2
5	*TNF*	-	1	-	1
6	*SLC1A2*	1	-	-	1
7	*RAB5A*	1	-	-	1
8	*MAPK14*	-	1	-	1
9	*MAP2K3*	-	1	1	2
	Total	4	5	2	11
