# Supplementary material for: A Complex Network of MicroRNAs Expressed in Brain and Genes Associated with Amyotrophic Lateral Sclerosis
Source: Int J Genomics. 2013 Jul 10;2013:383024. doi: 10.1155/2013/383024 (PMC3723150; doi:10.1155/2013/383024)
Supplement: Supplementary file 1 — Table S1: Information of Amyotrophic Lateral Sclerosis Disease associated genes selected from KEGG pathway database. Table S2: Distribution of target sites predicted across gene in 5' UTR, CDS and 3' UTR for miRNAs expressed in midbrain, cerebellum, frontal cortex and hippocampus predicted by miRanda. Table S3: Target sites predicted for miRNAs expressed in midbrain, cerebellum, hippocampus and frontal cortex using TargetScan. Table S4: Target sites predicted for miRNAs expressed in midbrain, cerebellum, hippocampus and frontal cortex using Pictar. Table S5: Comparison of target site prediction results obtained using TargetScan and Pictar. Table S6: Comparison of target site prediction results obtained using miRanda and Pictar. Table S7: Hot spots identified in selected genes in 5'UTR , CDS and 3' UTR for miRNAs considered in the study. Figure SF1: Schematic representation of miRNA Target sites on ALS2 Figure SF2: Schematic representation of miRNA Target sites on APAF1 Figure SF3: Schematic representation of miRNA Target sites on BAD Figure SF4: Schematic representation of miRNA Target sites on BAX Figure SF 5: Schematic representation of miRNA Target sites on BCL2 Figure SF 6: Schematic representation of miRNA Target sites on BCL2L1 Figure SF7: Schematic representation of miRNA Target sites on BID Figure SF8: Schematic representation of miRNA Target sites on CASP1 Figure SF8: Schematic representation of miRNA Target sites on CASP1 Figure SF9: Schematic representation of miRNA targets on GRIA1 Figure SF10: Schematic representation of miRNA Target sites on GPX1 Figure SF11: Schematic representation of miRNA Target sites on DERL1 Figure SF12: Schematic representation of miRNA Target sites on DAXX Figure SF13: Schematic representation of miRNA Target sites on CYCS Figure SF14. Schematic representation of miRNA targets on CHP Figure SF15: Schematic representation of miRNA Target sites on CCS Figure SF16: Schematic representation of miRNA Target sites on CAT Figure SF17: [file 383024.f1.zip › f1.383024/SF1-37 Supplementary Figures.pdf]

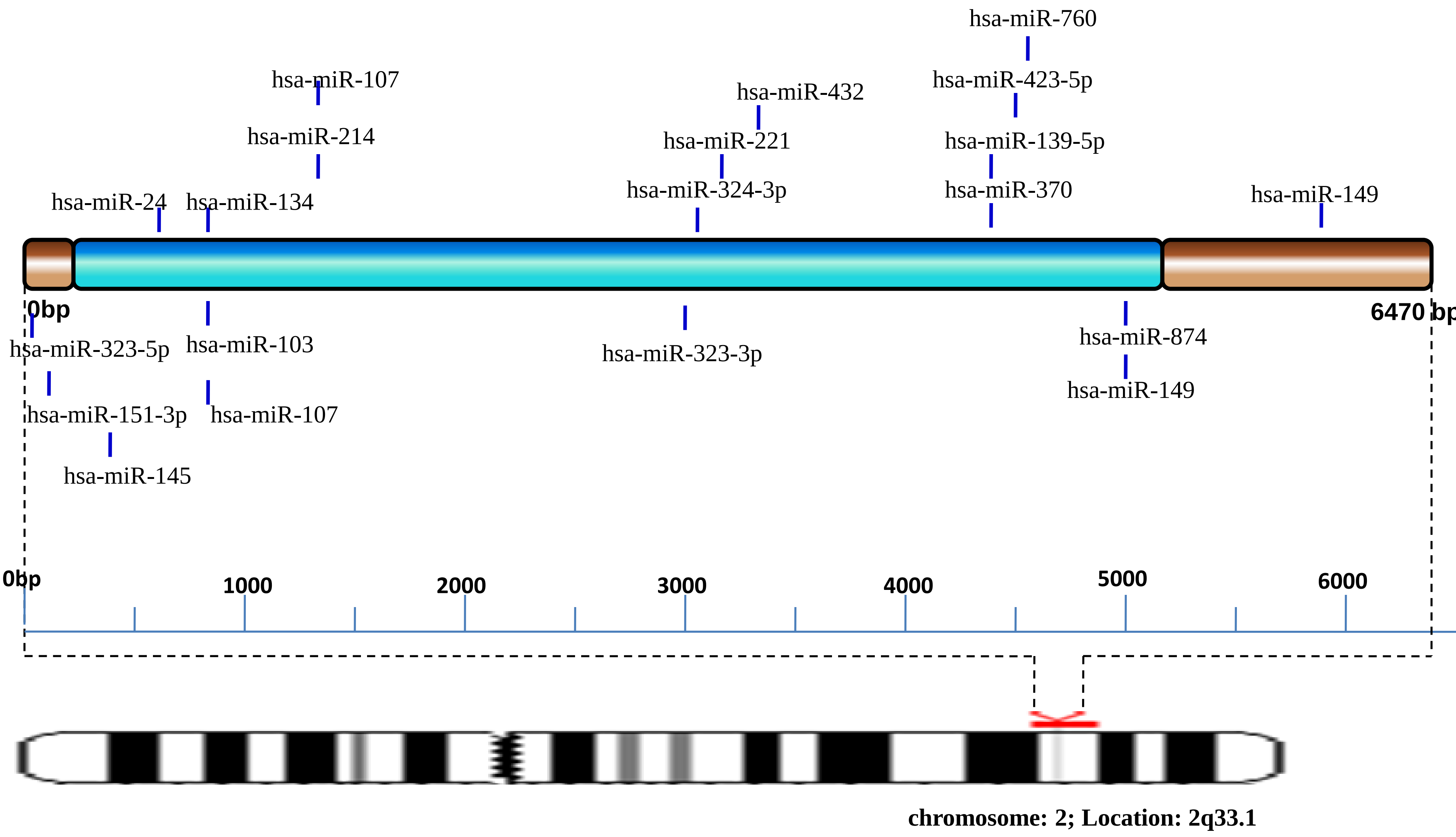

Figure SF1. Schematic representation of miRNA Target sites on ALS2

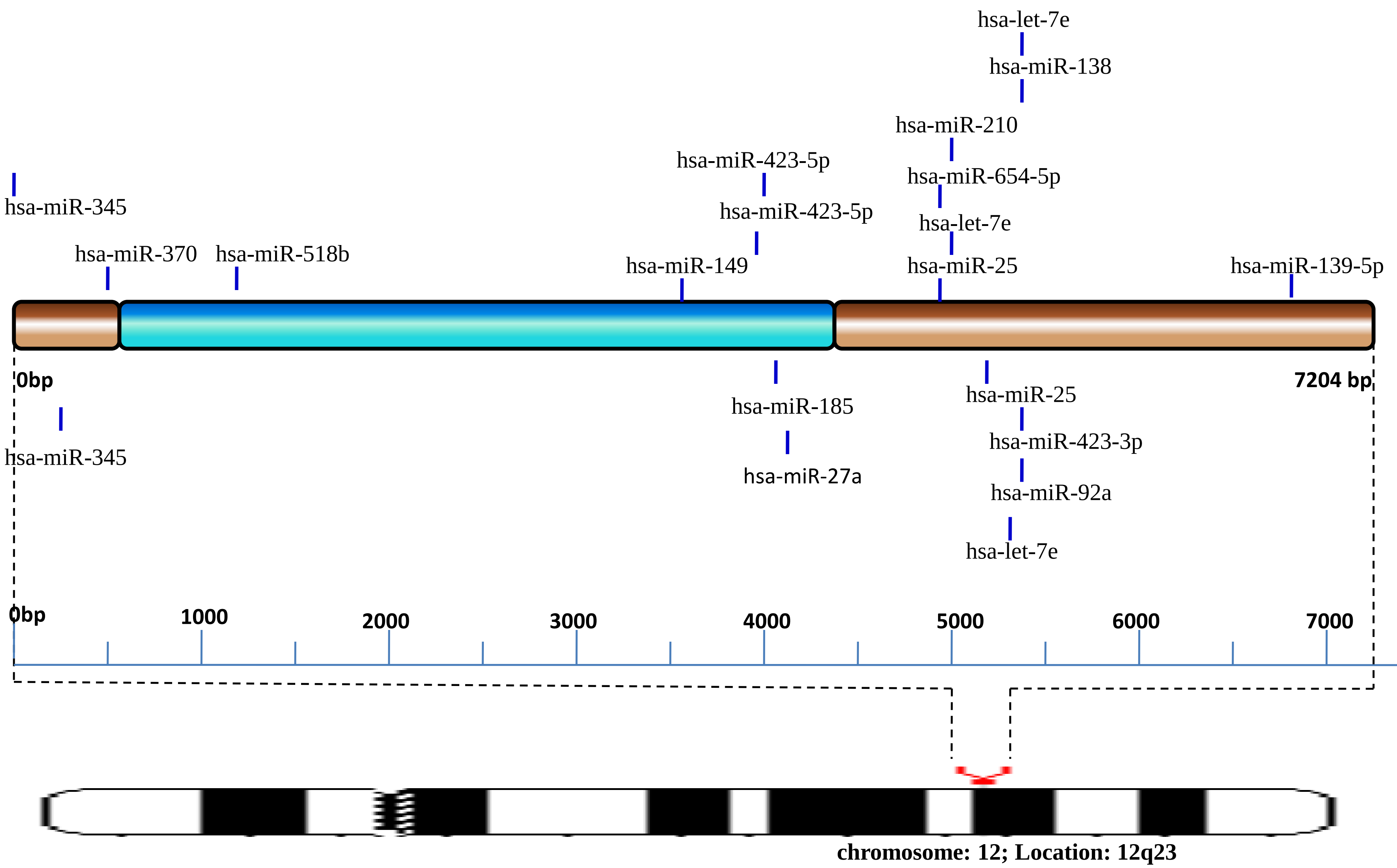

Figure SF2. Schematic representation of miRNA Target sites on APAF1

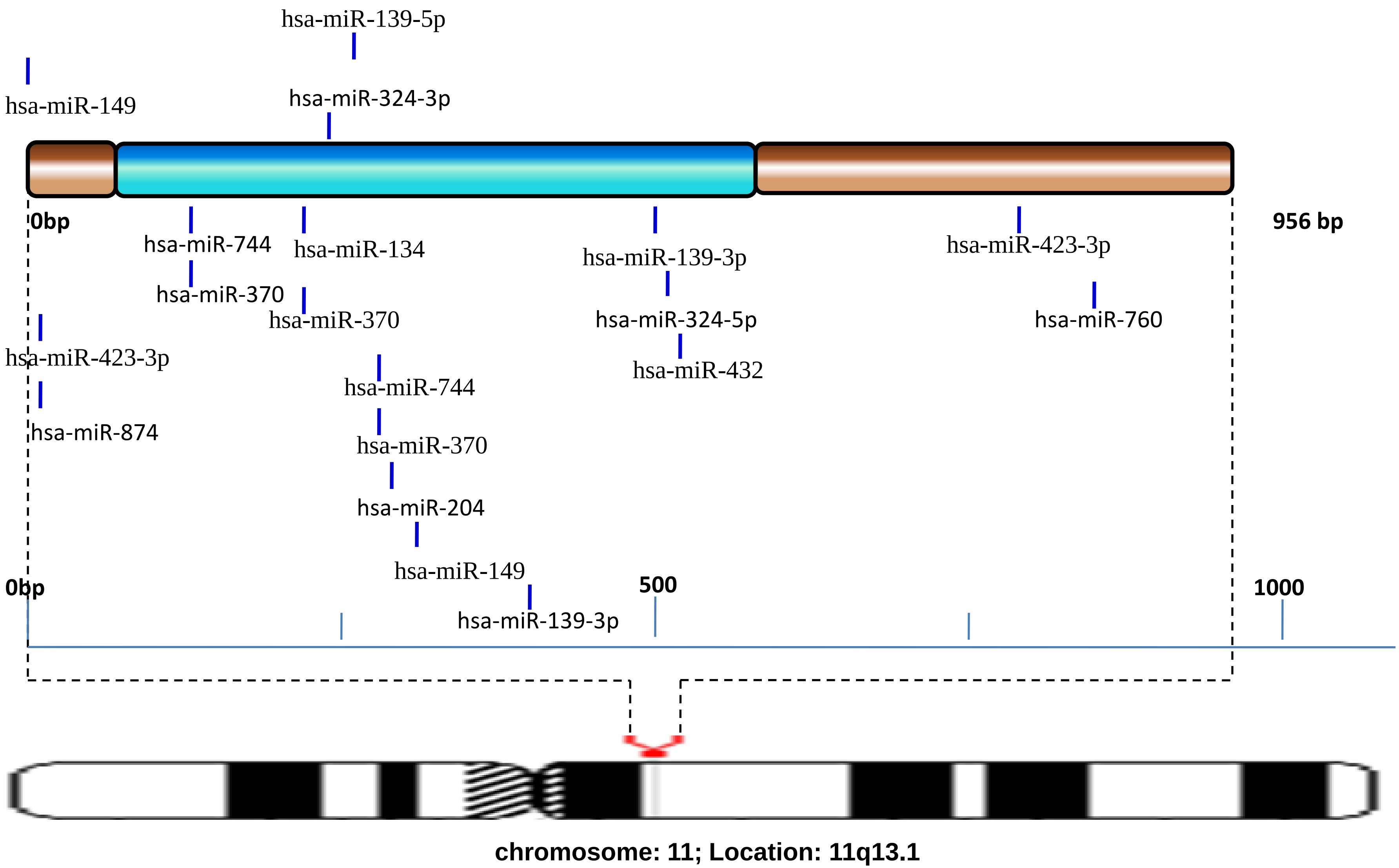

Figure SF3. Schematic representation of miRNA Target sites on BAD

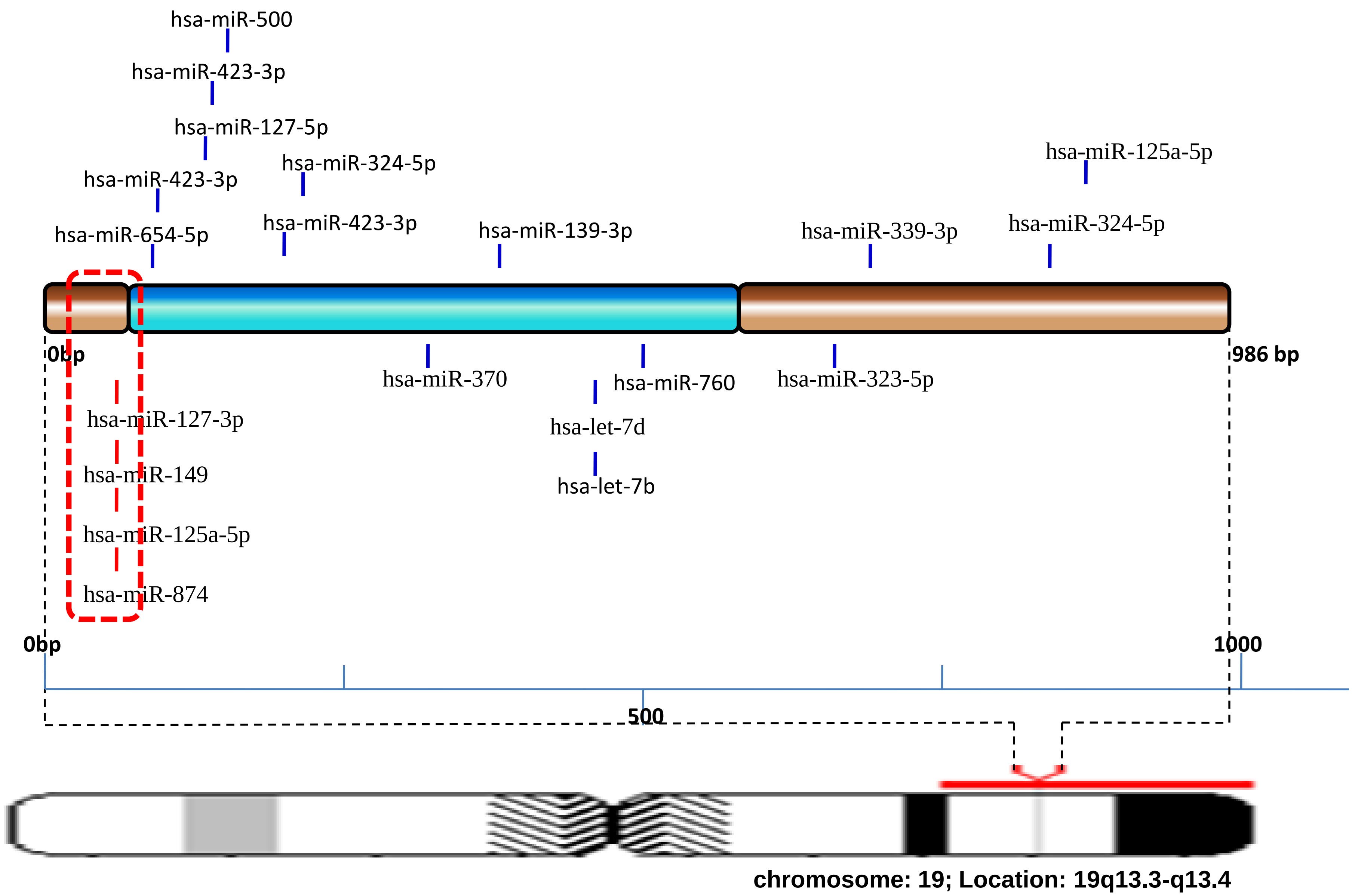

Figure SF4. Schematic representation of miRNA Target sites on BAX

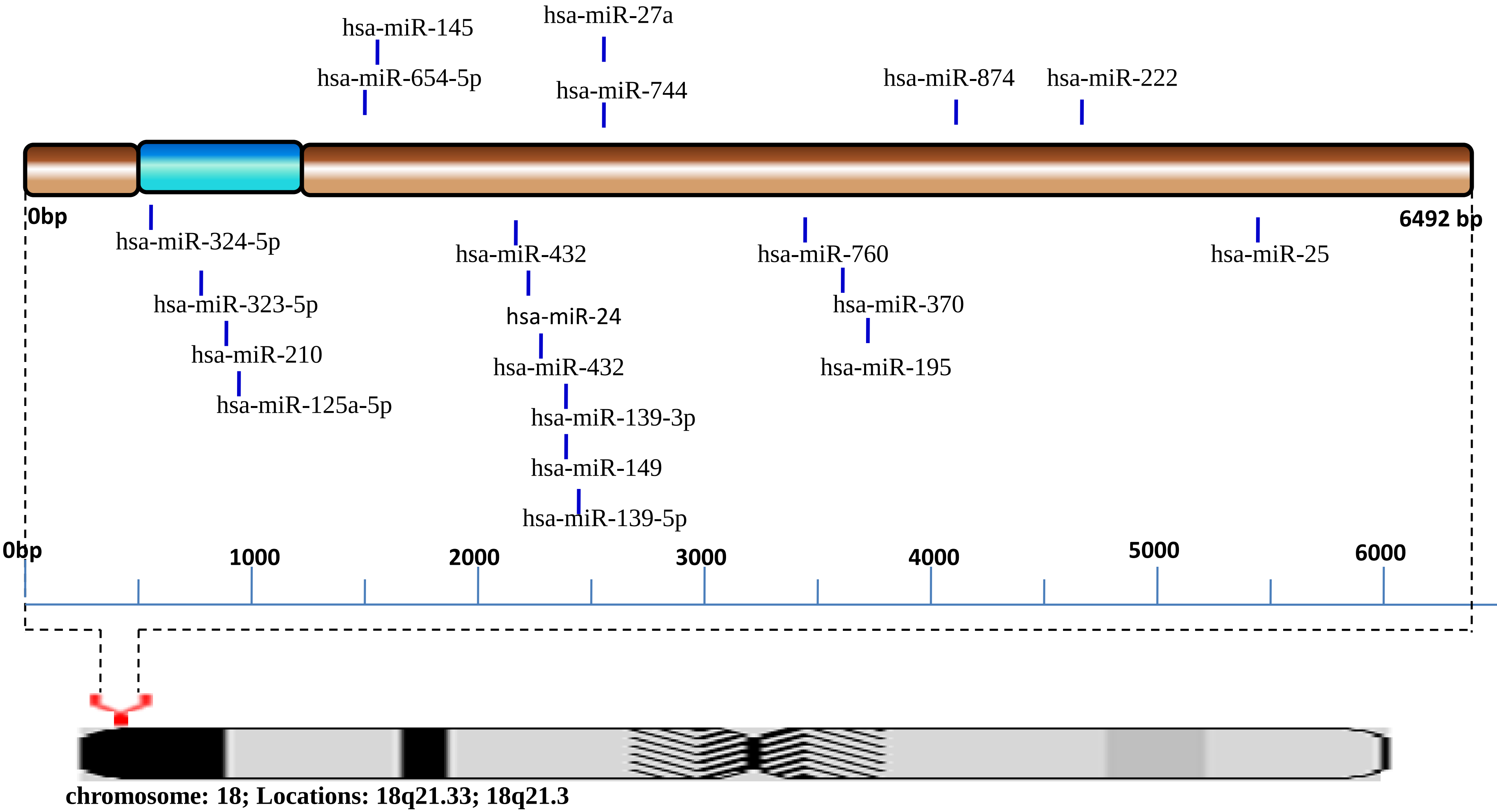

Figure SF 5. Schematic representation of miRNA Target sites on BCL2

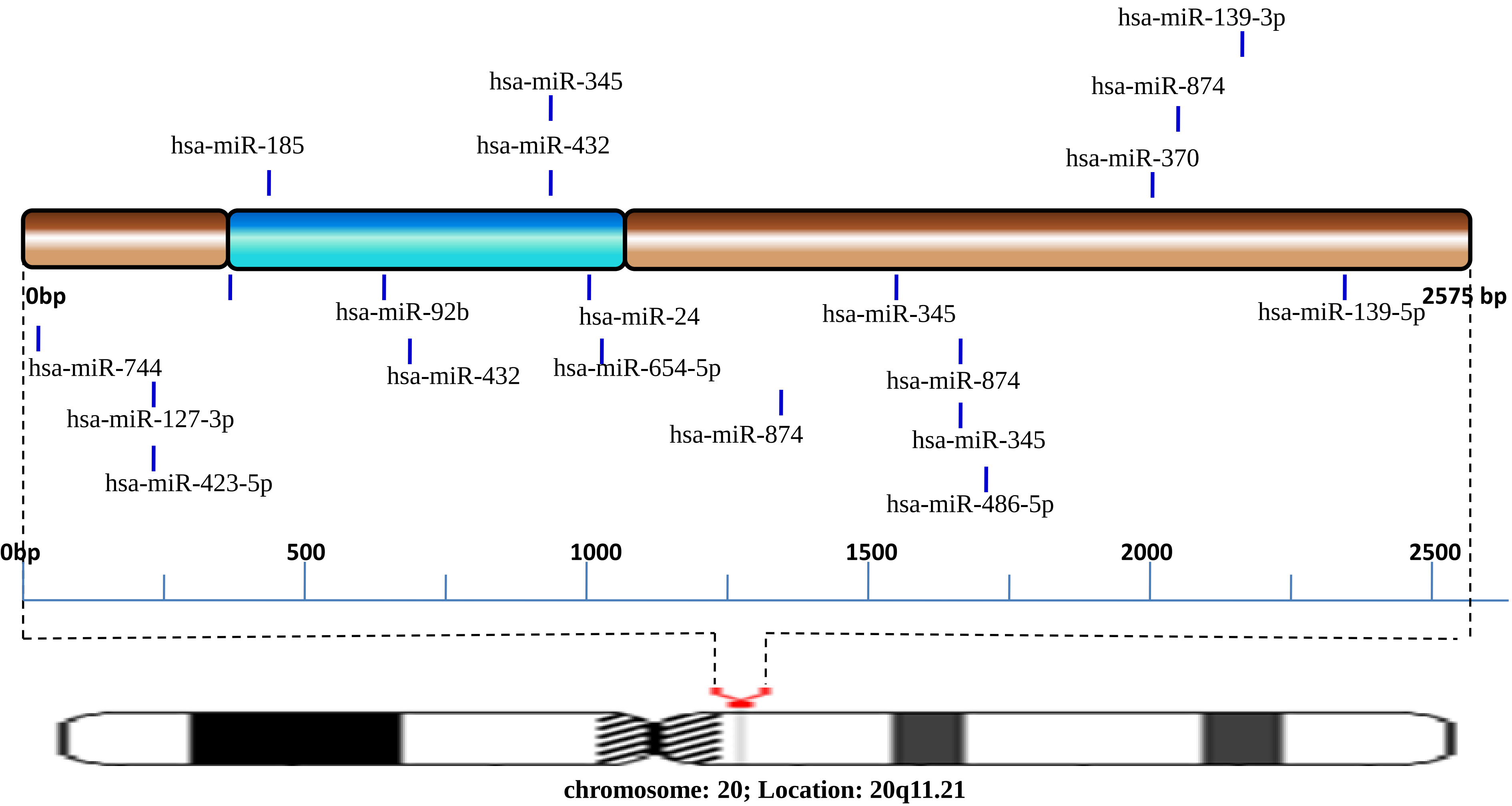

Figure SF 6. Schematic representation of miRNA Target sites on BCL2L1

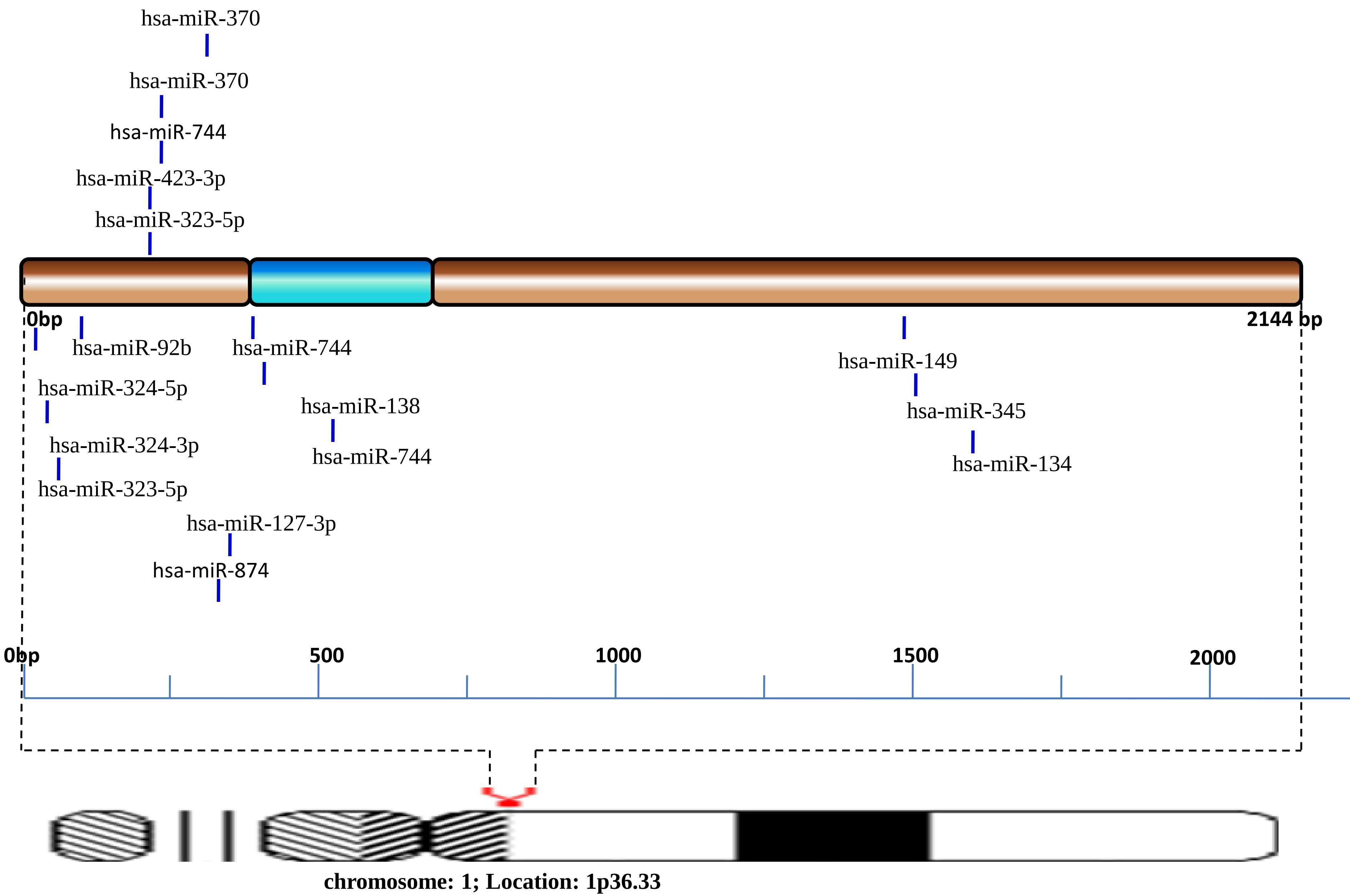

Figure SF7. Schematic representation of miRNA Target sites on BID

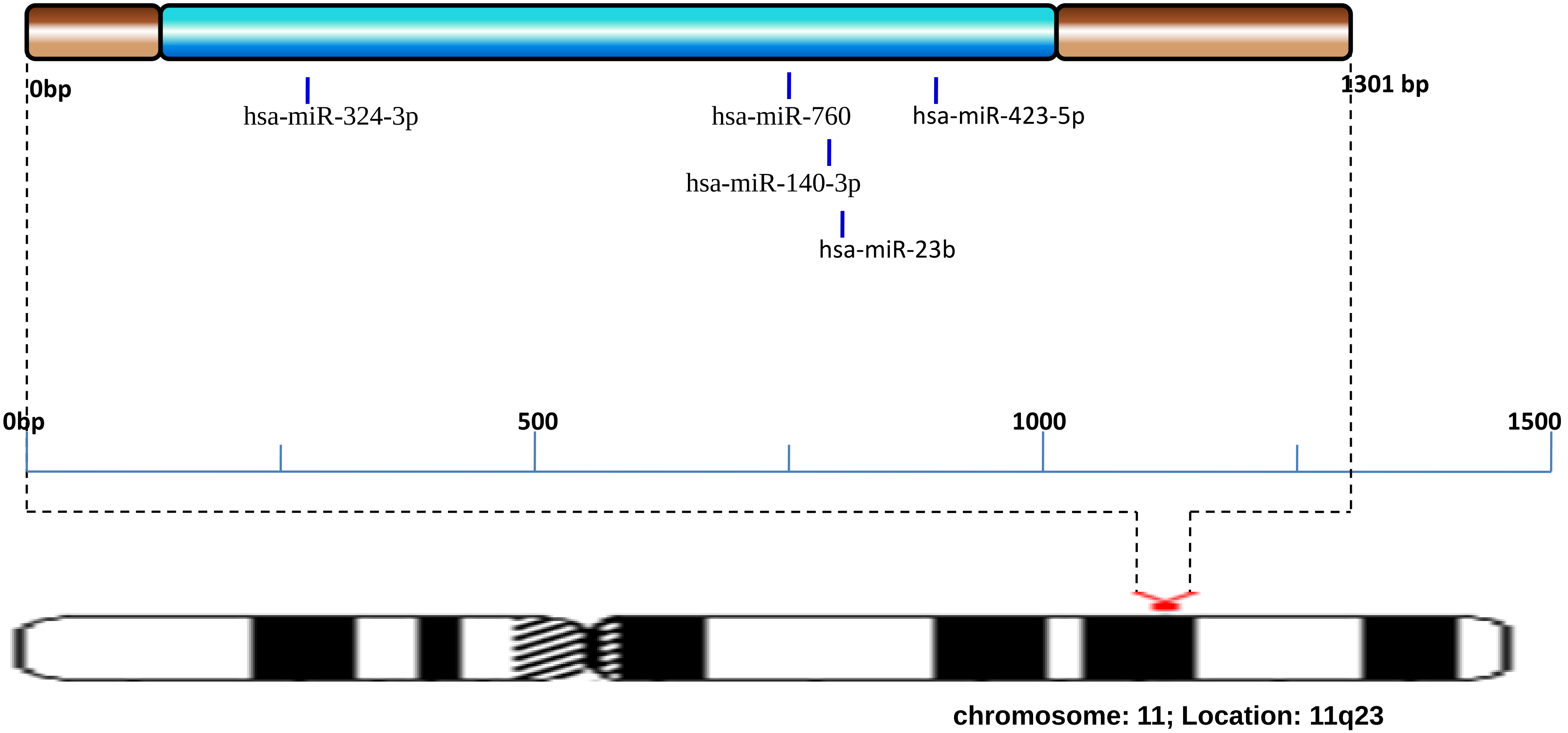

Figure SF8. Schematic representation of miRNA Target sites on CASP1

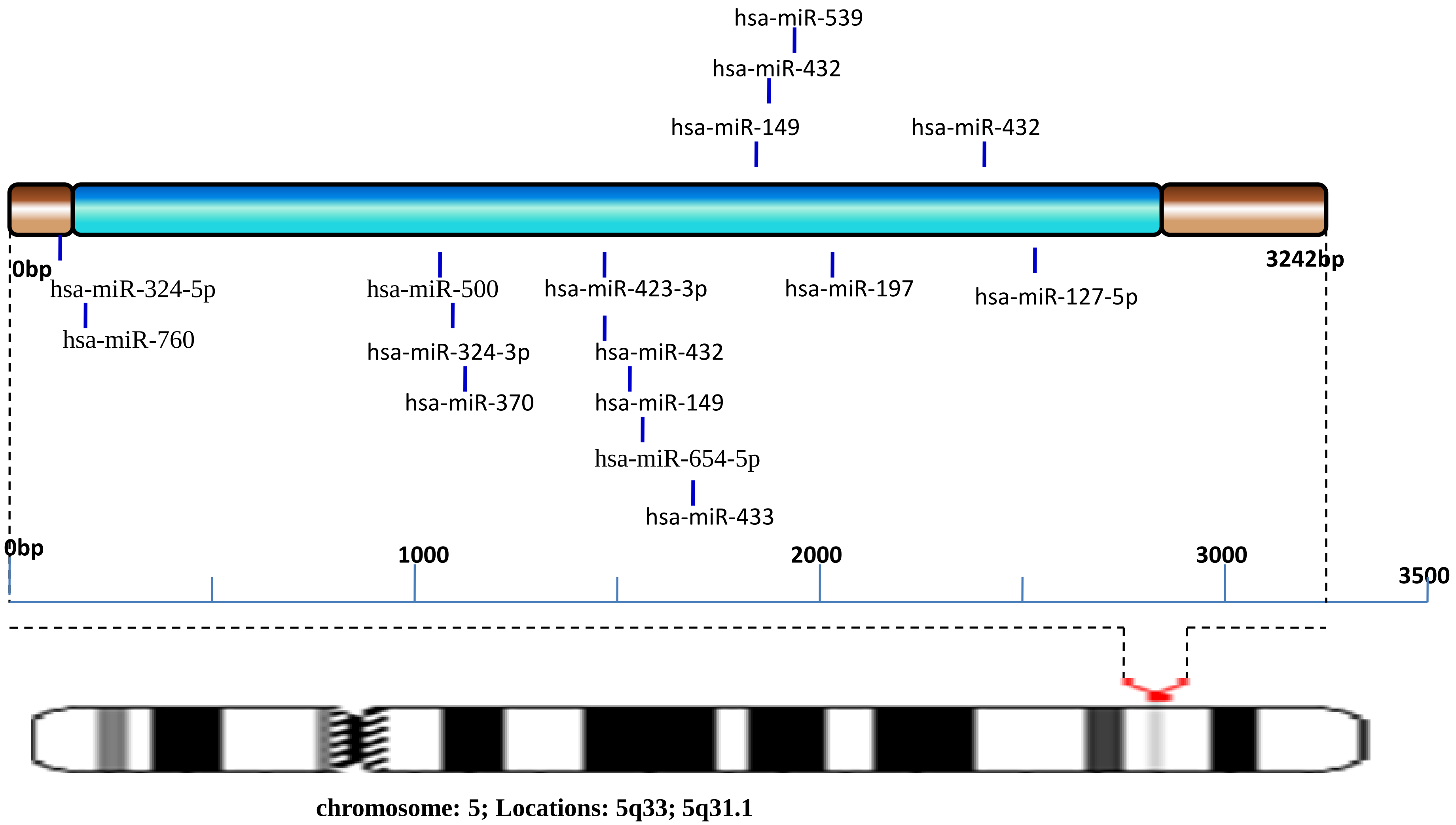

Figure SF9. Schematic representation of miRNA targets on GRIA1

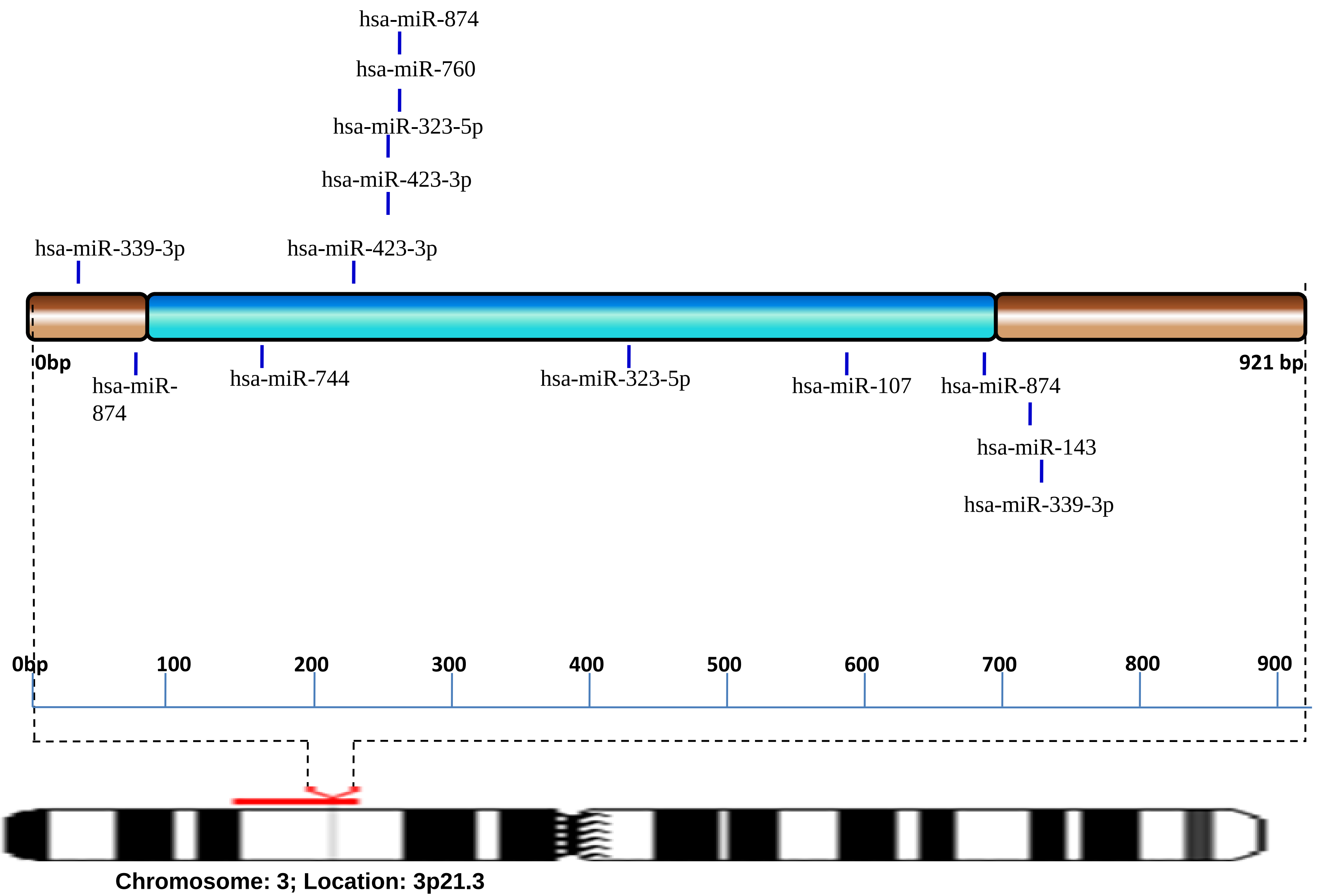

Figure SF10. Schematic representation of miRNA Target sites on GPX1

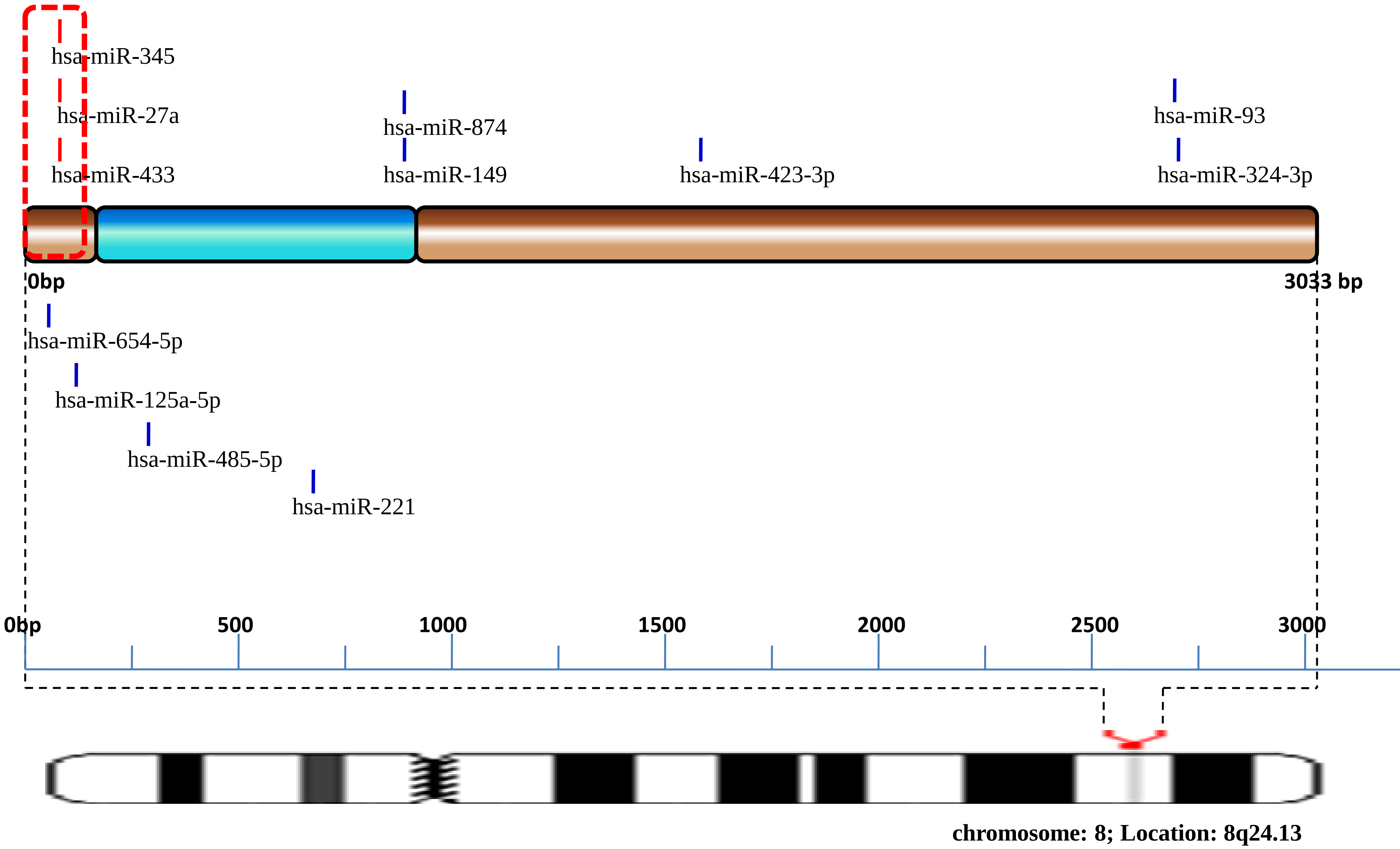

Figure SF11. Schematic representation of miRNA Target sites on DERL1

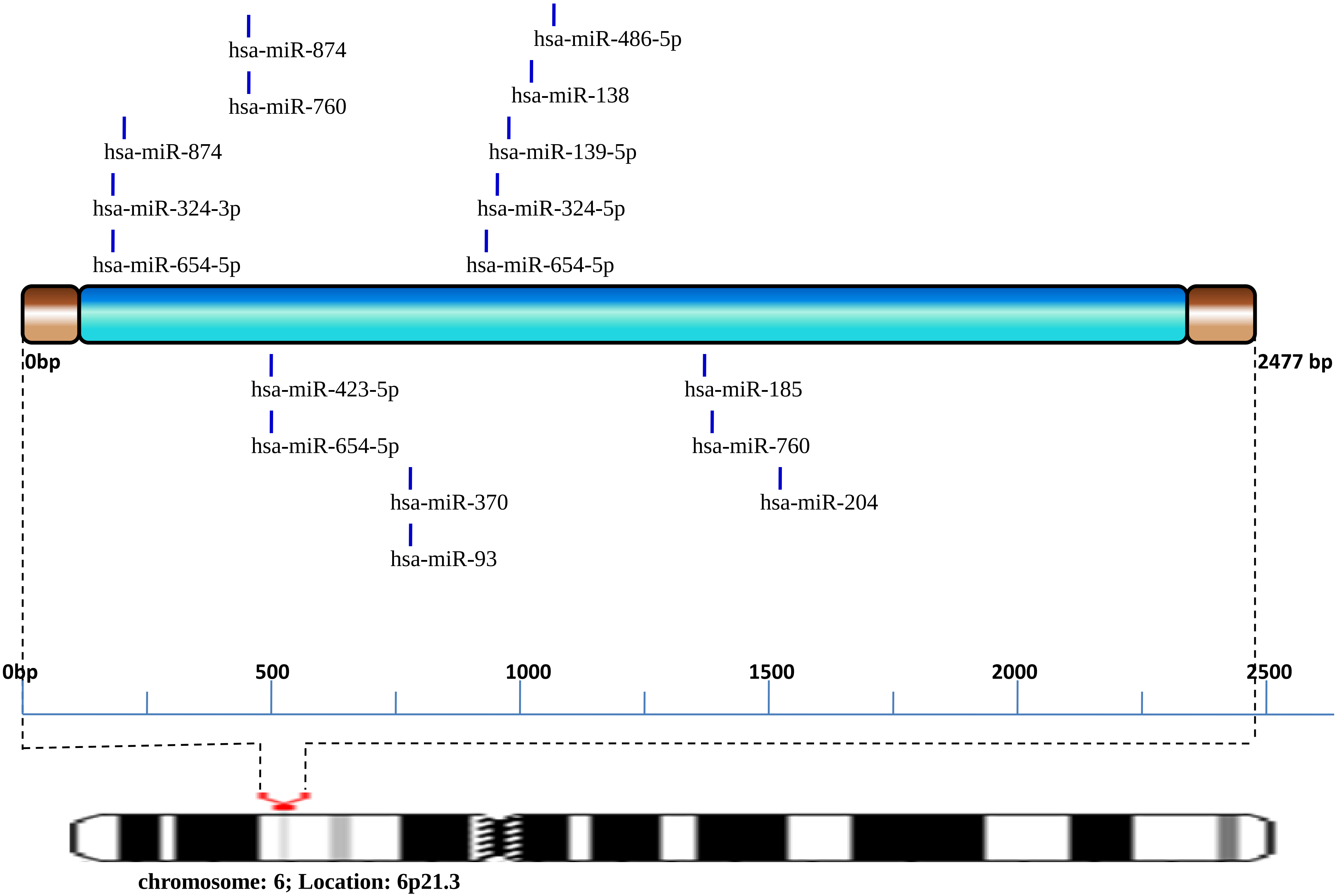

Figure SF12. Schematic representation of miRNA Target sites on DAXX

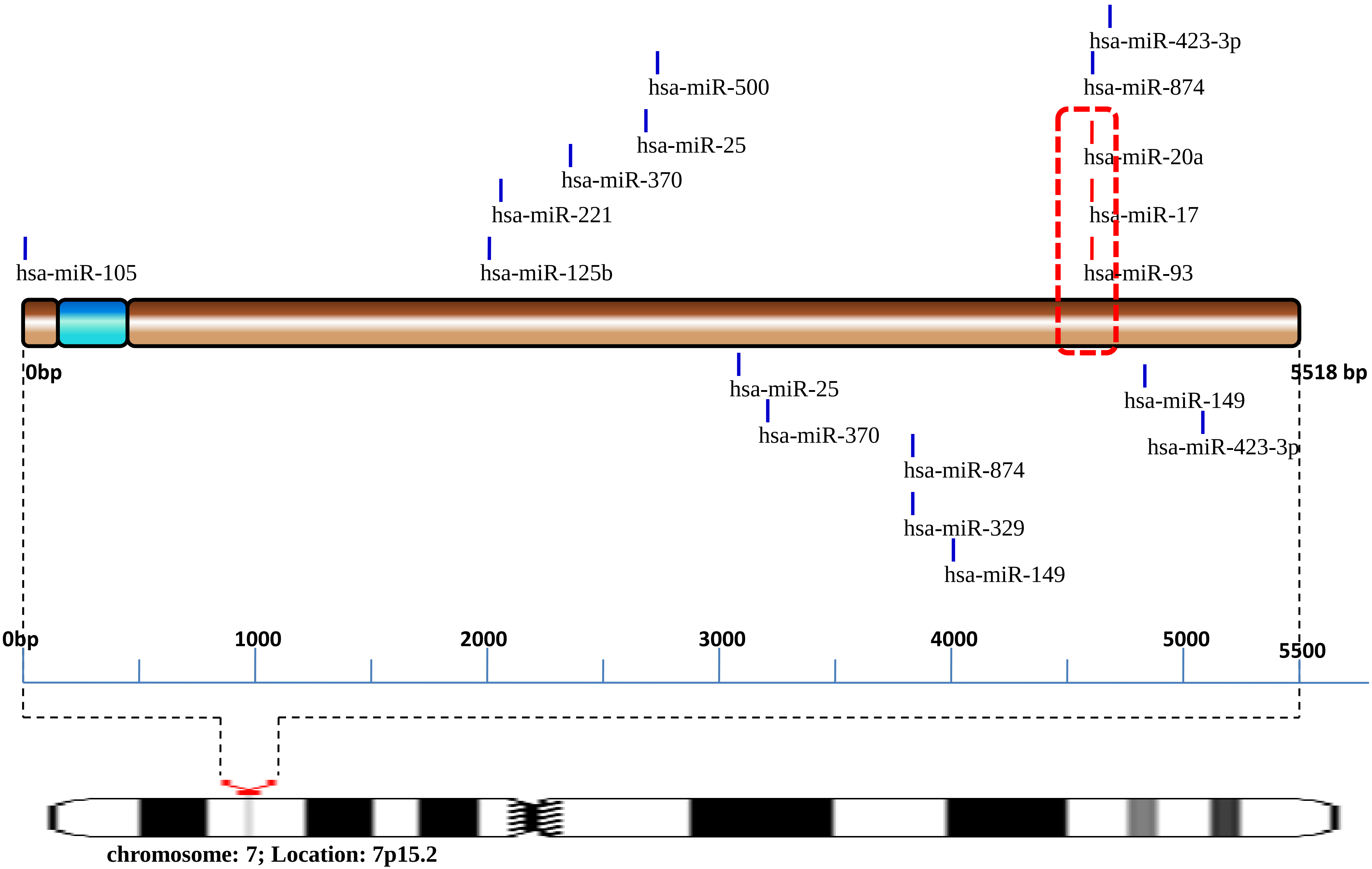

Figure SF13.- Schematic representation of miRNA Target sites on CYCS

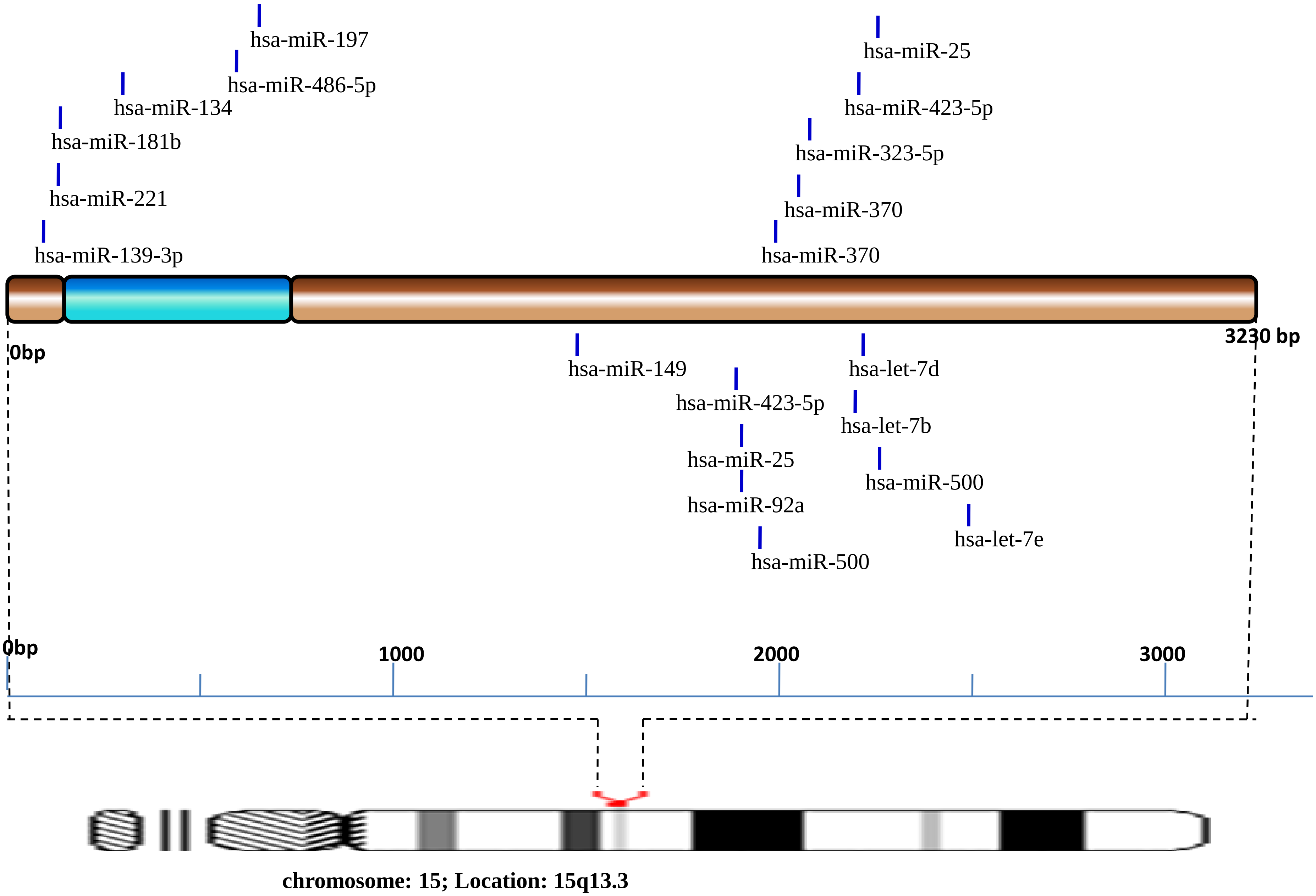

Figure SF14. Schematic representation of miRNA targets on CHP

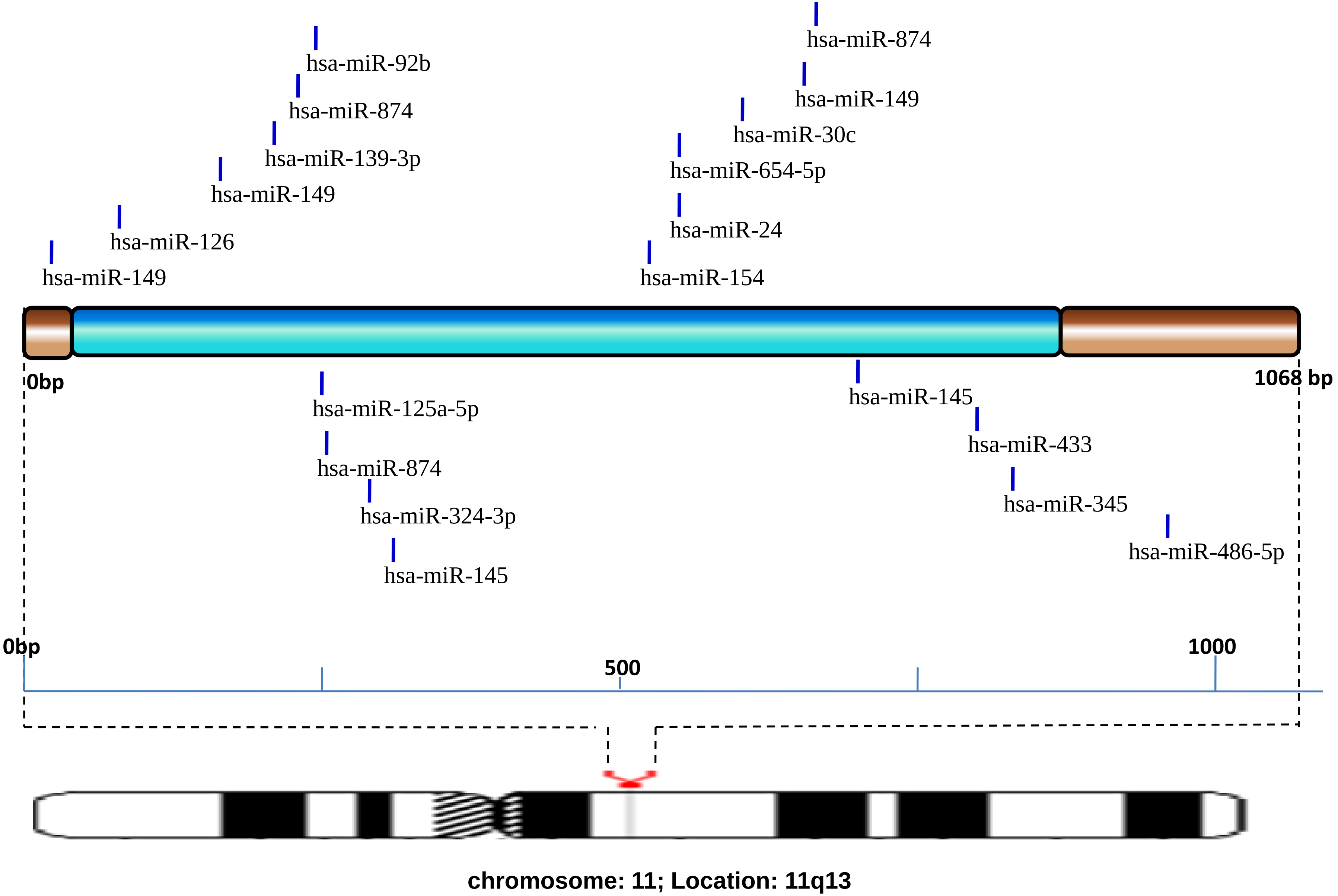

Figure SF15. Schematic representation of miRNA Target sites on CCS

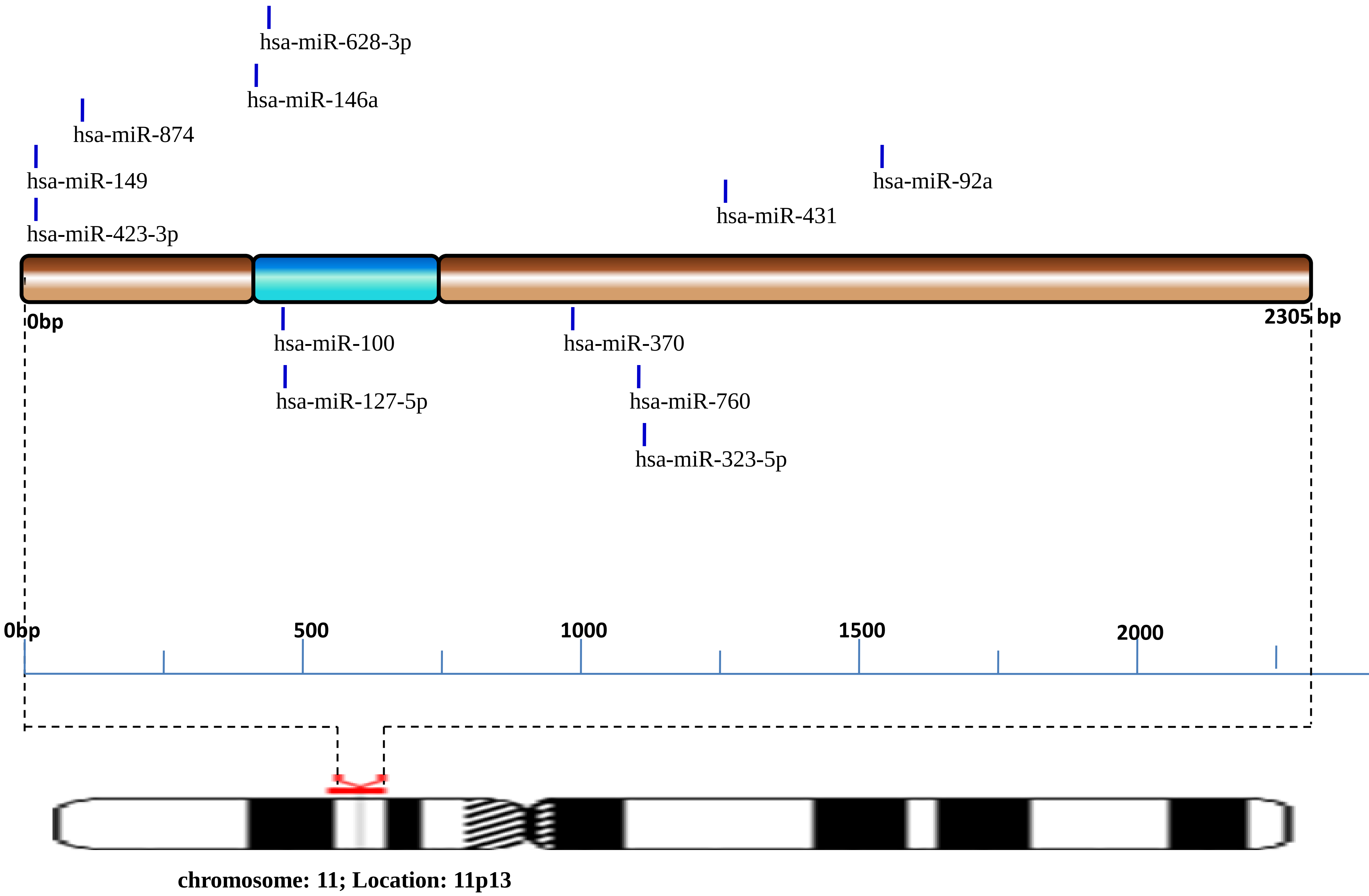

Figure SF16. Schematic representation of miRNA Target sites on CAT

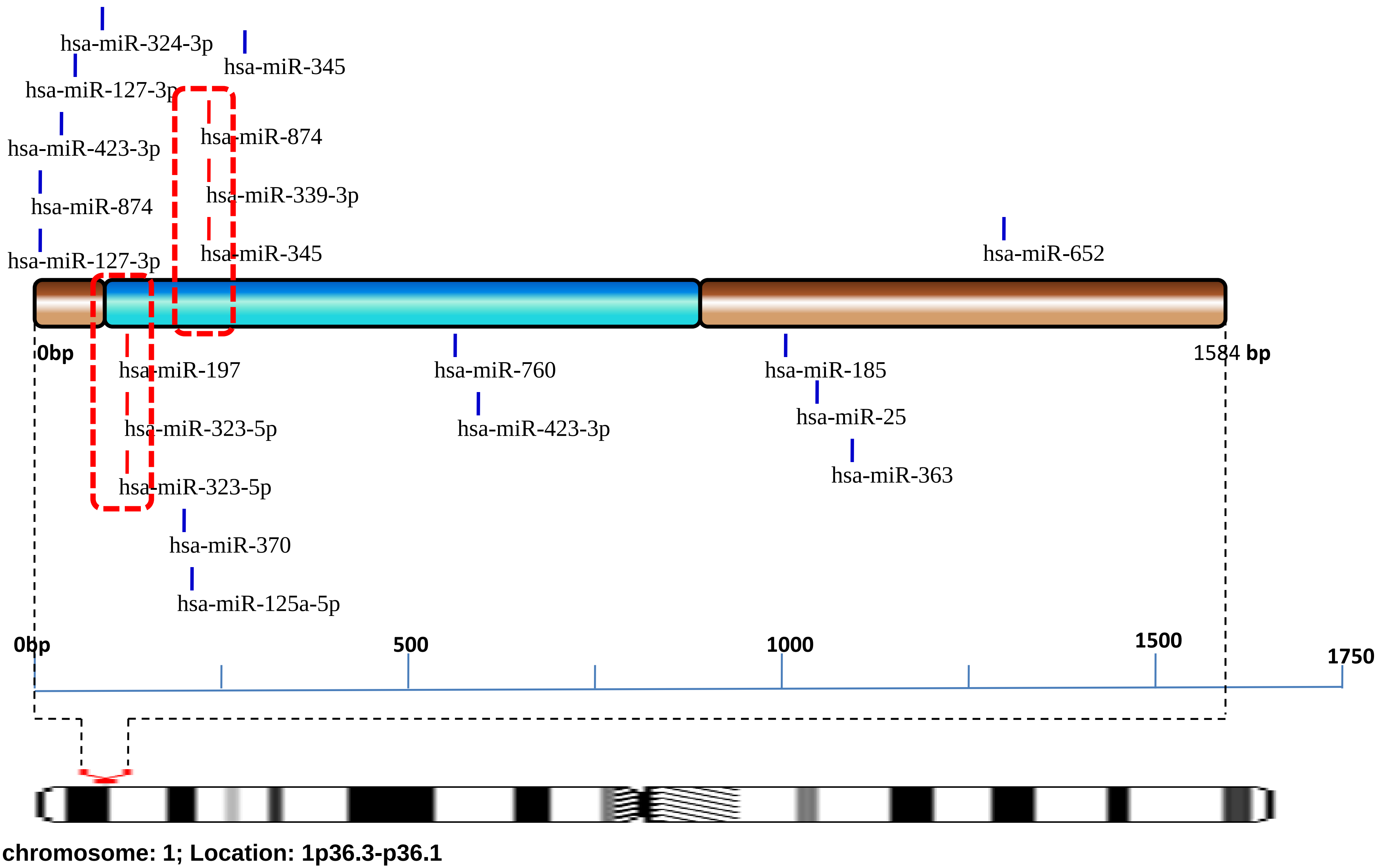

Figure SF17. Schematic representation of miRNA Target sites on CASP9

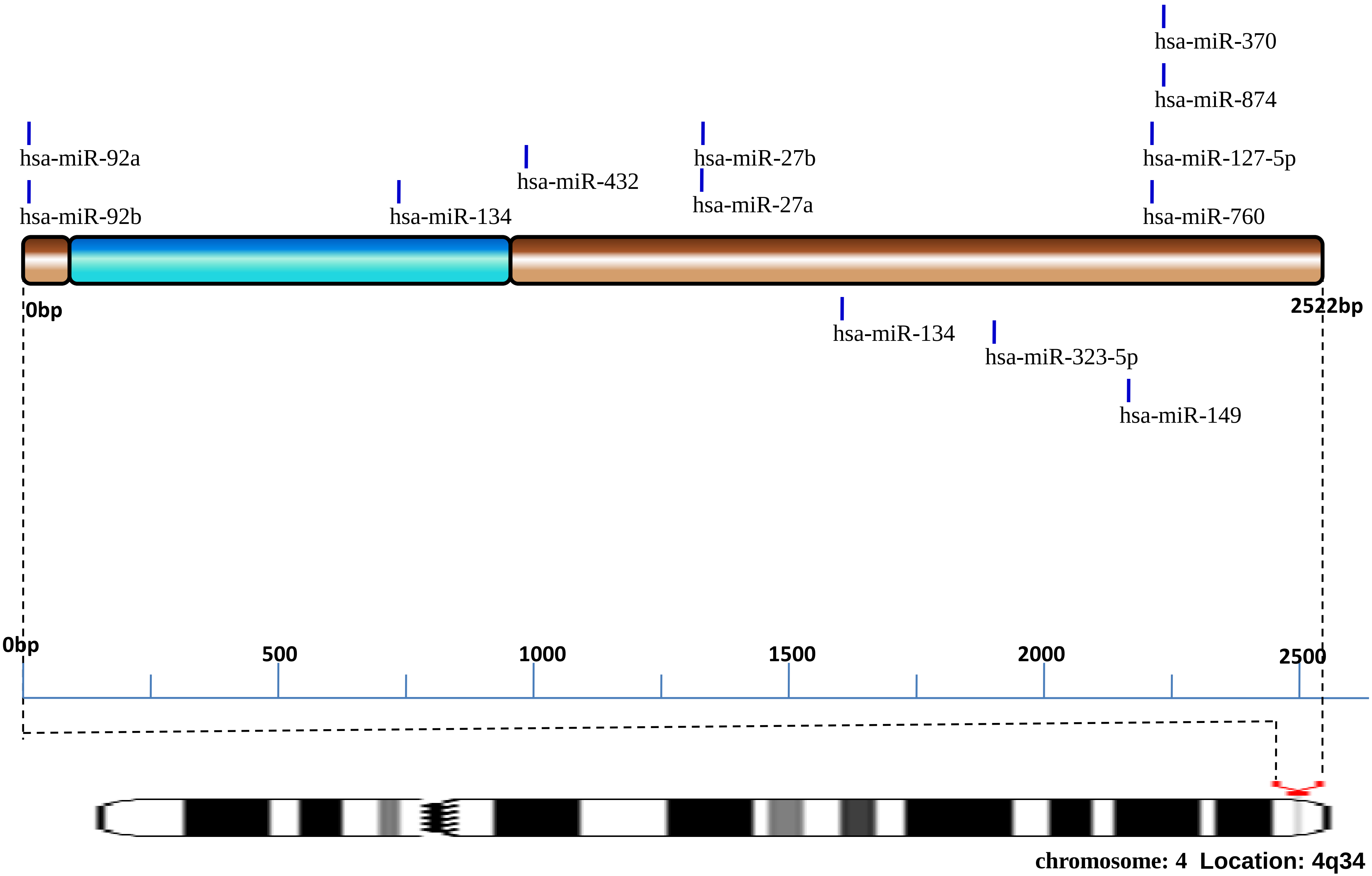

Figure SF18. Schematic representation of miRNA Target sites on CASP3

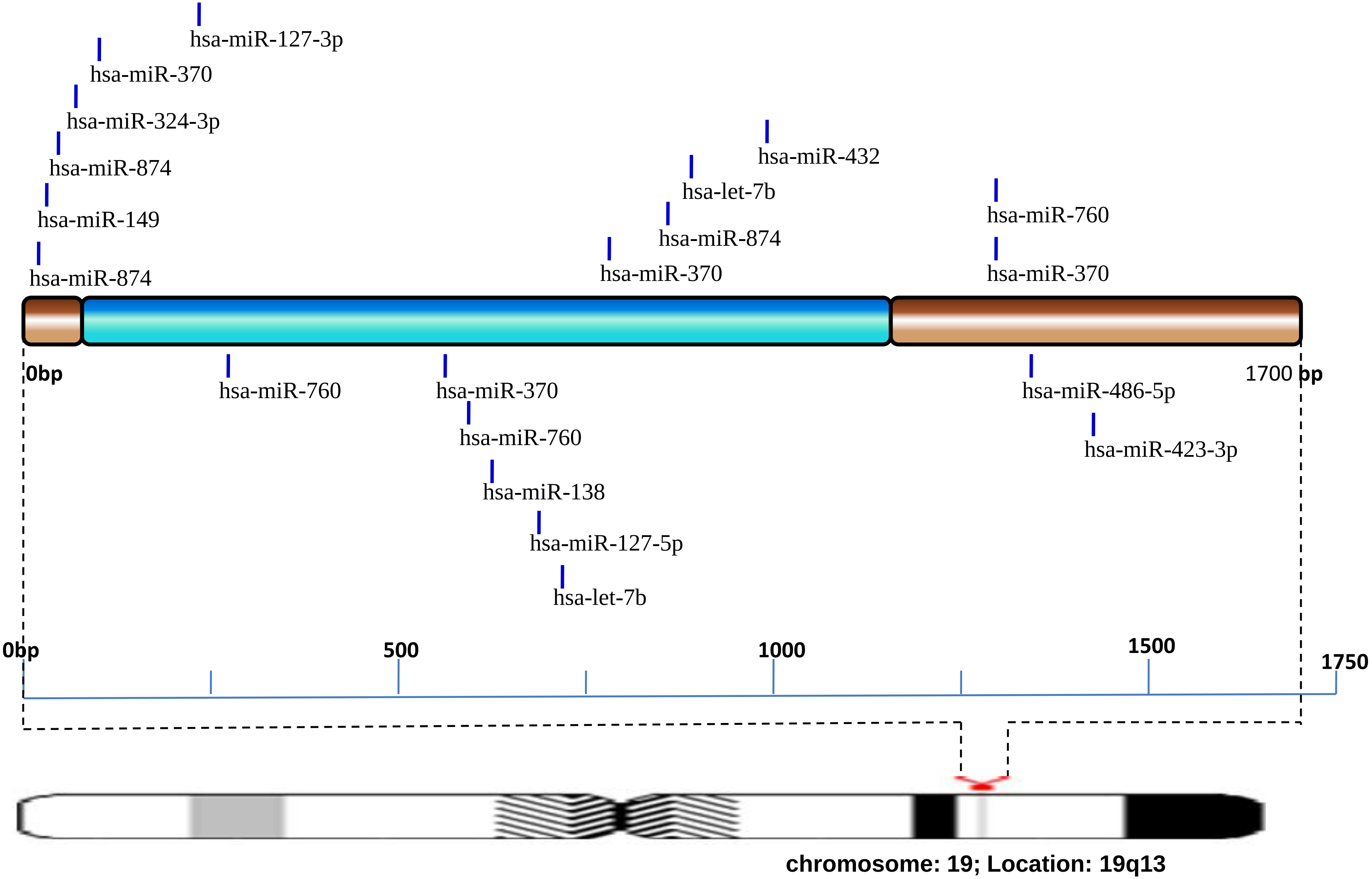

Figure SF19. Schematic representation of miRNA Target sites on TOMM40

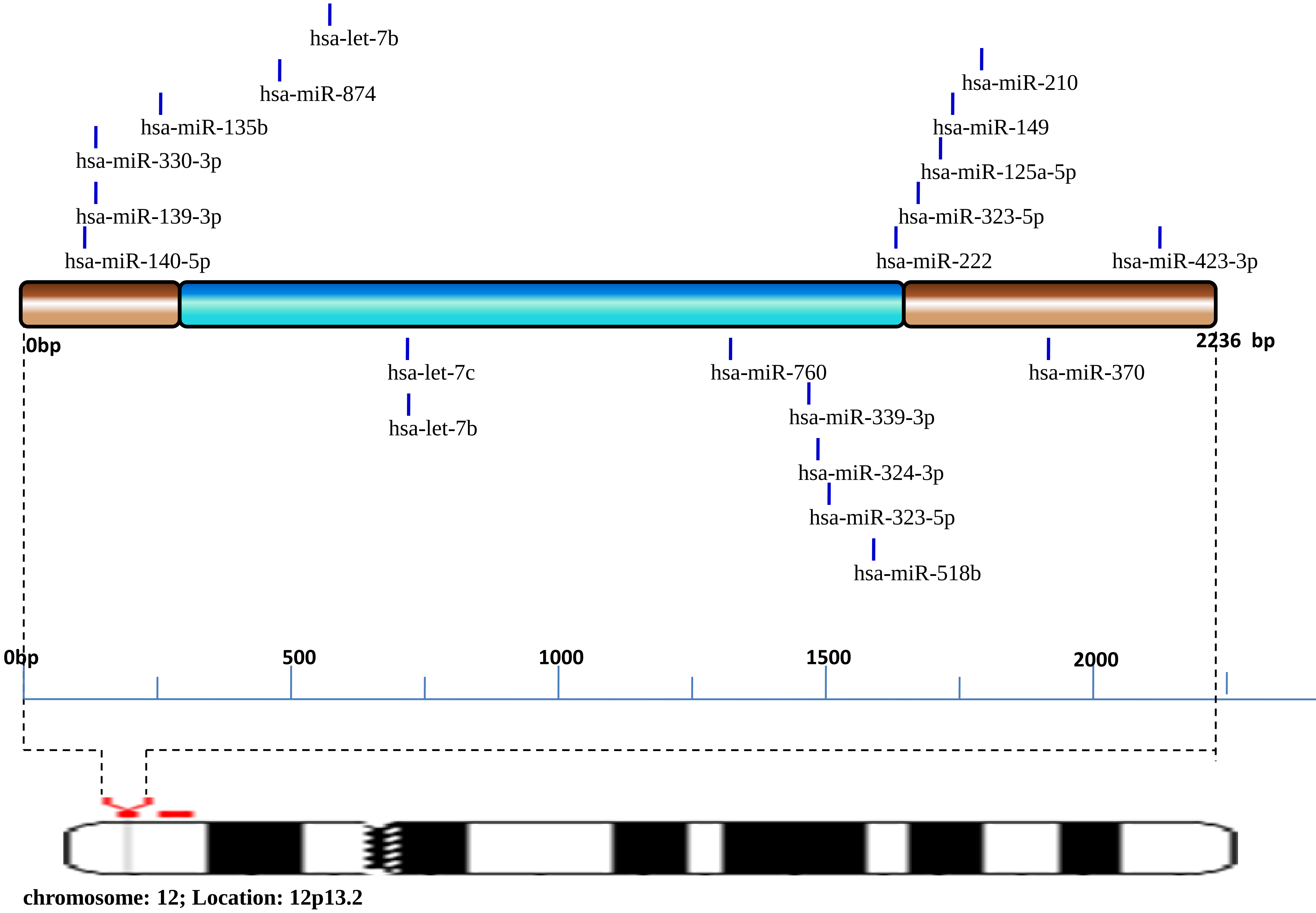

Figure SF20. Schematic representation of miRNA Target sites on TNFRSF1A

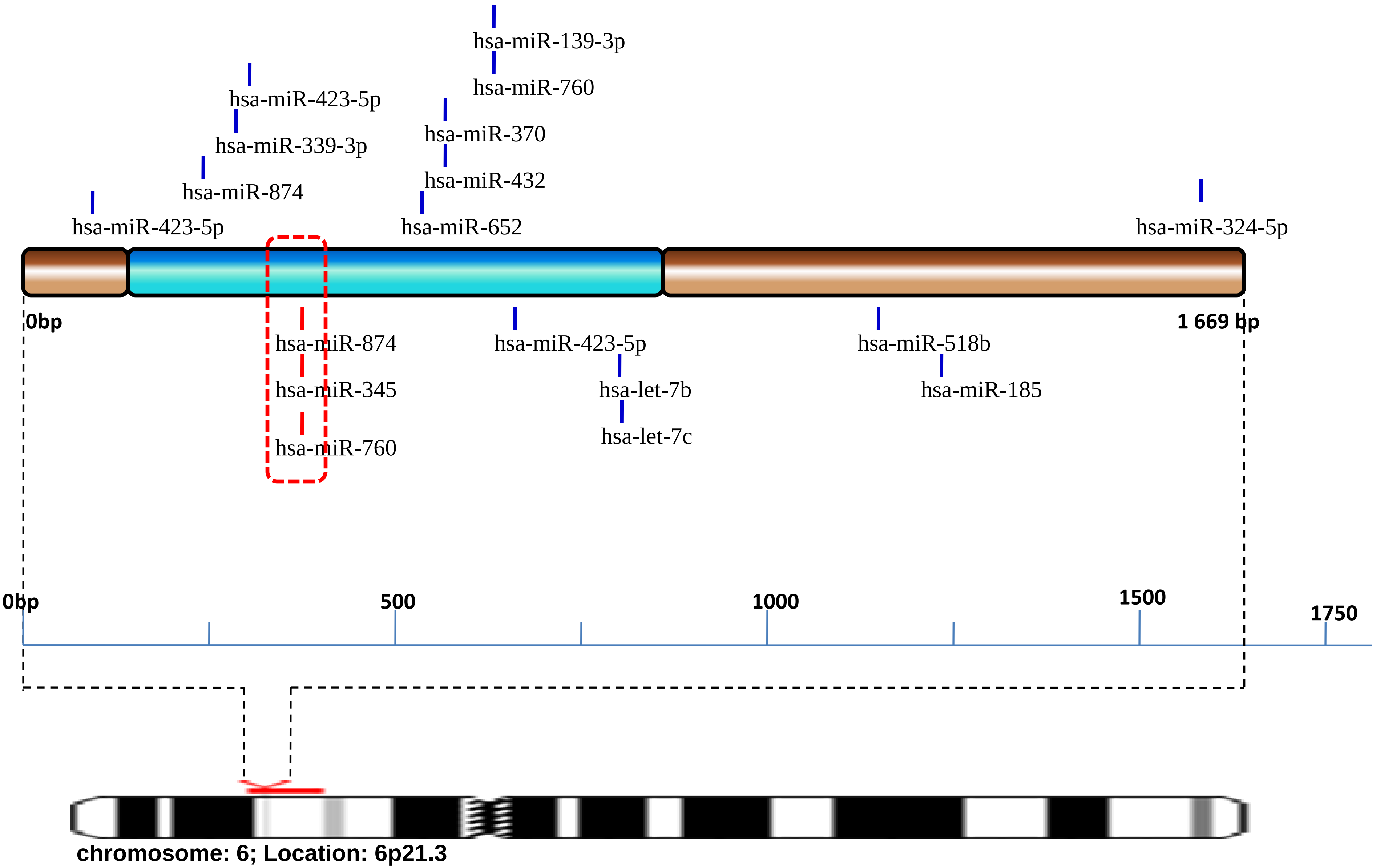

Figure SF21. Schematic representation of miRNA Target sites on TNF

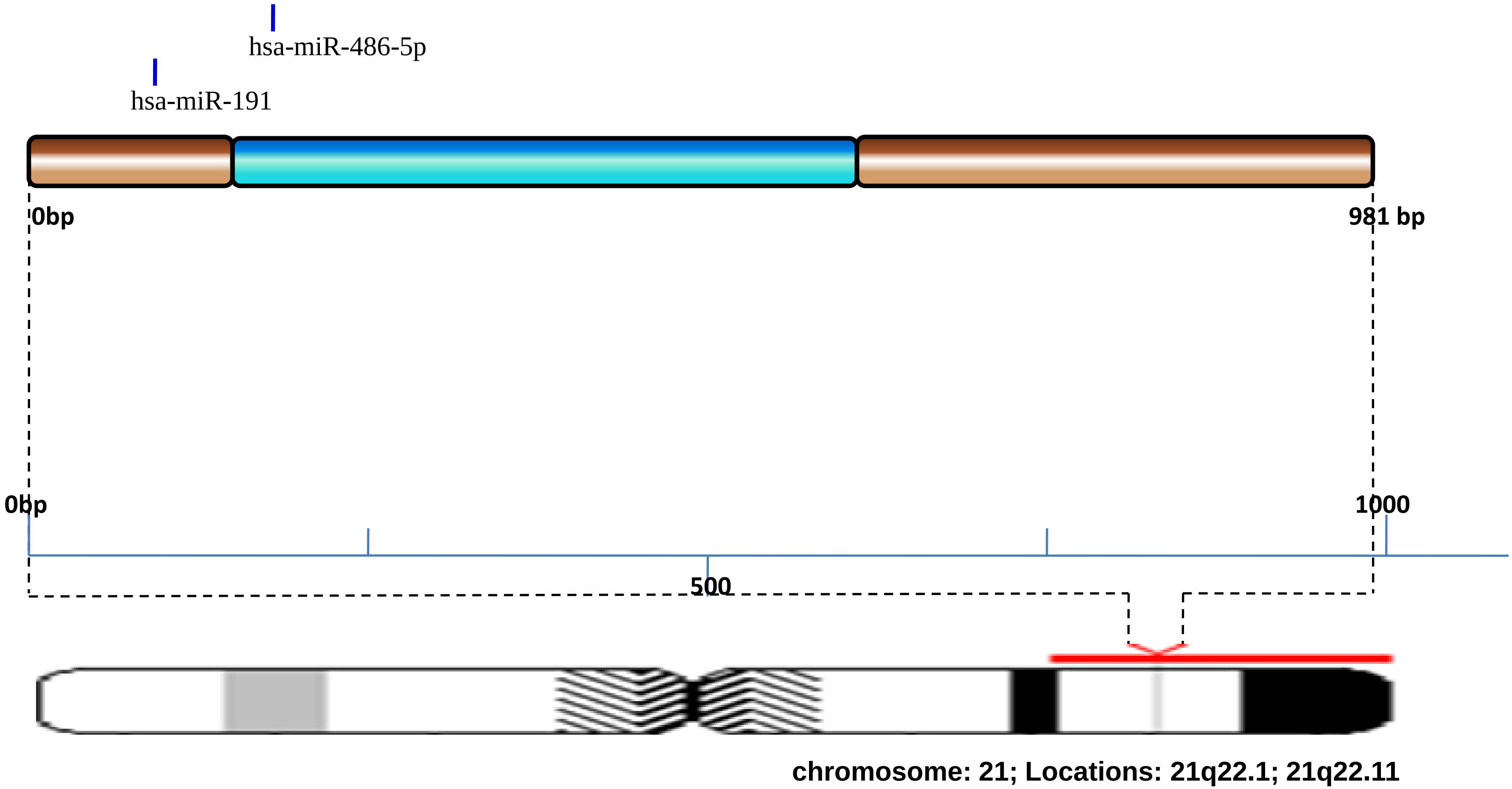

Figure SF22. Schematic representation of miRNA Target sites on SOD1

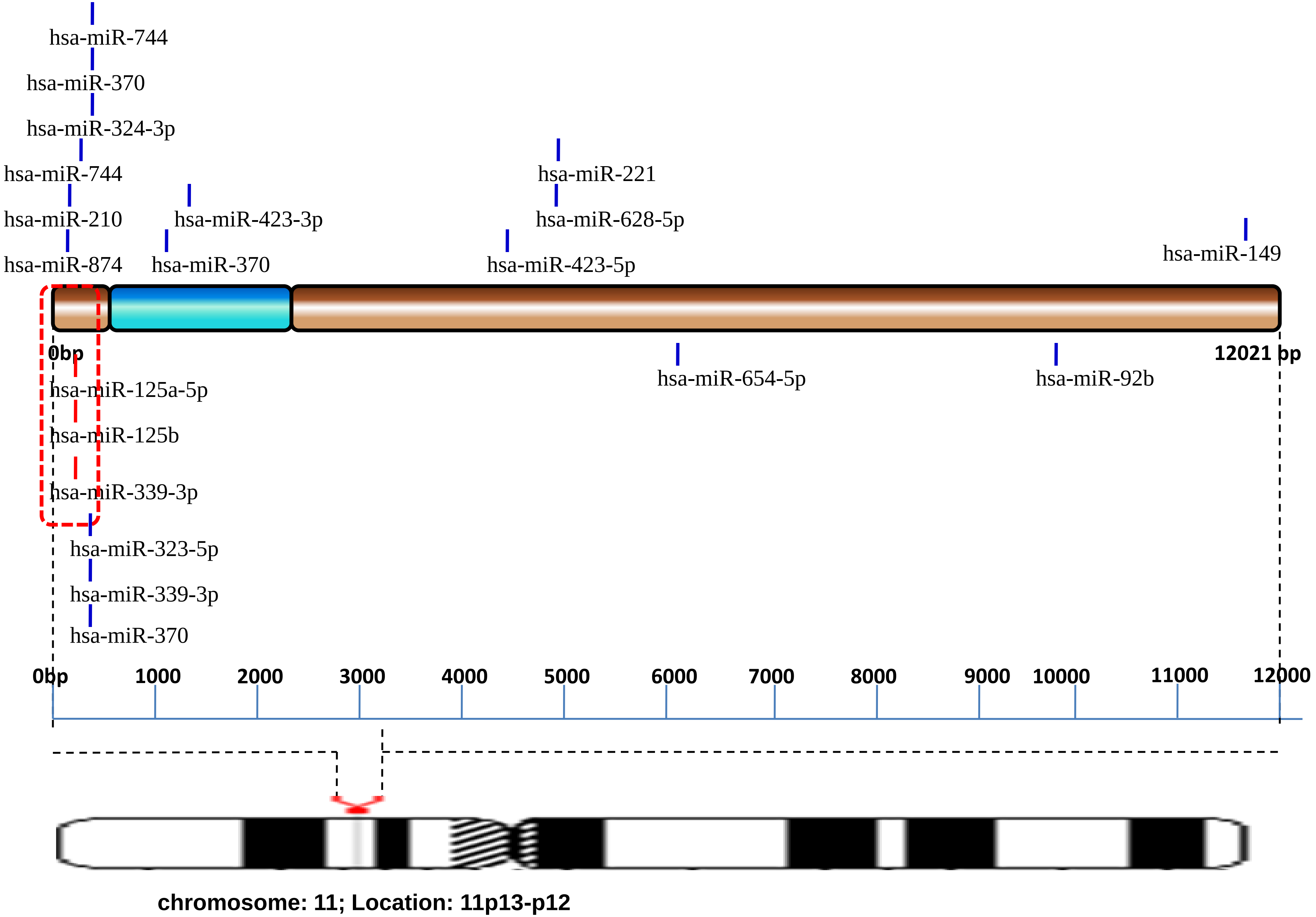

Figure SF23. Schematic representation of miRNA Target sites on SLC1A2

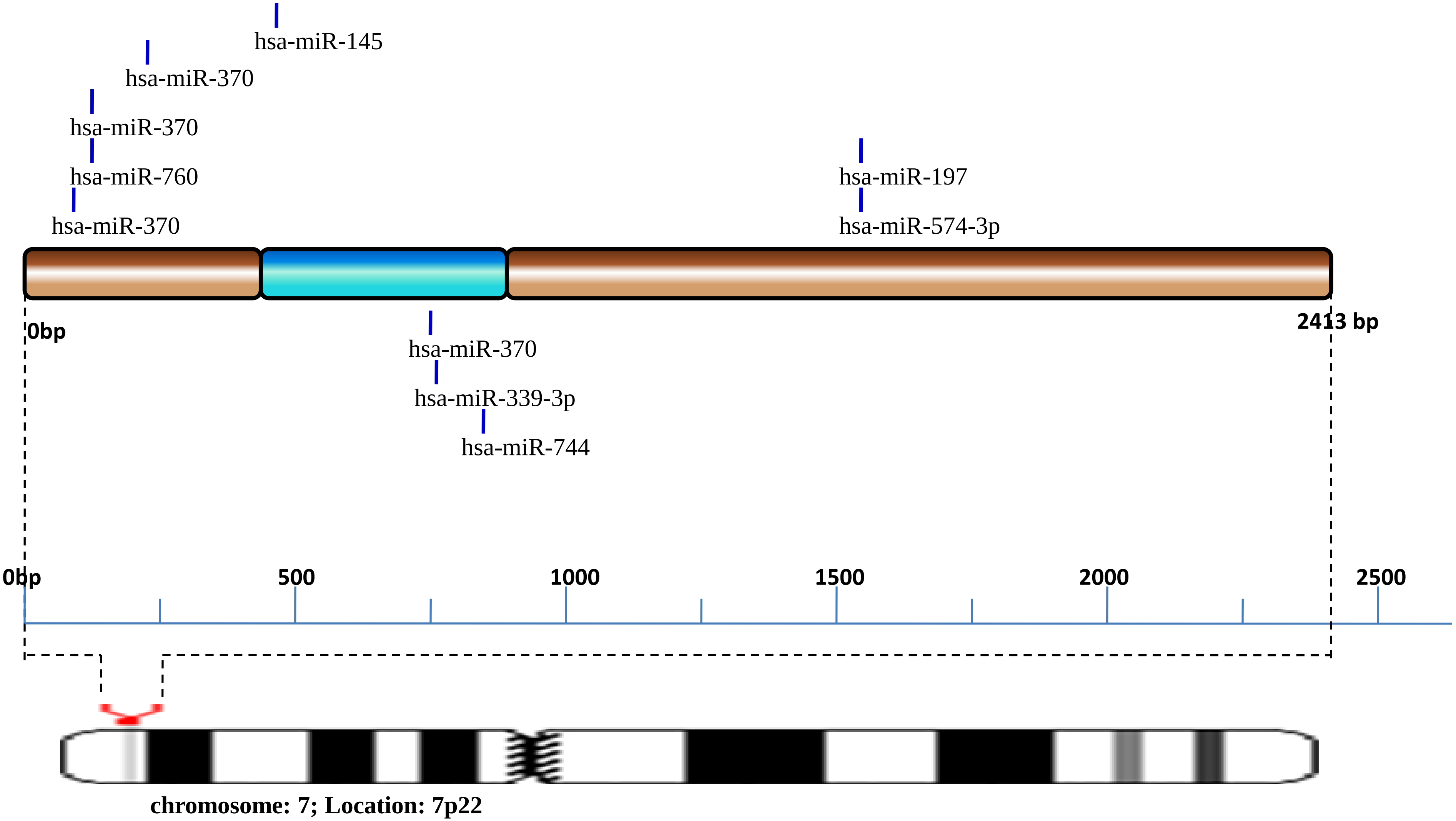

Figure SF24. Schematic representation of miRNA Target sites on RAC1

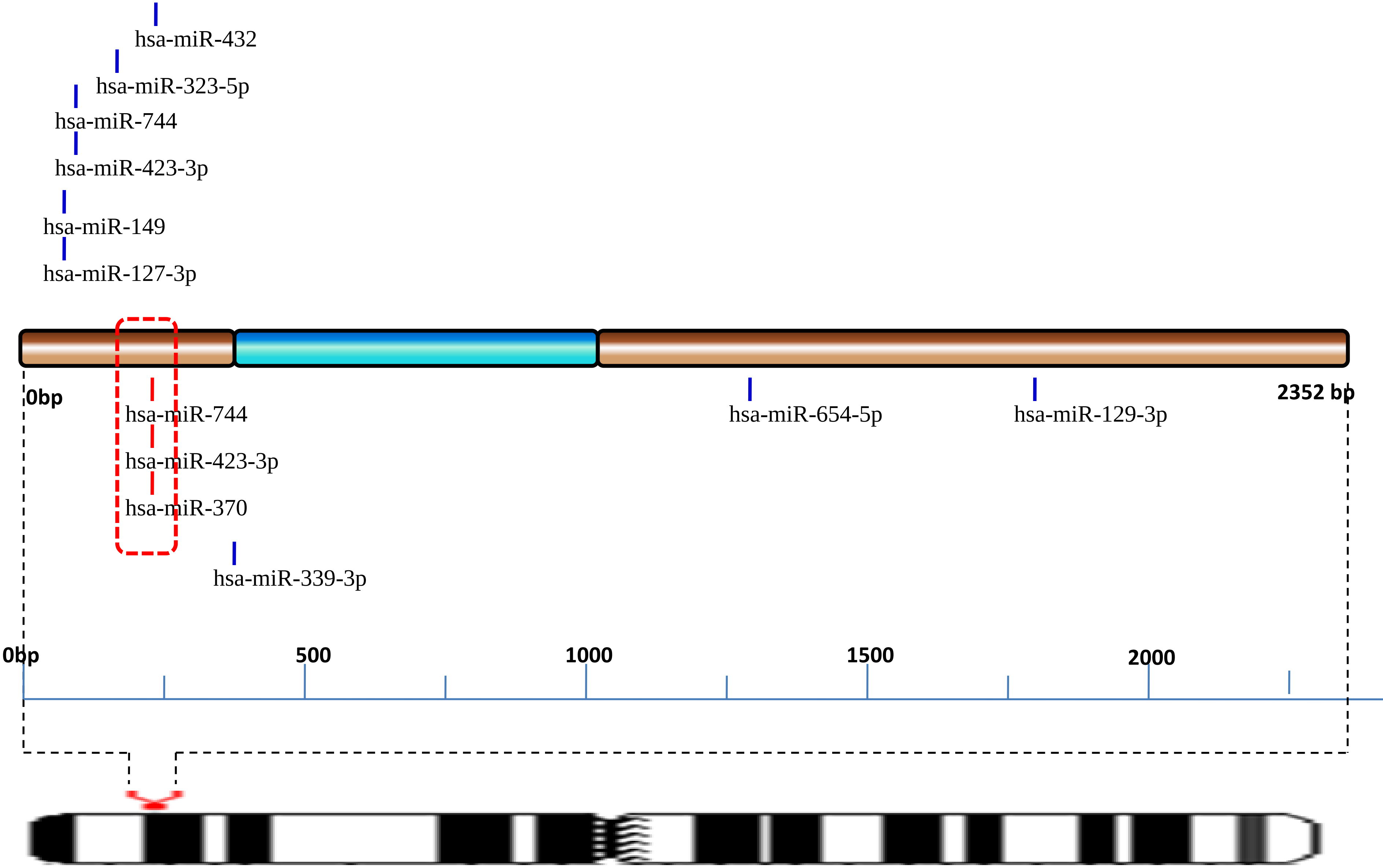

chromosome: 3; Location: 3p24-p22

Figure SF25. Schematic representation of miRNA Target sites on RAB5A

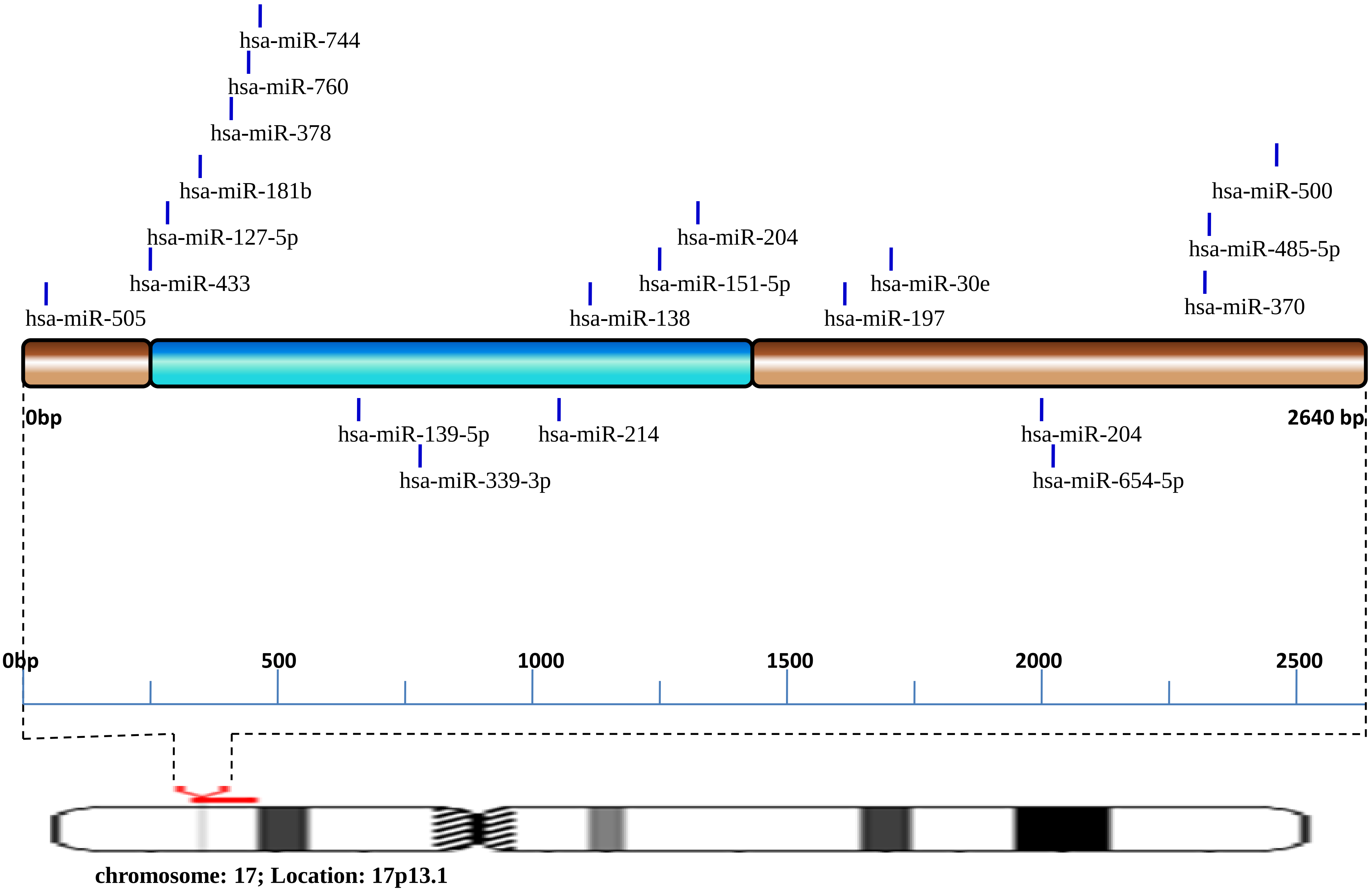

Figure SF26. Schematic representation of miRNA Target sites on p53

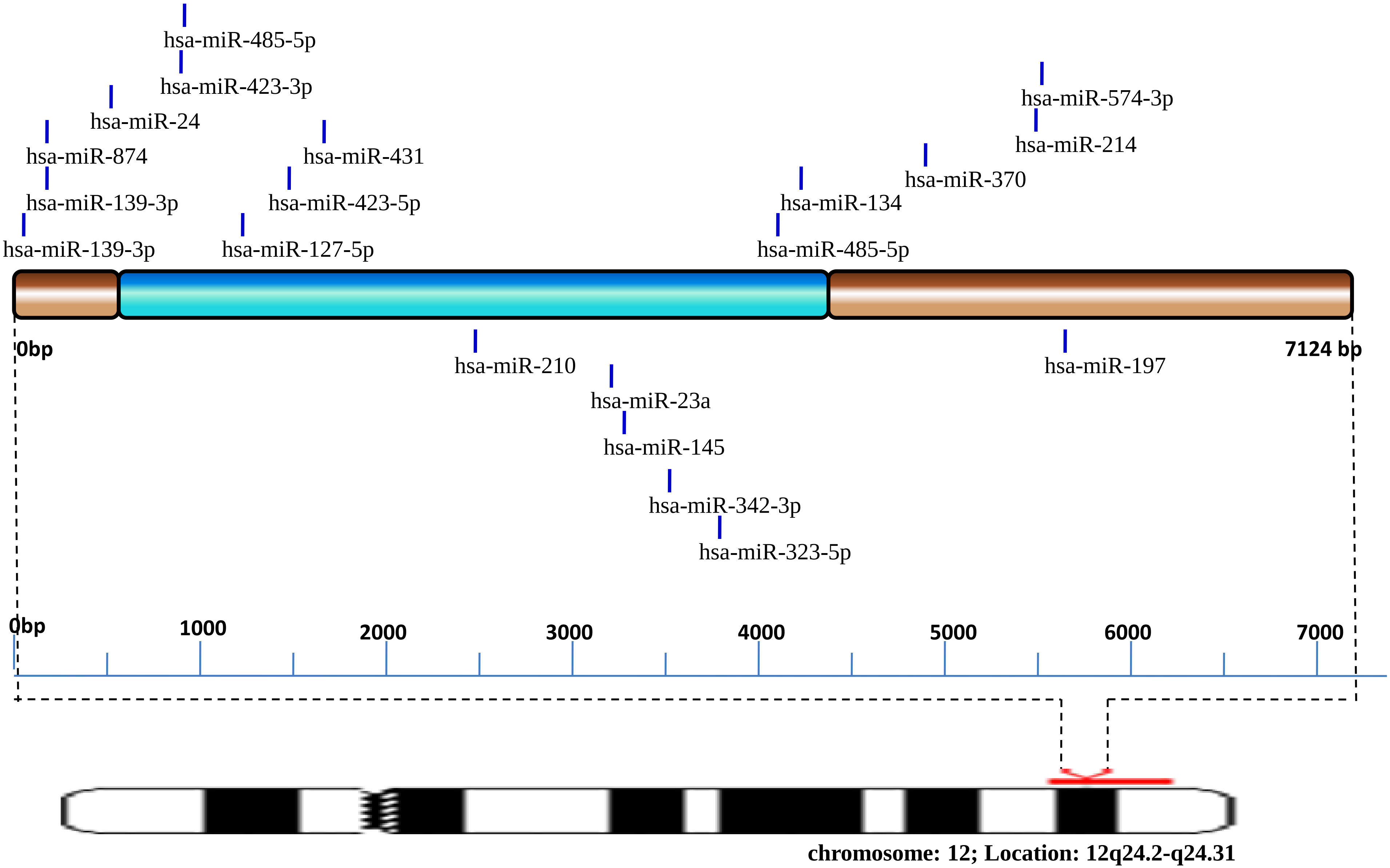

Figure SF27. Schematic representation of miRNA Target sites on NOS1

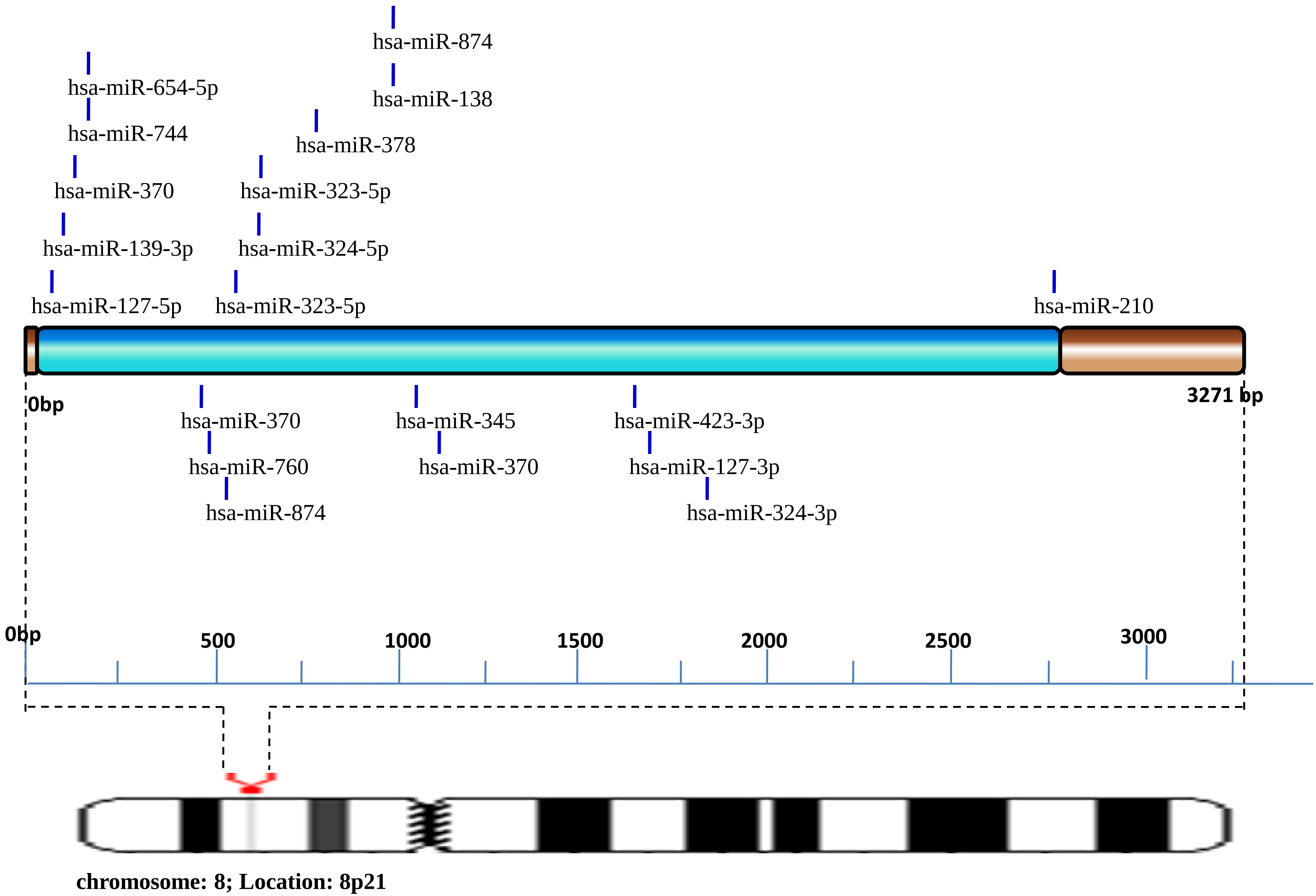

Figure SF28. Schematic representation of miRNA Target sites on NEFM

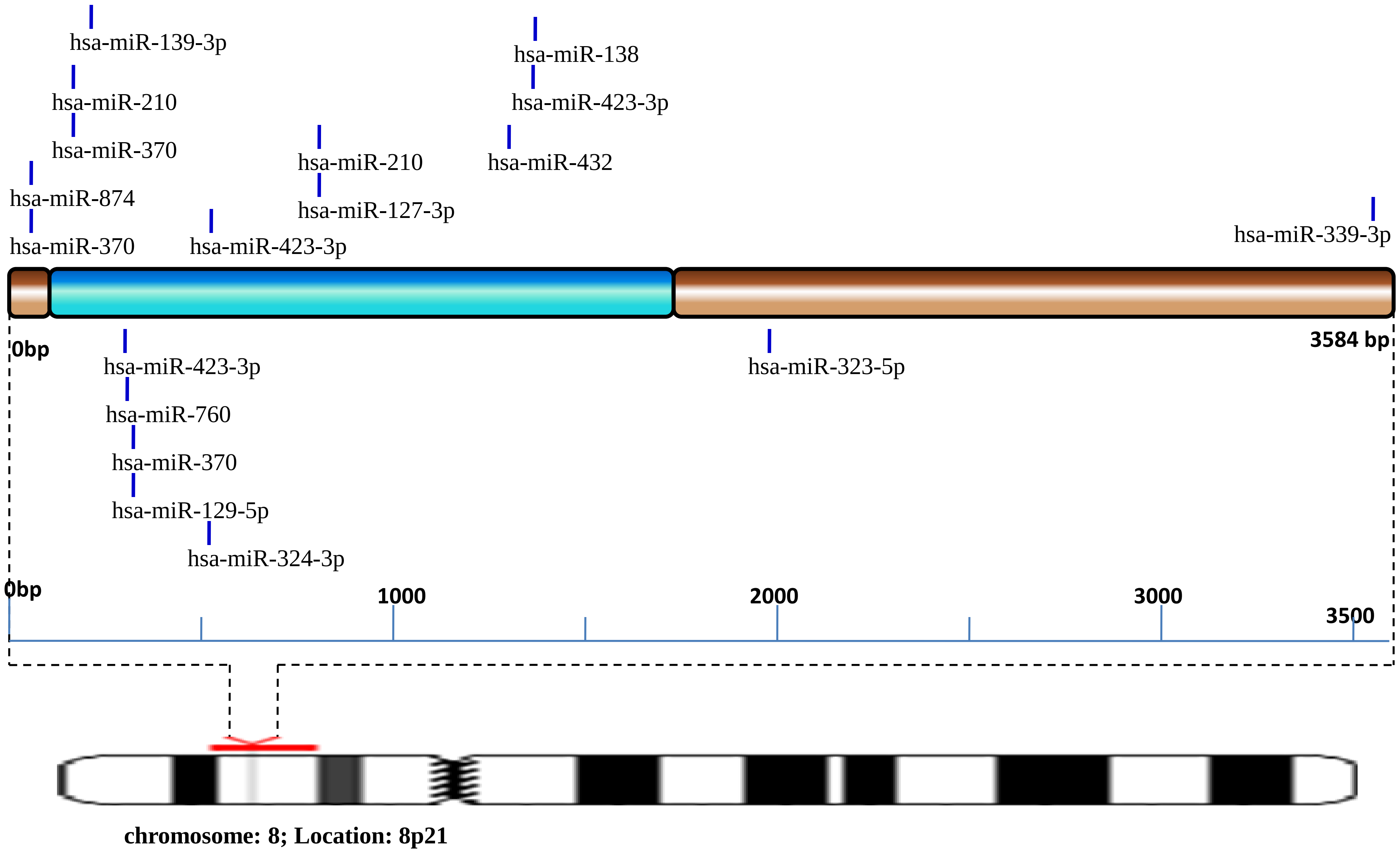

Figure SF29. Schematic representation of miRNA targets on NEFL

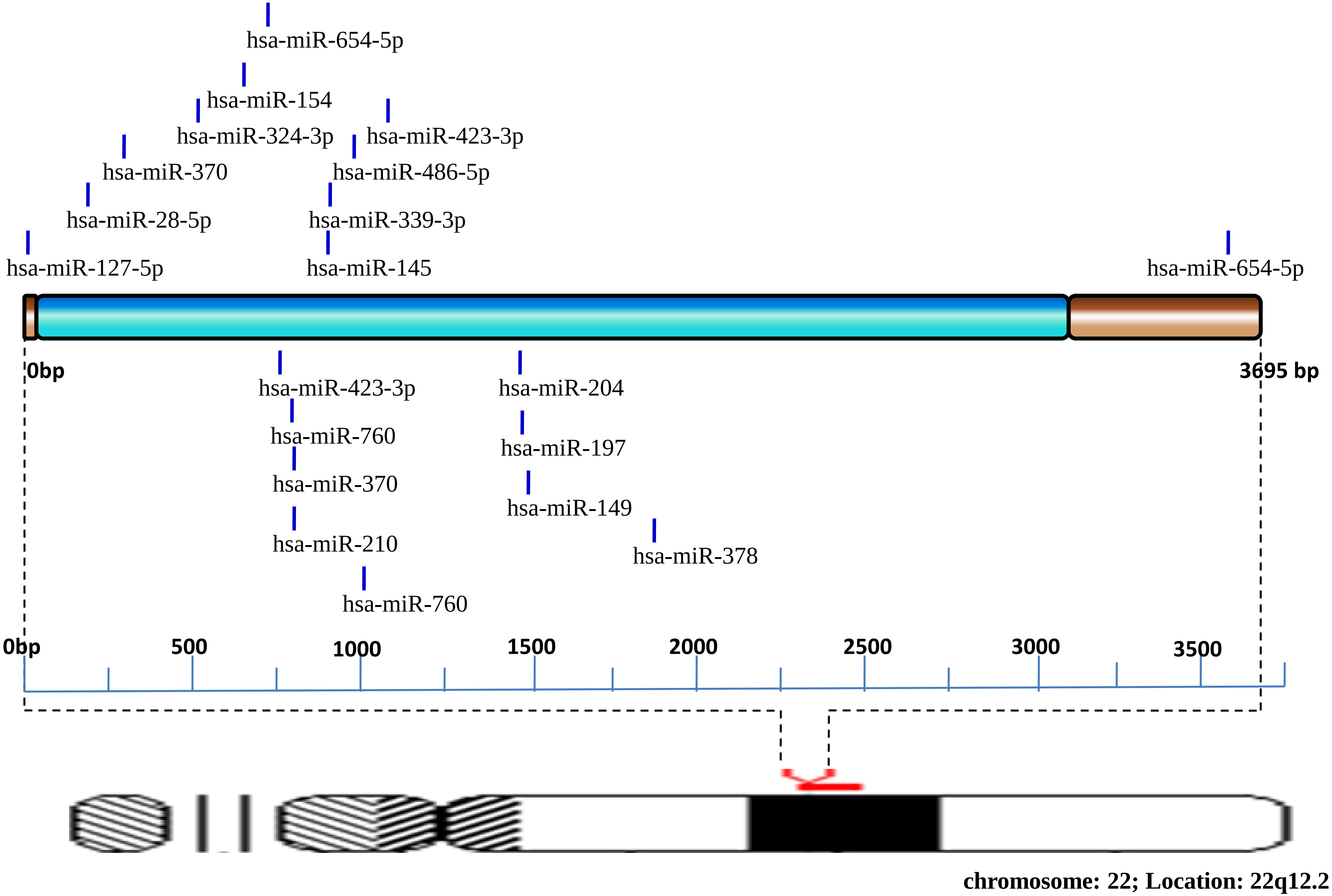

Figure SF30. Schematic representation of miRNA Target sites on NEFH

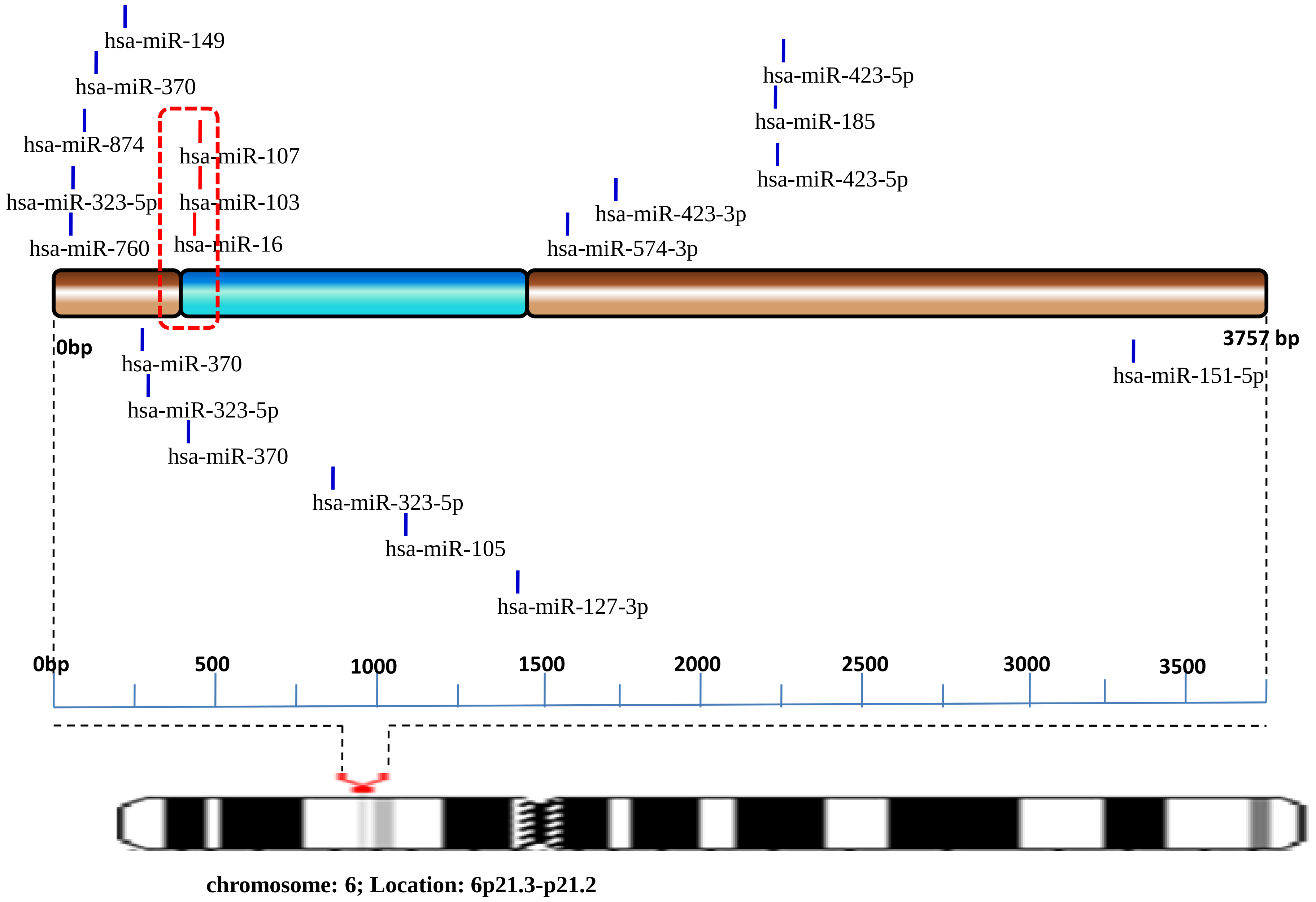

Figure SF31. Schematic representation of miRNA Target sites on MAPK14

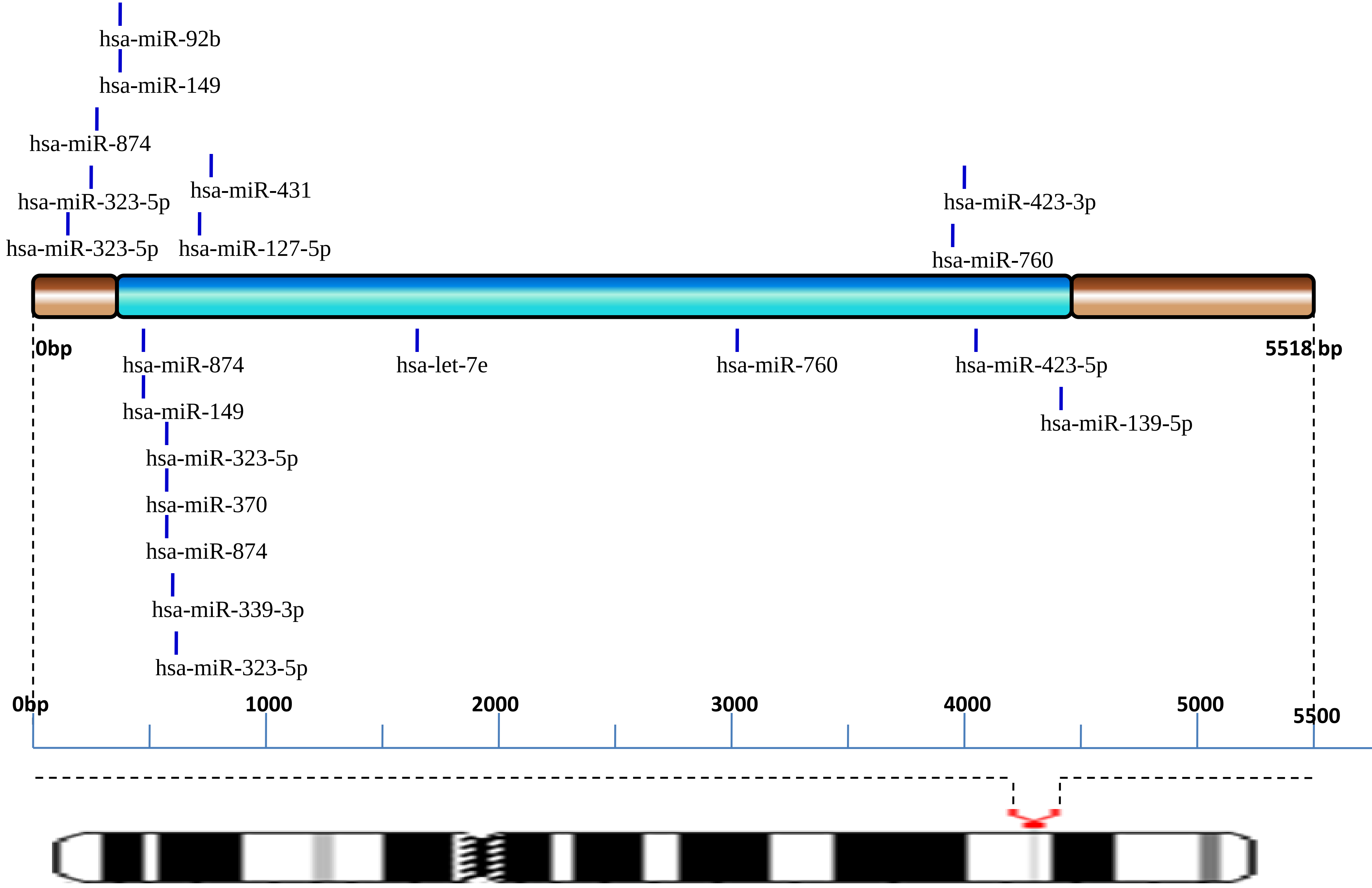

chromosome: 6; Location: 6q22.33

Figure SF32. Schematic representation of miRNA Target sites on MAP3K5

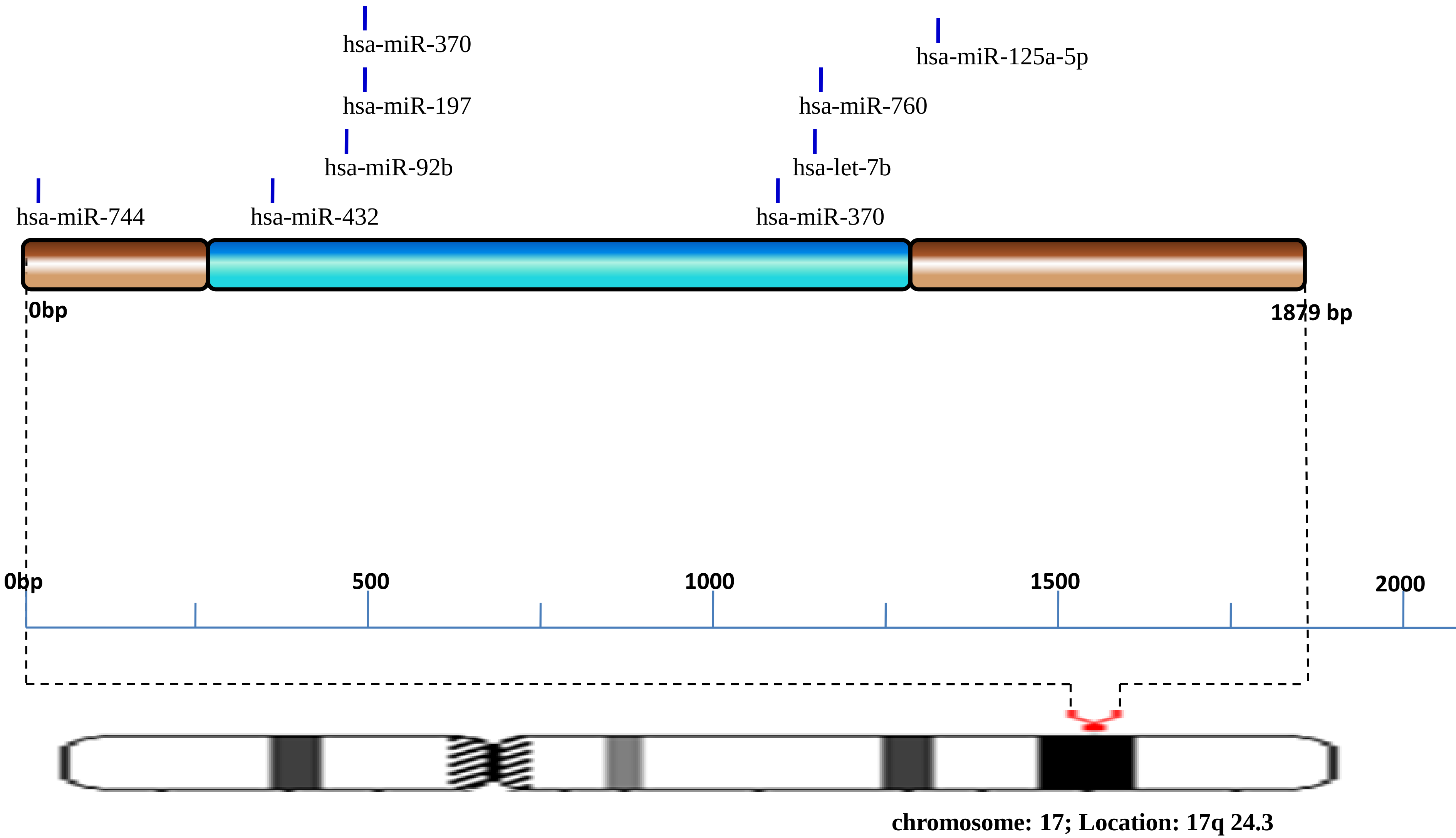

Figure SF33 Schematic representation of miRNA Target sites on MAP2K6

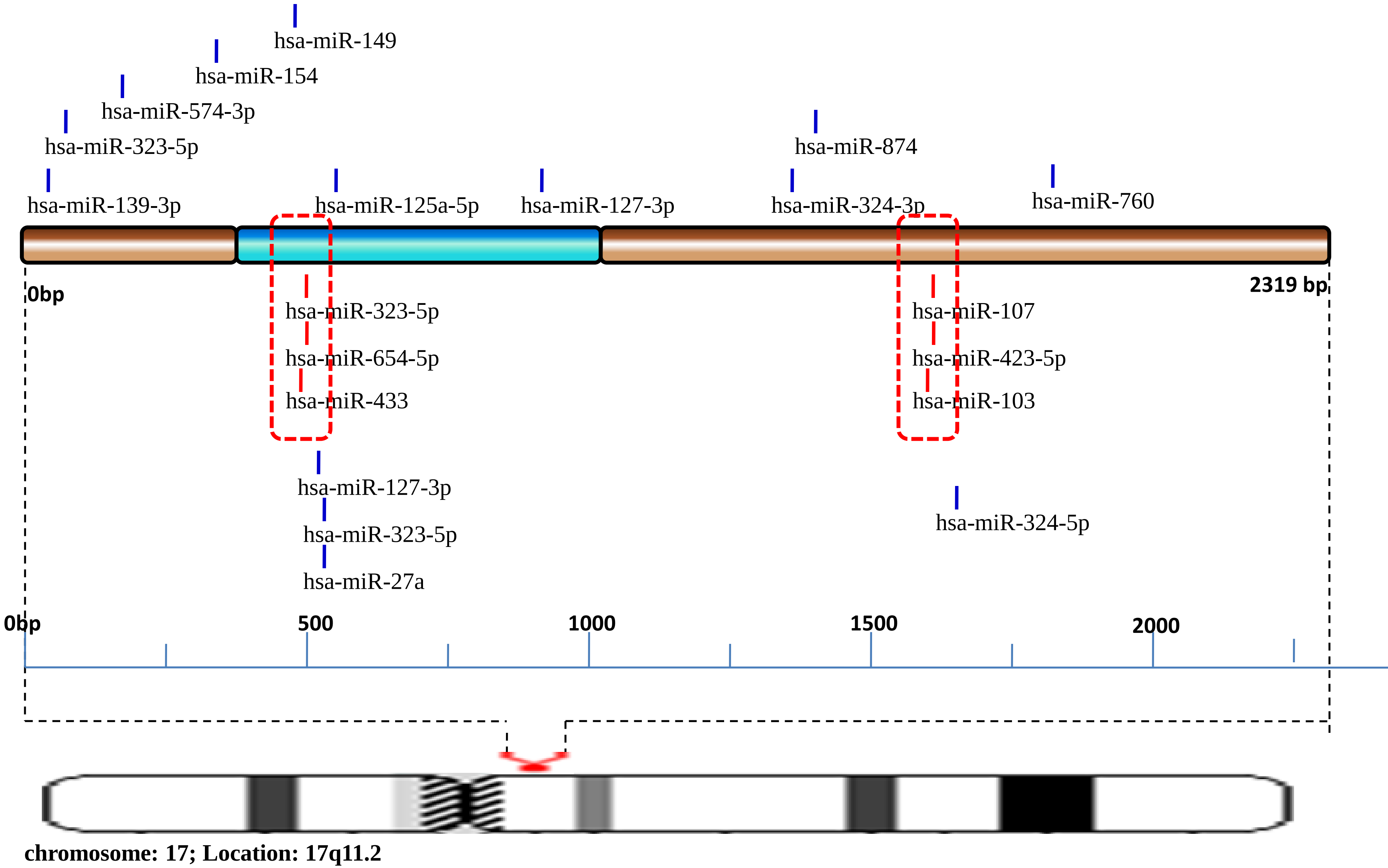

Figure SF34. Schematic representation of miRNA Target sites on MAP2K3

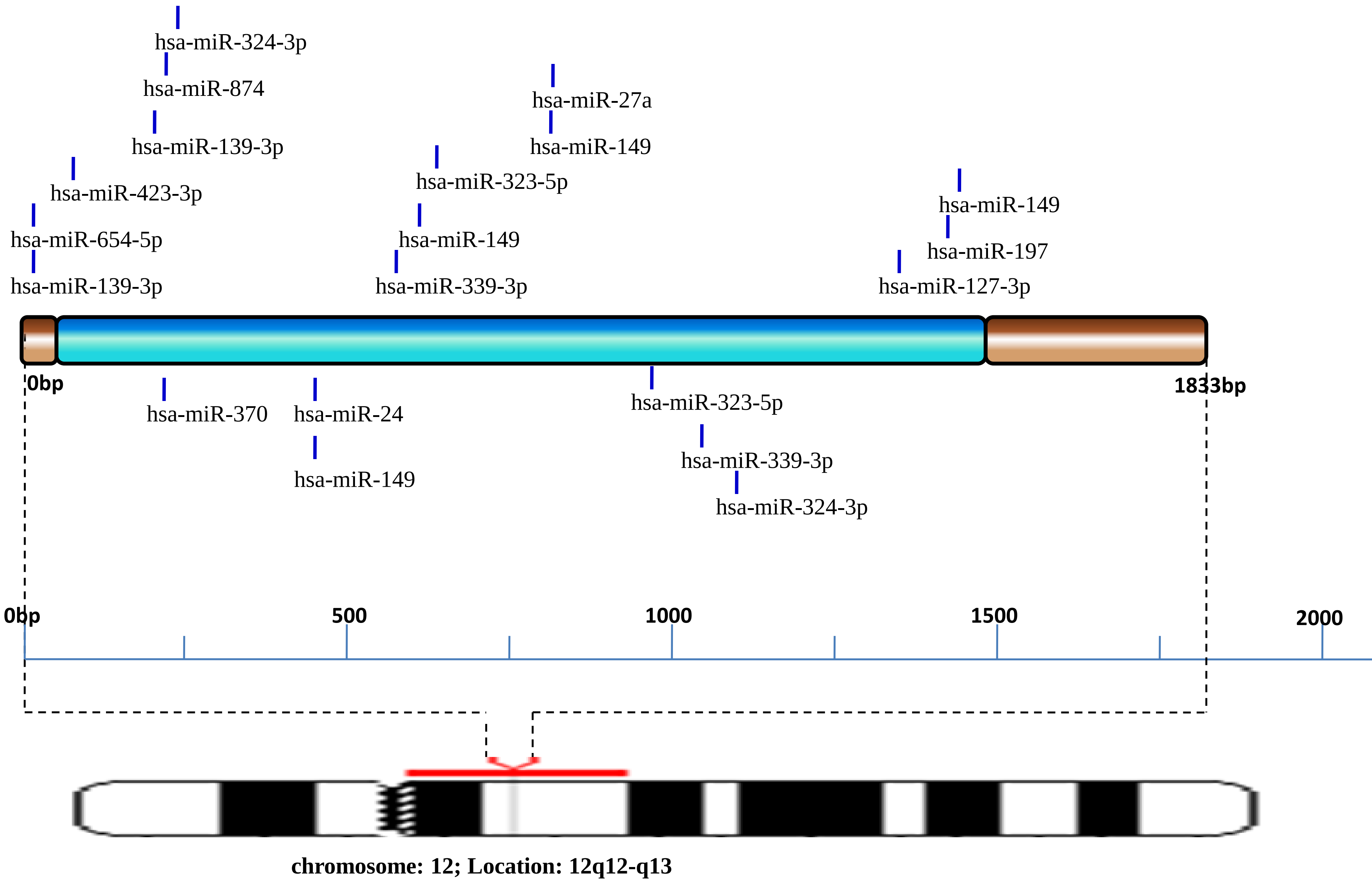

Figure SF35. Schematic representation of miRNA Target sites on PRPH

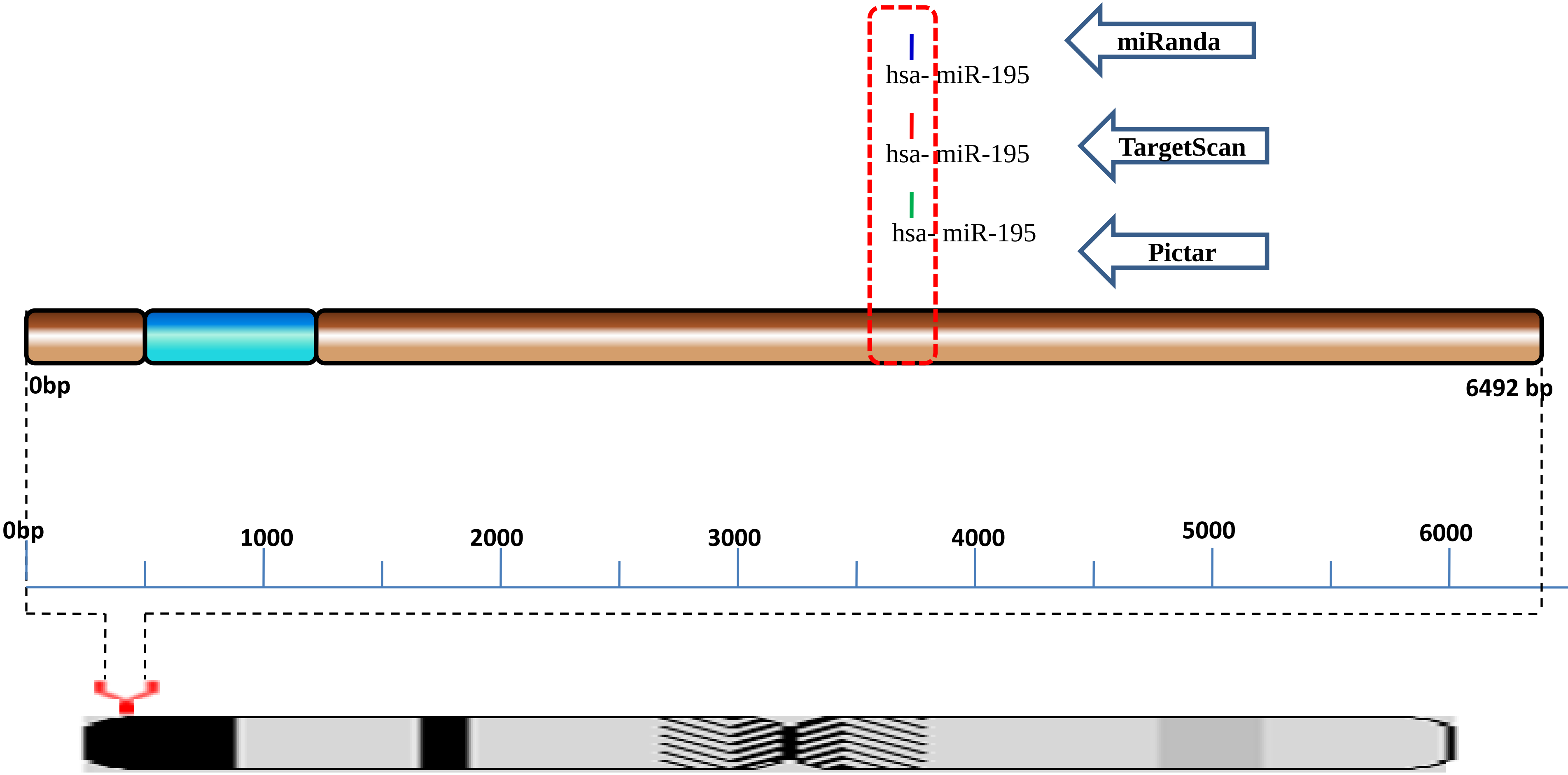

chromosome: 18; Locations: 18q21.33; 18q21.3

Figure SF36. Schematic representation of miRNA conserved target on BCL2

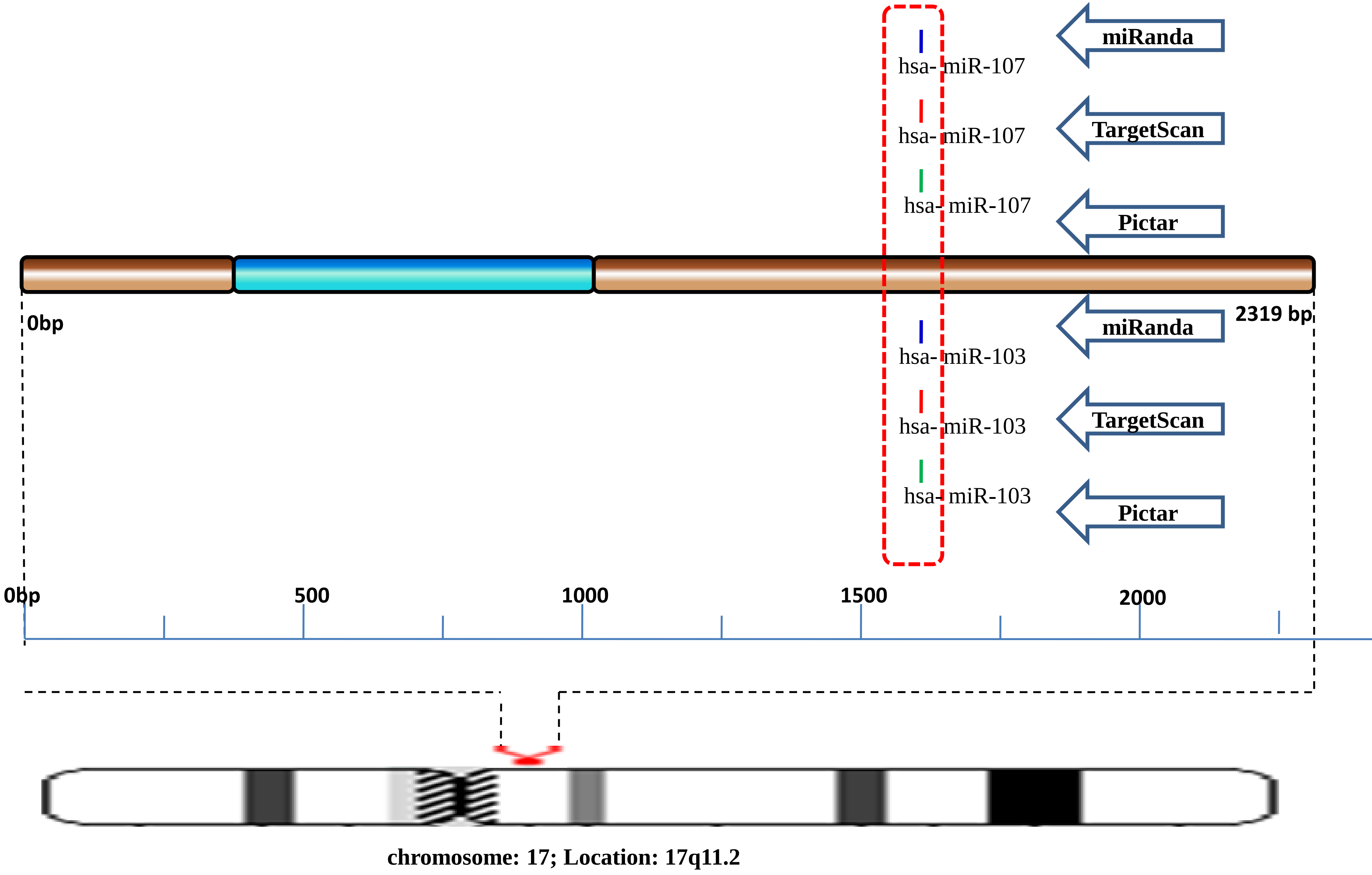

Figure SF37. Schematic representation of miRNA conserved target on MAP2K3
